# Identification of Candidate Genes Associated with Susceptibility to Ovarian Clear Cell Adenocarcinoma Using *cis*-eQTL Analysis

**DOI:** 10.3390/jcm9041137

**Published:** 2020-04-16

**Authors:** Jihye Kim, Joon-Yong Chung, Jae Ryoung Hwang, Yoo-Young Lee, Tae-Joong Kim, Jeong-Won Lee, Byoung-Gie Kim, Duk-Soo Bae, Chel Hun Choi, Stephen M. Hewitt

**Affiliations:** 1 Department of Obstetrics and Gynecology, Dankook University Hospital, Cheonan 31116, Korea; jihyeobgy.kim@gmail.com; 2 Experimental Pathology Laboratory, Laboratory of Pathology, Center for Cancer Research, National Cancer Institute, National Institutes of Health, Bethesda, MD 20892, USA; chungjo@mail.nih.gov; 3 Samsung Biomedical Research Institute, Samsung Medical Center, Sungkyunkwan University School of Medicine, Seoul 06351, Korea; jaeryounghwang@gmail.com; 4 Departments of Obstetrics and Gynecology, Samsung Medical Center, Sungkyunkwan University School of Medicine, Seoul 06351, Korea; yooyoung.lee@samsung.com (Y.-Y.L.); tj28.kim@gmail.com (T.-J.K.); garden.lee@samsung.com (J.-W.L.); bgkim@skku.edu (B.-G.K.); ds123.bae@samsung.com (D.-S.B.)

**Keywords:** ancestry, clear cell adenocarcinoma, epithelial ovarian cancer, quantitative trait loci, single-nucleotide polymorphism

## Abstract

Ovarian clear cell adenocarcinoma (Ov-CCA) has a higher prevalence in the Japanese ancestry than other populations. The ancestral disparities in Ov-CCA prevalence suggests the presence of Ov-CCA-specific genetic alterations and may provide an opportunity to identify the novel genes associated with Ov-CCA tumorigenesis. Using 94 previously reported genes as the phenotypic trait, we conducted multistep expression quantitative trait loci (eQTL) analysis with the HapMap3 project datasets. Four single-nucleotide polymorphisms (SNPs) (rs4873815, rs12976454, rs11136002, and rs13259097) that had different allele frequencies in the Japanese ancestry and seven genes associated in *cis* (*APBA3*, *C8orf58*, *KIAA1967*, *NAPRT1*, *RHOBTB2*, *TNFRSF10B*, and *ZNF707*) were identified. In silico functional annotation analysis and in vitro promoter assay validated the regulatory effect of rs4873815-TT on *ZNF707* and rs11136002-TT on *TNFRSF10B*. Furthermore, *ZNF707* was highly expressed in Ov-CCA and had a negative prognostic value in disease recurrence in our sample cohort. This prognostic power was consistently observed in The Cancer Genome Atlas (TCGA) clear cell renal cell carcinoma dataset, suggesting that *ZNF707* may have prognostic value in clear cell histology regardless of tissue origin. In conclusion, rs4873815-TT/*ZNF707* may have clinical significance in the prognosis and tumorigenesis of Ov-CCA, which may be more relevant to clear cell histology. Besides, this study may underpin the evidence that *cis*-eQTL analysis based on ancestral disparities can facilitate the discovery of causal genetic alterations in complex diseases, such as cancer.

## 1. Introduction

Gene expression levels can be considered as quantitative traits, and specific variants associated with transcript levels are referred to as expression quantitative trait loci (eQTL). eQTL analysis is a well-established in silico approach that provides evidence of the functional effects of specific loci on gene expression and identifies the novel candidate genes at the risk loci [1,2,3].

Identifying causal variants located outside of the protein coding region is an ingenious and perceptive way to discover the genetic landscape of complex diseases, including cancer [4]. There are two major applications for using eQTL analysis to investigate the genetic landscape in complex diseases [1]. Genome-wide association study (GWAS) is a well-established and effective method of identifying candidate risk loci associated with specific traits in large cohorts. The majority of candidate risk loci identified by GWAS are mainly located in the noncoding region and are assumed to be non-causal [5]. eQTL analysis can measure gene expression of these risk loci to identify novel susceptibility loci and associated genes that can be involved in gene regulation related to a specific trait [1]. Several studies on cancers, including epithelial ovarian carcinoma (EOC), have identified novel genes and proposed molecular pathways through eQTL analysis using the identified risk loci in GWAS [6,7,8]. In addition, using well-known genetic mutations or disease-specific characteristics as the phenotypic trait, several studies with eQTL analysis have already identified specific risk loci and associated novel candidate genes in multiple cancer types, including breast, prostate, lung, colorectal, and high-grade serous ovarian carcinoma (HGSOC) [3,9,10,11].

Epithelial ovarian carcinoma (EOC) is the second most common gynecologic cancer and the leading cause of death for patients with gynecologic cancer in developed countries [12]. Ovarian clear cell adenocarcinoma (Ov-CCA) is the second most common subtype of EOC and is characterized by resistance to conventional platinum-based chemotherapy, which causes poor prognosis [12,13,14,15]. In addition, a significant ethnic difference exists in the prevalence of Ov-CCA between Western and Japanese populations. Ov-CCA is a rare tumor in Europe and the United States (1–12%), whereas the prevalence in Japan is as high as 15–25% compared to non-Japanese Asian populations [14,16]. This ethnic difference is also observed in Japanese living in the United States. They exhibit a markedly higher incidence of Ov-CCA than Caucasians (3.4% vs. 9.0%) [12]. While socioeconomic factors have significant relevance in these disparities, ethnic difference in Ov-CCA prevalence suggests the presence of Ov-CCA-specific genetic alterations associated with ancestry. In this regard, recent advances in breast and prostate cancer research have demonstrated that genetic factors related to ancestry may be associate with tumorigenesis [17,18].

In this study, we conducted *cis*-eQTL analyses to identify candidate susceptibility loci using 94 previously reported genetic mutations in Ov-CCA as the phenotypic trait [19]. Given that disease-specific ancestral disparities can be used as the phenotypic trait in eQTL analysis [17,18], we hypothesized that specific susceptibility loci and its associated genes can be identified in Ov-CCA by Japanese ancestry. Lastly, to validate potential functional role of the identified novel susceptibility loci and associated genes in Ov-CCA, we performed expression and survival analysis of the identified genes in both our gene expression datasets and TCGA (The Cancer Genome Atlas) datasets.

## 2. Materials and Methods

### 2.1. Identification of Susceptibility Loci and Associated Genes through cis-eQTL Analysis

In a previous study, we had performed an integrative analysis using array comparative genomic hybridization (aCGH) and paired gene expression microarray data on 19 freshly frozen Ov-CCA samples. With this, 94 candidate genes were identified with frequent copy number alterations in Ov-CCA [19].

**Figure 1:**
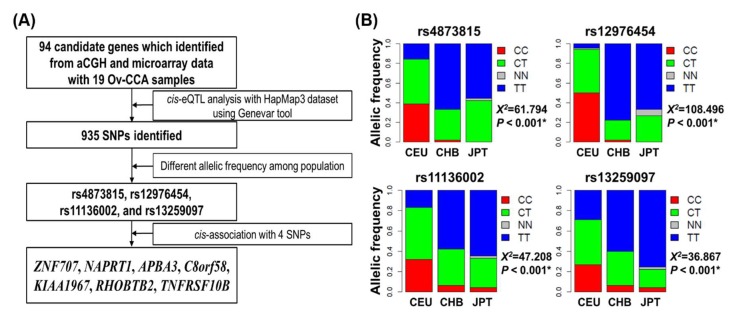
Identification of Susceptibility Loci and Associated Genes through cis-eQTL Analysis. (**A**) Flowchart of the study. (**B**) Four single-nucleotide polymorphisms (SNPs) differentially distributed among European (CEU), Chinese (CHB), and Japanese (JPT) populations. *p*-values < 0.001 are flagged with asterisk (*).

In this study, a three-step eQTL analysis was employed to identify susceptibility loci and related genes associated with Japanese ancestry. We used gene expression profiling and genotyping data from the HapMap3 project, which included 1099 individuals from eight ancestries (Table S1) [20]. The detailed workflow is described in Figure 1A.

First, with a set of 94 candidate genes treated as the phenotypic trait, the eQTL analysis (http:www.gtexportal.org/home/eqtl) was performed to identify specific single-nucleotide polymorphisms (SNPs) in genes of interest with the HapMap3 dataset using the Genevar tool (Wellcome Sanger Institute, Cambridge, UK, http://www.sanger.ac.uk/resources/software/genevar) [21]. We excluded SNPs if their imputation accuracy was r^2^ < 0.25. Allele frequencies for each SNP were also computed, and SNPs with minor allele frequencies (<0.1) were excluded from further analysis, as were SNPs that did not satisfy the Hardy–Weinberg equilibrium with *p* < 10^−4^. A total of 935 SNPs passed the filtering step (Table S2). Second, of the 935 SNPs, we sought those that were preferentially prevalent in the Japanese ancestry (JPT) in comparison to the European (CEU) or Chinese (CHB) ancestries. Four SNPs (rs4873815, rs12976454, rs11136002, and rs13259097) fit these criteria (Figure 1B). Lastly, to identify the gene associated with each of the four SNPs, we identified the association between the corresponding germline genotypes and the transcript abundances of mRNAs within 500 kb of either side of the corresponding loci based on linear regression coefficients. A *cis*-association was determined by significant correlation between the germline genotypes and the transcript levels at a false discovery rate (FDR) of 0.05. *p*-values were adjusted using the Benjamini–Hochberg method for multiple testing corrections. Through these workflows, seven associated genes (*ZNF707*, *NAPRT1*, *APBA3*, *C8orf58*, *KIAA1967*, *RHOBTB2*, and *TNFRSF10B*) were demonstrated (Figure 1A).

### 2.2. Functional Validation of Susceptibility Loci on Associated Genes

We performed an in silico functional analysis on four SNPs using Ensembl Variant Effect Predictor (VEP) analysis with Combined Annotation-Dependent Depletion (CADD) Phred score and LoFtool annotation tools to annotate and prioritize variants for further promoter assay [22,23,24].

Based on the results of in silico functional analysis, rs4873815/*ZNF707* was selected for in vitro promoter assay. In addition, because rs11136002 was associated in *cis* with four genes among the seven identified genes, rs11136002/*TNFRSF10B* was also selected for promoter assay as representative.

In the multistep eQTL analysis of this study, alleles TT of rs4873815 and rs11136002 were most frequently expressed in Japanese ancestry. Accordingly, promoter assay was performed with the alleles TT of rs4873815 and rs11136002, which were expected to have regulatory effects on *ZNF707* and *TNFRSF10B*, respectively. Allele CC of *ZNF707* and *TNFRSF10B*, which had a significantly lower allelic frequency in JPT than CEU and CHB, were selected as control. In addition, each internal control vector contained only *TNFRSF10B* promoter or *ZNF707* promoter, as in a previous study [25].

We conducted dual-luciferase reporter assays to investigate whether the promoter activity of *ZNF707* and *TNFRSF10B* was regulated by rs4873815 and rs11136002, respectively. Promoter regions of *ZNF707* and *TNFRSF10B* were amplified by PCR using genomic DNA from human embryonic kidney 293T cells (HEK293T).

The primers used for PCR were as follows:

*TNFRSF10B*-F: 5′-CCAAGAGCTCGCACCCGGGCCAGCGGCTG-3′

*TNFRSF10B*-R: 5′-CCGGAGATCTGGCGTCATTCGGGGCGGGGC-3′

*ZNF707*-F: 5′-CCGGGAGCTCGTGAGGACTAAACTGATC-3′

*ZNF707*-R: 5′-CAGGAAGCTTCCGGCCCCTCTAGGAG-3′

The 2000 base pairs containing promoter regions for *ZNF707* and *TNFRSF10B* of PCR products were cloned at SacI and BglII for *TNFRSF10B* and SacI and HindIII sites for *ZNF707* in a pGL3-basic firefly luciferase reporter plasmid (Promega Corporation, Madison, WI, USA). About 500 base pairs around the SNP region in rs11136002 and rs4873815 were amplified by PCR with genomic DNA isolated from the RMG-2 ovarian clear cell carcinoma cell line. The PCR products were cloned at the KpnI and SacI sites in a pGL3-basic plasmid containing *TNFRSF10B* promoter or *ZNF707* promoter, respectively. The primers used for amplifying rs11136002 and rs4873815 genes were as follows:

rs1136002-F: 5′-CCGGGGTACCCACATCTGATCAAGGATTG-3′

rs1136002-R: 5′-CCGGGAGCTCGGAAACGATGAATACAGC-3′

rs4873815-F: 5′-CCGGGGTACCCTCAATATTGTTATATCC-3′

rs4873815-R: 5′-CCAAGAGCTCCACATCACATTTTTCTC-3′

The constructs were confirmed by Sanger method with BigDye^®^ Terminator v3.1 cycle sequencing kit from SolGent Co., Ltd. (Daejeon, Korea). The sequences were analyzed by ABI 3730XL DNA analyzer (50 cm capillary). Each primer used for PCR reactions (described above) were used for sequencing of rs11136002 and rs4873815 genes. For sequencing *TNFRSF10B* promoter or *ZNF707* promoter, inner primers were used as well as the primers used for PCR reactions because these promoters were about 2000 base pairs. The inner primers and the sequencing results are provided in Table S3.

For the promoter assay, each firefly luciferase reporter plasmid and Renilla luciferase reporter plasmid (pRL-SV40: Promega), an internal control for transfection efficiency, was transfected into the HEK293T cells. Sixteen hours after transfection, luciferase activity was measured by a dual-luciferase assay kit (Promega, Madison, WI, USA). Three biological replicates in triplicate were performed for the experiments to ensure reproducibility.

### 2.3. Analysis of the Clinical Significance of the Seven Identified Genes Using Our Sample Cohort

For validation of the four SNPs and seven associated genes in Ov-CCA, we first analyzed gene expression microarray data from our previous studies to investigate whether these genes were expressed differentially among Ov-CCA (*n* = 19), HGSOC (*n* = 26), and normal ovarian tissue (*n* = 7) [19,26]. The samples were collected prospectively from patients at Samsung Medical Center with Institutional Review Board (IRB) approval (2011-04-008, Seoul, South Korea) and informed consent. All the patients were treated with maximal debulking surgery followed by cisplatin-based combination chemotherapy. Optimality status was defined according to the size of the nodules remaining after surgery (<1 cm, optimal; ≥1 cm, suboptimal). Gene expression analyses of the HGSOC and normal ovarian tissue were performed using AffymetrixGeneChip Human Gene 1.0 ST oligonucleotide arrays (Affymetrix, Santa Clara, CA, USA, http://www.affymetrix.com). Analysis of the Ov-CCA was performed using the Agilent oligo microarray kit 8x60K according to the Agilent One-Color Microarray-Based Gene Expression Analysis Protocol (Agilent Technologies, Santa Clara, CA, USA, http://www.chem.agilent.com). To avoid the batch effect while maintaining biological variability, we performed quantile normalization for each method prior to batch effect correction. We used in silico Merging with Combat for batch effect correction (r/bioconductor packages) [27].

In addition, a Kaplan–Meier survival curve was plotted to investigate whether expression of seven genes was significantly associated with survival in HGSOC or Ov-CCA patients. In addition, multivariate Cox proportional hazards regression models were employed to evaluate the prognostic power of the identified genes in HGSOC and advanced-stage Ov-CCA.

### 2.4. Analysis of the Clinical Significance of the Seven Identified Genes Using TCGA Datasets

To further elucidate the clinical significance of the identified genes, we conducted survival analysis using gene expression profiles from the publicly available TCGA database (https://tcga-data.nci.nih.gov/tcga/tcgaHome2.jsp). As there were no Ov-CCA cases in the TCGA database, a clear cell renal cell carcinoma (TCGA-ccRCC) dataset, reported to have similar molecular profiles to that of Ov-CCA, was included in the survival analysis as an alternative [28]. In addition, the HGSOC dataset was included as a control.

Clinical information and molecular data, including mRNA expression, were obtained from the TCGA Data Portal. This study meets the publication guidelines provided by TCGA (http://cancergenome.nih.gov/publications/publicationguidelines).

### 2.5. Statistical Analysis

For promoter assay, statistical significance was measured using unpaired Student’s *t*-test. After confirming whether the data were normally distributed using the Shapiro–Wilk test, we used the Wilcoxon rank sum test to compare gene expressions among our sample cohorts. For survival analysis, expression values of a gene were dichotomized into high and low using the median as a cutoff. Overall survival (OS) was defined as the interval from the date of initial treatment to the date of last contact or death. Progression-free survival (PFS) was defined as the interval from the date of initial treatment to the date of progression, date of recurrence, or the date of last contact. PFS and OS curves were created with the Kaplan–Meier method and the log-rank test. All of the tests were two-sided, with statistical significance set at *p* < 0.05, and were performed using R software, version 3.1.3 (R Foundation, Vienna, Austria; http://www.R-project.org).

## 3. Results

### 3.1. Total of 935 SNPs Identified through cis-eQTL Analysis of 94 Genes Reported in Ov-CCA

eQTL analysis of 94 Ov-CCA-associated genes revealed a total of 935 eQTLs with significant correlations after FDR adjustment (FDR, 0.05) (Table S2). SNPs appeared most frequently on chromosomes 8 (628 SNPs) and 17 (174 SNPs). The three most frequent ancestries showing SNPs were CEU (200 SNPs), Maasai (MKK, 124 SNPs), and Luhya (LWK, 106 SNPs), while JPT only had 68 correlated SNPs (Table S1).

### 3.2. Four cis-eQTL with Differential Allelic Frequency in the Japanese Ancestry

Of the 935 identified *cis*-eQTLs, four SNPs (rs4873815, rs12976454, rs11136002, and rs13259097) had significantly different frequencies of alternative alleles among CEU, CHB, and JPT populations (x^2^ = 61.79, 108.50, 47.21, and 36.87, all of *p* < 0.001, respectively) (Figure 1B). Details of the difference in frequencies between CHB and JPT are summarized in Table S4.

### 3.3. Seven Genes Associated with Four SNPs in cis

Next, we identified the seven associated genes with statistically significant *cis*-eQTL associations with the four SNPs using the HapMap3 dataset. As shown in Table 1, rs4873815 was located on chromosome 8q24.3 and identified as a *cis*-eQTL for genes *ZNF707* (*p* = 8.92e−4) and *NAPRT1* (*p* = 4.41e−4). rs11136002 was located on chromosome 8p21.3 and identified as a *cis*-eQTL for *C8orf58* (*p* = 5.53e−4), *KIAA1967* (*p* = 9.19e−4), *RHOBTB2* (*p* = 3.12e−4), and *TNFRSF10B* (*p* = 4.03e−4). rs12976454 was located on chromosome 19p13.3 and identified as a *cis*-eQTL for *APBA3* (*p* = 3.40e−4). rs13259097 was located on chromosome 8p21.3 and identified as a *cis*-eQTL for *RHOBTB2* (*p* = 3.87e−4).

Of these, *C8orf58* was first identified in EOC through this study. *NAPRT1*, involved in the nicotinamide adenine dinucleotide (NAD) synthesis pathway, was not reported in our previous study of Ov-CCA. The remaining five genes were previously reported in a study that identified 94 candidate genes [19].

### 3.4. Potential Regulatory Effect of rs4873815-TT and rs11136002-TT on Associate Genes

In the VEP analysis, we annotated the affected transcripts of four SNPs and prioritized them based on CADD Phred scores and LoFtool toolsets. As shown in Figure S1, rs4873815 had a 12.08 CADD Phred score and 0.627 LoFtool score for the *ZNF623* gene located near the *ZNF707* gene, indicating that it is a possibly deleterious and phenotypically functional variant in an individual. In addition, because rs11136002 was associated in *cis* with four genes among seven identified genes, rs11136002/*TNFRSF10B* was selected for the promoter assay as representative.

To verify whether identified susceptibility loci affect promoter activity of associate genes, luciferase reporter assays were performed in HEK293T cells using rs4873815 and its candidate gene *ZNF707* or rs11136002 and *TNFRSF10B*.

**Figure 2:**
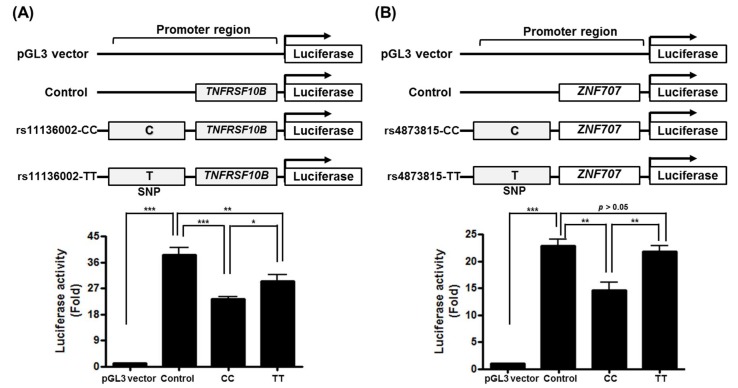
Promoter activity of (**A**) rs11136002/*TNFRSF10B* and (**B**) rs4873815/*ZNF707*. Compared to the pGL3 vector, Allele CC and Allele TT exhibited significant induction of promoter activities in each locus. Between the two allele constructs (CC vs. TT), the transcriptional activity of TT was significantly higher than that of CC in both loci, implying that the transcription regulation is allele-specific in both genes. Each bar represents mean ± SEM of firefly luciferase activity normalized to Renilla luciferase activity. * *p* < 0.05, ** *p* < 0.01, *** *p* < 0.001.

As shown in Figure 2, transcriptional activity with the T allele of rs4873815 (rs4873815-TT), which showed a high frequency in JPT, was significantly higher than that with the C allele of rs4873815 (rs4873815-CC, high frequency in CEU) of *ZNF707* (*p* < 0.01).

Moreover, rs11136002-TT (high frequency in JPT) increased the transcriptional activity of *TNFRSF10B* compared to CC (high frequency in CEU) (*p* < 0.05). This significantly increased transcriptional activity demonstrates that TT, which showed a high frequency in JPT in both rs4873815 and rs11136002, may induce the expression of *ZNF707* or *TNFRSF10B*, respectively, by regulating promoter activity. 

### 3.5. Seven Identified Genes Were Differentially Expressed in Ov-CCA Compared to Normal or HGSOC

Using the gene datasets from our sample cohort (7 normal ovarian tissue samples, 26 HGSOC samples, and 19 Ov-CCA samples), we examined the expression of the seven genes (*APBA3*, *C8orf58*, *KIAA1967*, *NAPRT1*, *RHOBTB2*, *TNFRSF10B*, and *ZNF707*) identified in this study. The clinicopathological features of EOC patients of our cohort are summarized in Table S5.

**Figure 3:**
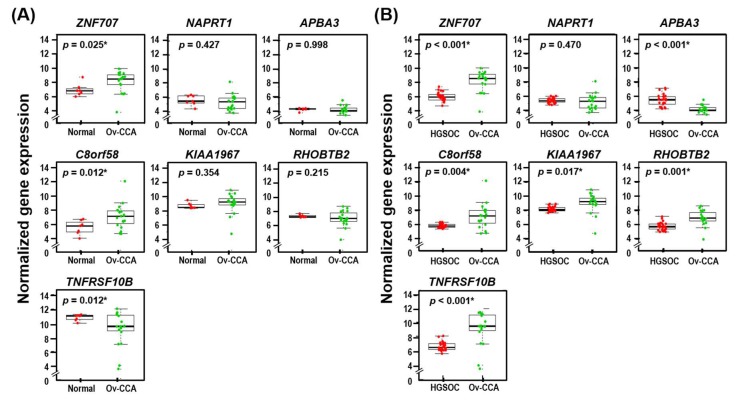
Expression of seven identified genes of our ovarian sample cohorts. (**A**) between normal ovarian tissue samples (*n* = 7) and Ov-CCA (*n* = 19) samples and (**B**) between high-grade serous ovarian cancer (HGSOC; *n* = 26) and Ov-CCA (*n* = 19) samples. *p*-values < 0.05 are flagged with an asterisk (*).

The expression of *ZNF707* and *C8orf58* (*p* = 0.025 and *p* = 0.012, respectively) in Ov-CCA was significantly higher than that of normal ovarian tissues. Meanwhile, the expression of *TNFRSF10B* (*p* = 0.012) was decreased significantly in Ov-CCA compared to normal ovarian tissue (Figure 3A).

Furthermore, we demonstrated that expression of *ZNF707*, *C8orf58*, *KIAA1967*, *RHOBTB2*, and *TNFRSF10B* was significantly increased in Ov-CCA in comparison to HGSOC (*p* < 0.001, *p* = 0.004, *p* = 0.017, *p* = 0.001, and *p* < 0.001, respectively), whereas expression of *APBA3* was significantly lower in Ov-CCA (*p* < 0.001, Figure 3B).

### 3.6. Prognostic Significance of the Identified Genes in Clear Cell Histology

**Figure 4:**
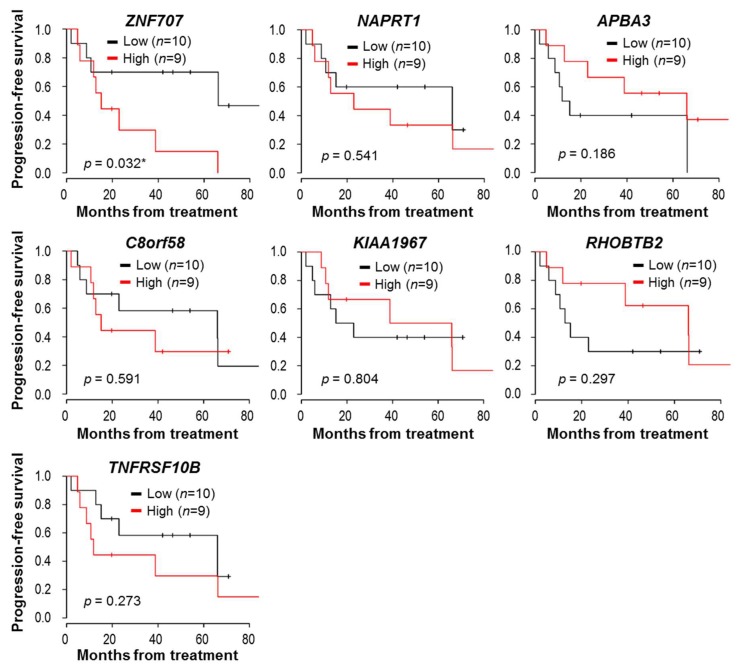
Kaplan–Meier survival plot for seven genes in all stages of Ov-CCA (*n* = 19) samples. Expression values of each gene were dichotomized into high and low expression using the median as a cutoff. Black line: low expression, red line: high expression. *p*-values < 0.05 are flagged with an asterisk (*).

The survival analysis was conducted using the datasets of our Ov-CCA and HGSOC samples. Although the analyzed sample was small, higher *ZNF707* expression was statistically associated with decreased PFS in all stages of Ov-CCA sample analysis (*p* = 0.032) (Figure 4).

In addition, there was a significant association between *C8orf58* and *TNFRSF10B* expression and PFS in the subset analysis with advanced-stage HGSOC (*n* = 26) and advanced-stage Ov-CCA (*n* = 10) samples (*p* = 0.009 and *p* = 0.025, respectively) (Figure S2). Meanwhile, there were no significant correlations between the identified genes and survival outcomes in the overall Ov-CCA and HGSOC dataset analysis (Figure S3).

**Figure 5:**
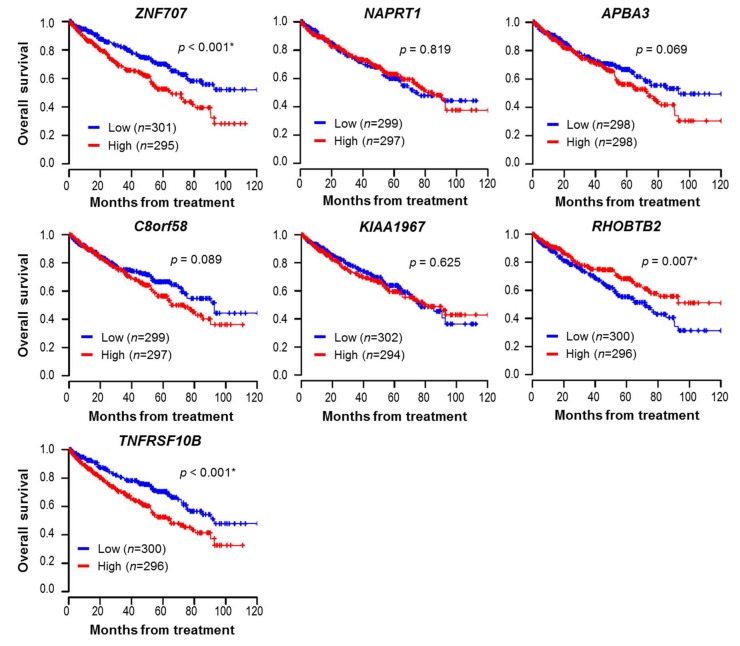
Kaplan–Meier survival plot for seven genes in clear cell renal cell carcinoma (ccRCC) populations (*n* = 596) of The Cancer Genome Atlas (TCGA) datasets. Overall survival analysis was performed using the TCGA-ccRCC dataset. Expression values of each gene were dichotomized into high and low expression using the median as a cutoff. Blue line: low expression, red line: high expression. *p*-values < 0.05 are flagged with an asterisk (*).

Furthermore, to validate the above results in a relatively large cohort regardless of tissue origin, Kaplan–Meier plots of the TCGA-ccRCC datasets (*n* = 596) are presented in Figure 5. A higher expression of *ZNF707* (median survival, 65.1 months vs. not reached; *p* < 0.001) or *TNFRSF10B* (64.9 months vs. 92.8 months, *p* < 0.001) and a lower expression of *RHOBTB2* (71.8 months vs. not reached, *p* = 0.007) were significantly associated with decreased ccRCC patient overall survival. However, there were no significant survival differences for the seven genes in HGSOC (*n* = 265), as shown in Figure S4.

## 4. Discussion

Through multistep *cis*-eQTL analyses using HapMap3 dataset, four SNPs (rs4873815, rs12976454, rs11136002, and rs13259097) and seven associated genes (*APBA3*, *C8orf58*, *KIAA1967*, *NAPRT1*, *RHOBTB2*, *TNFRSF10B*, and *ZNF707*) were identified by differences in disease prevalence of Japanese ancestry. Of the seven genes identified, five (*APBA3*, *KIAA1967*, *RHOBTB2*, *TNFRSF10B*, and *ZNF707*) were reported in 94 previously identified genes, while the remaining two (*C8orf58* and *NAPRT1*) are newly reported genes in Ov-CCA [19].

Multiple in silico and in vitro analyses were conducted to demonstrate the function of the four SNPs and seven associate genes in Ov-CCA. rs4873815-TT, which demonstrated enrichment in the Japanese ancestry compared to European and Chinese ancestries, induced promoter activity on *ZNF707* compared to rs4873815-CC. In addition, rs11136002-TT, which was also enriched in the Japanese ancestry in comparison to the others, induced more promoter activity on the *TNFRSF10B* gene in comparison to rs11136002-CC. In expression analyses of our sample cohorts, *ZNF707*, *C8orf58*, and *TNFRSF10B* were differentially expressed in Ov-CCA compared to normal ovarian tissue or HGSOC. *ZNF707*, *C8orf58*, and *TNFRSF10B* showed significantly higher expression in Ov-CCA compared to HGSOC.

In survival analyses of our Ov-CCA cohort, only high expression of *ZNF707* was associated with poor prognosis in Ov-CCA disease recurrence. To verify that these findings are consistently observed in large cohorts with similar molecular traits, survival analyses of the TCGA-ccRCC and TCGA-HGSOC datasets were conducted. High expression of *ZNF707* and *TNFRSF10B* and low expression of *RHOBTB2* were significantly associated with poor overall survival in the TCGA-ccRCC population, whereas there was no association in the HGSOC population.

These results demonstrate that the four susceptibility loci and seven identified genes, especially rs4873815-TT/*ZNF707*, may be plausible candidates for elucidating Ov-CCA carcinogenesis or prognostication. Moreover, high expression of *ZNF707* may have prognostic power in clear cell histology regardless of tissue origin.

*ZNF707* is one of the genes upregulated in EOC compared to normal human ovarian surface epithelial cells in cDNA microarray analysis (fold: 3.42, *p* < 0.05) [29]. Interestingly, Borghese et al. reported that *ZNF707* is hypermethylated in ovarian endometrioma in comparison to eutopic endometrium (fold: 1.884) [30]. Given that endometriosis increases the risk of Ov-CCA [16], *ZNF707* may play a functional role in tumorigenesis of endometriosis-associated Ov-CCA. Our findings support this rational assumption by deriving the result that *ZNF707* is highly expressed in Ov-CCA and high *ZNF707* expression has negative value in Ov-CCA prognostication. Based on previous research and our study findings, we suggest that *ZNF707* may be involved in Ov-CCA tumorigenesis or prognostication.

Although the six remaining genes (*APBA3*, *C8orf58*, *KIAA1967*, *NAPRT1*, *RHOBTB*, and *TNFRSF10B*) did not show consistent association with survival outcome in EOC and clear cell histology, there have been several reports showing that the remaining genes are correlated with tumorigenesis in other cancers. *TNFRSF10B* is a member of the tumor necrosis factor (TNF) receptor superfamily that is activated by TNF-related apoptosis-inducing ligand (TRAIL), which has been demonstrated to induce selective apoptosis. Several polymorphisms and mutations of the TRAIL and TRAIL receptor genes have been suggested as risk or prognostic factors in lymphoid malignancies as well as breast, colon, liver, lung, and prostate cancer [31,32,33]. Specifically, loss of *TNFRSF10B* expression is a frequent event in hepatocellular carcinoma (HCC) [34] and lung [35] and testicular cancers [36].

*RHOBTB2*, also known as *DBC2*, is an important tumor suppressor gene related to various carcinomas, including those of the breast, bladder, colon, liver, lung, and prostate [37,38,39,40]. Furthermore, patients with *RHOBTB2*-negative breast cancer have been linked to poor prognosis, and *RHOBTB2* silencing is an independent risk factor for breast cancer [40].

In addition, *APBA3* promotes aerobic glycolysis by activating hypoxia-inducible factor 1 (HIF-1) in macrophages; therefore, inhibition of *APBA3* in macrophages contributes to suppression of cancer cell metastasis [41]. *C8orf58* is downregulated in HCC and coexpressed with MEG3 protein, which acts as a suppressor in tumor cells [42]. *KIAA1967* has been reported as one of the fusion partners with *FGFR2* by transcriptome sequencing in lung squamous cell carcinoma of the TCGA database [43]. Lastly, *NAPRT1*, a gene that codes for an NAD-producing enzyme, is amplified and overexpressed in a subset of common types of cancer, including ovarian cancer [44].

As we identified four SNPs and seven associated genes based on the difference of Ov-CCA prevalence by Japanese ancestry, the potential role of seven identified genes in Ov-CCA prognostication or tumorigenesis may be limited to Japanese ancestry. Given that ccRCC has a similar molecular profile to Ov-CCA [28], we performed expression and survival analyses to verify the clinical significance of seven associated genes with the TCGA-ccRCC dataset composed of various ancestries. In addition, further validation was conducted in a minority ancestry with our Korean EOC cohort. Through this multistep analysis using various cohorts, rs4873815-TT/*ZNF707* demonstrated potential prognostic value in Ov-CCA patients. Although further investigation is needed, rs4873815-TT/*ZNF707* may also have a prognostic power in clear cell histology regardless of cancer tissue.

Because the public Ov-CCA dataset was not available, the TCGA-ccRCC dataset was used as an alternative, based on the molecular similarity between ccRCC and Ov-CCA. In addition, our EOC cohort sample size was too small to generalize study findings. Six identified genes, excluding *ZNF707*, did not show significant differences or consistent results in the expression and survival analyses of Ov-CCA. It is possible that these six genes may have nothing to do with Ov-CCA tumorigenesis, that is, the risk locus can be an eQTL for the target gene, but this eQTL–target gene association may be independent of the risk of a specific trait [1]. However, the potential role of the remaining six genes should be comprehensively verified in the near future with relatively large and diverse Ov-CCA datasets.

In conclusion, we identified four SNPs and their seven associated genes in Ov-CCA through *cis*-eQTL analysis based on the differences in allelic frequencies of a Japanese ancestry. rs4873815-TT/*ZNF707* may have clinical significance in the prognosis and tumorigenesis of Ov-CCA, which may be more relevant with clear cell histology.

This study suggests that *cis*-eQTL analysis using publicly available datasets may facilitate the discovery of novel genetic alterations in Ov-CCA and provide the basis for understanding its tumorigenesis. Although further investigation is needed to validate the potential role of the remaining six genes in Ov-CCA tumorigenesis, our findings support the existing evidence that genetic ancestry can be an attractive clue to complete the genomic landscape of complex diseases, such as cancer.

## Figures and Tables

**Table 1:** Four SNPs and seven associated genes of ovarian clear cell adenocarcinoma (Ov-CCA).

SNP	Chr.	Cytoband	SNP Position	Transcripts Associated in *cis*	Gene Start	Gene End	*p*-Value
rs4873815	8	8q24.3	144796206	*ZNF707*	144824516	144849514	8.92e−4
		8q24.3		*NAPRT1*	144728101	144731656	4.41e−4
rs12976454	19	19p13.3	3495971	*APBA3*	3701771	3712673	3.40e−4
rs11136002	8	8p21.3	22273027	*C8orf58*	22513067	22517605	5.53e−4
		8p21.3		*KIAA1967*	22518202	22533920	9.19e−4
		8p21.3		*RHOBTB2*	22913059	22933655	3.12e−4
				*TNFRSF10B*	22933598	22982637	4.03e−4
rs13259097	8	8p21.3	22189689	*RHOBTB2*	22913059	22933655	3.87e−4

SNP, Single-nucleotide polymorphism; Chr., chromosome.

## Supplementary Materials

The following are available online at https://www.mdpi.com/2077-0383/9/4/1137/s1. Figure S1: The results table with Ensembl Variant Effect Predictor (VEP) analysis of four susceptibility loci. Figure S2: Kaplan–Meier survival plot for seven genes in HGSOC (*n* = 26) and advanced-stage Ov-CCA (*n* = 10) samples. Figure S3: Kaplan–Meier survival plot for seven genes in all epithelial ovarian cancer samples (*n* = 45). Figure S4: Kaplan–Meier survival plot for seven genes in HGSOC populations (*n* = 265) of TCGA datasets. Table S1: Details of the HapMap3 dataset (release 3). Table S2: Details of the 935 identified SNPs. Table S3: Primers and sequencing result in Promoter assays. Table S4: Four SNPs with different allelic frequency between Japanese and Chinese populations. Table S5: Clinicopathologic characteristics of patients in our sample cohorts.
