# Supplementary material for: Identification of Candidate Genes Associated with Susceptibility to Ovarian Clear Cell Adenocarcinoma Using cis-eQTL Analysis
Source: J Clin Med. 2020 Apr 16;9(4):1137. doi: 10.3390/jcm9041137 (PMC7231141; doi:10.3390/jcm9041137)
Supplement: Supplementary file 1 [file jcm-09-01137-s001.zip › jcm-764339-supple-for conversion/jcm-764339-supple.docx]

Supplementary Materials: Identification of Candidate Genes Associated with Susceptibility to Ovarian Clear Cell Adenocarcinoma Using *Cis*-eQTL Analysis

Jihye Kim, Joon-Yong Chung, Jae Ryoung Hwang, Yoo-Young Lee, Tae-Joong Kim, Jeong-Won Lee, Byoung-Gie Kim, Duk-Soo Bae, Chel Hun Choi and Stephen M. Hewitt


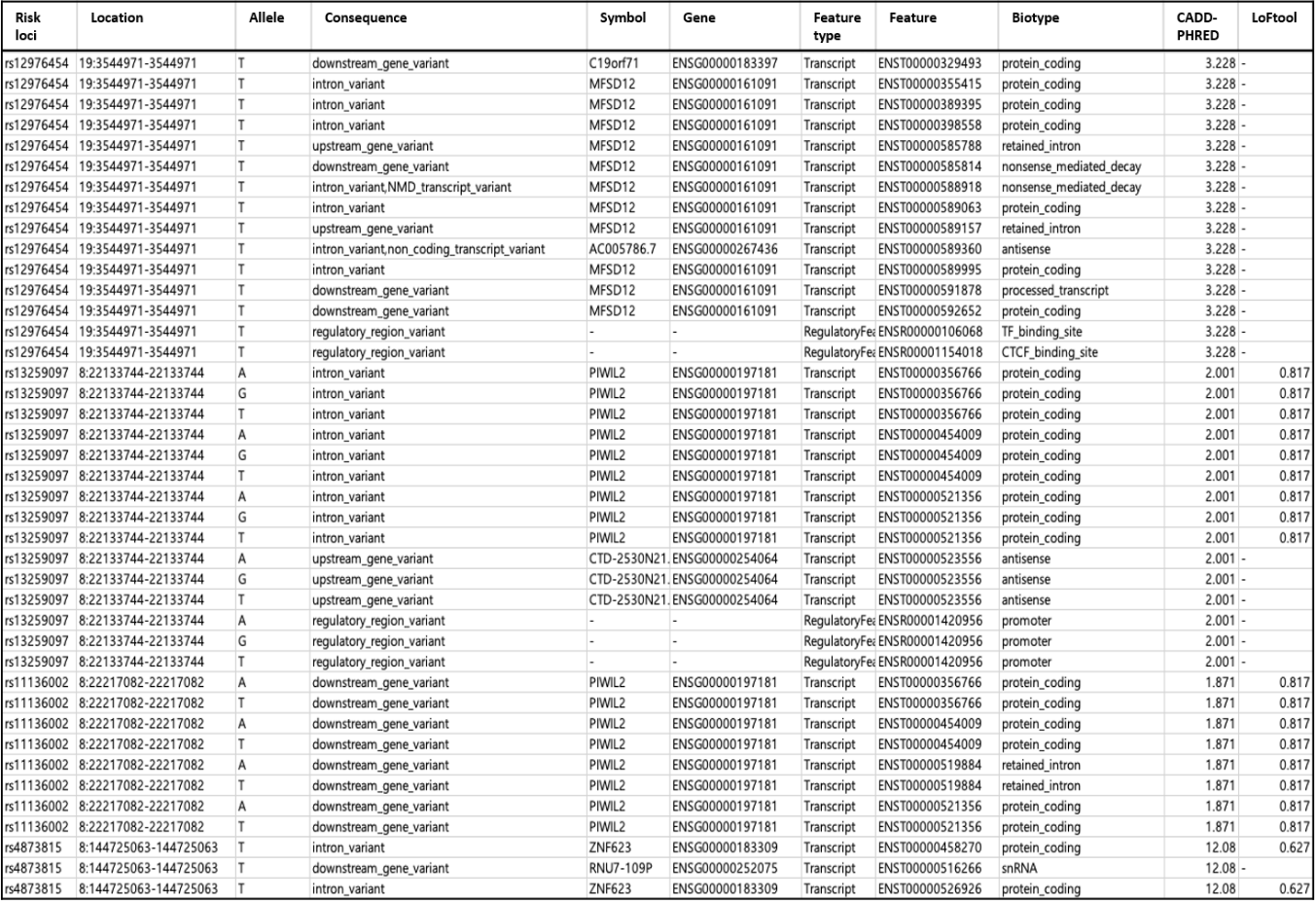


**Figure S1.** The results table with Ensemble Variant Effect Predictor (VEP) analysis of four susceptibility loci.


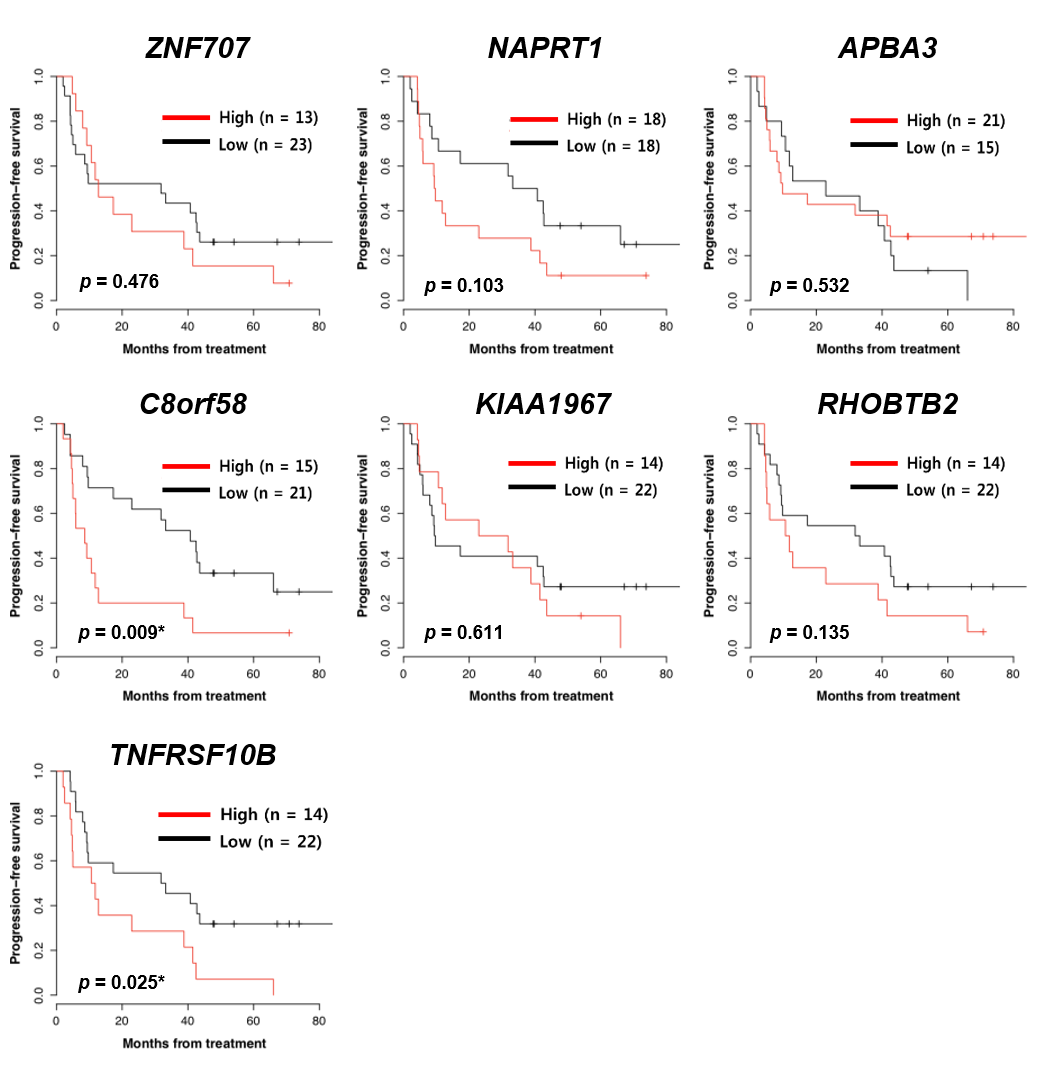


**Figure S2.** Kaplan-Meier survival plot for seven genes in HGSOC (*n* = 26) and advanced stage Ov-CCA (*n* = 10) samples. Expression values of each gene were dichotomized into high and low expression using the median as a cut-off. Black line: low expression and red line: high expression. *p-*values < 0.05 are flagged with an asterisk (*).


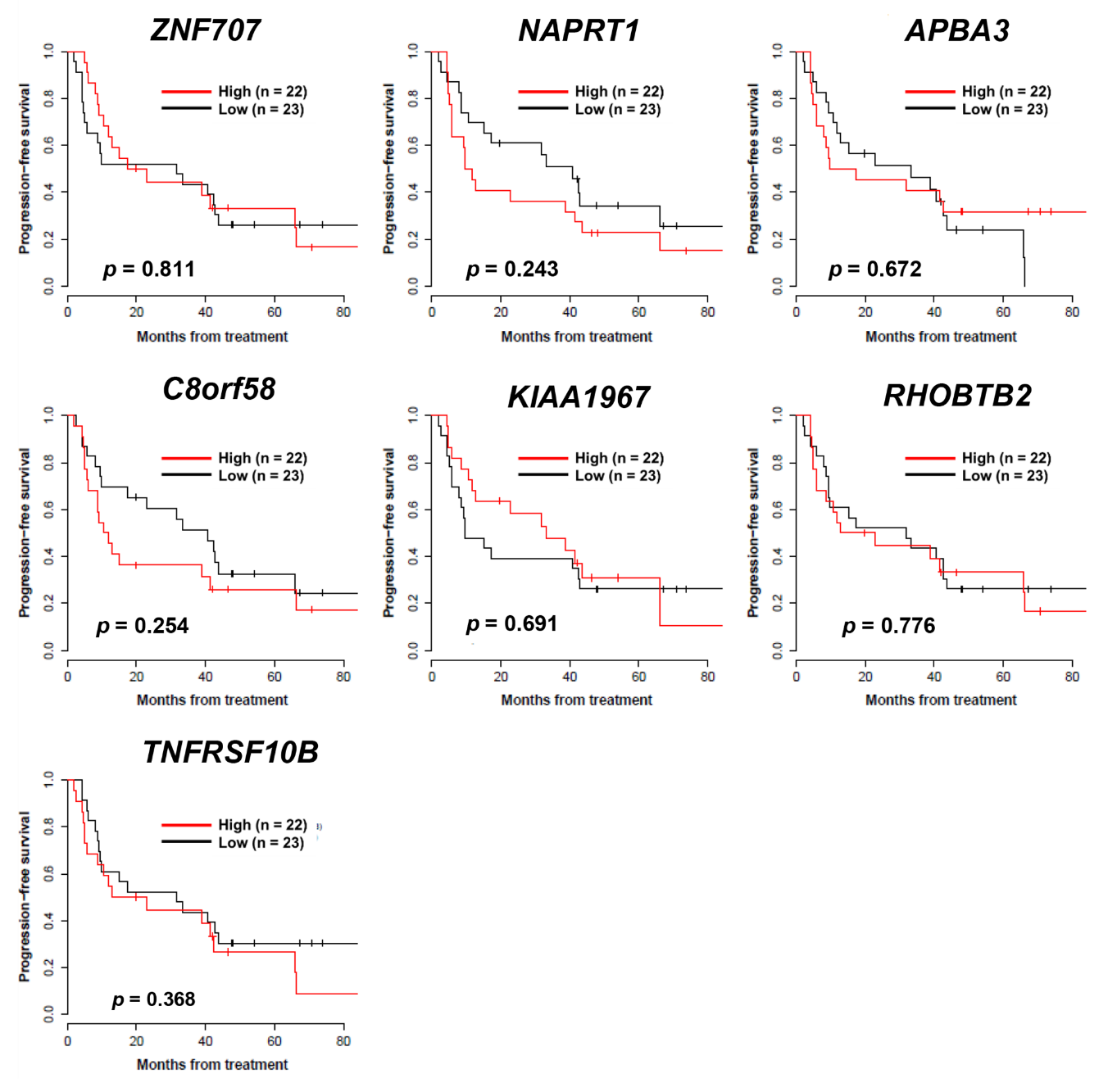


**Figure S3.** Kaplan-Meier survival plot for seven genes in all epithelial ovarian cancer samples (*n* = 45). Expression values of each gene were dichotomized into high and low expression using the median as a cut-off. Black line: low expression and red line: high expression. *p*-values < 0.05 are flagged with an asterisk (*).


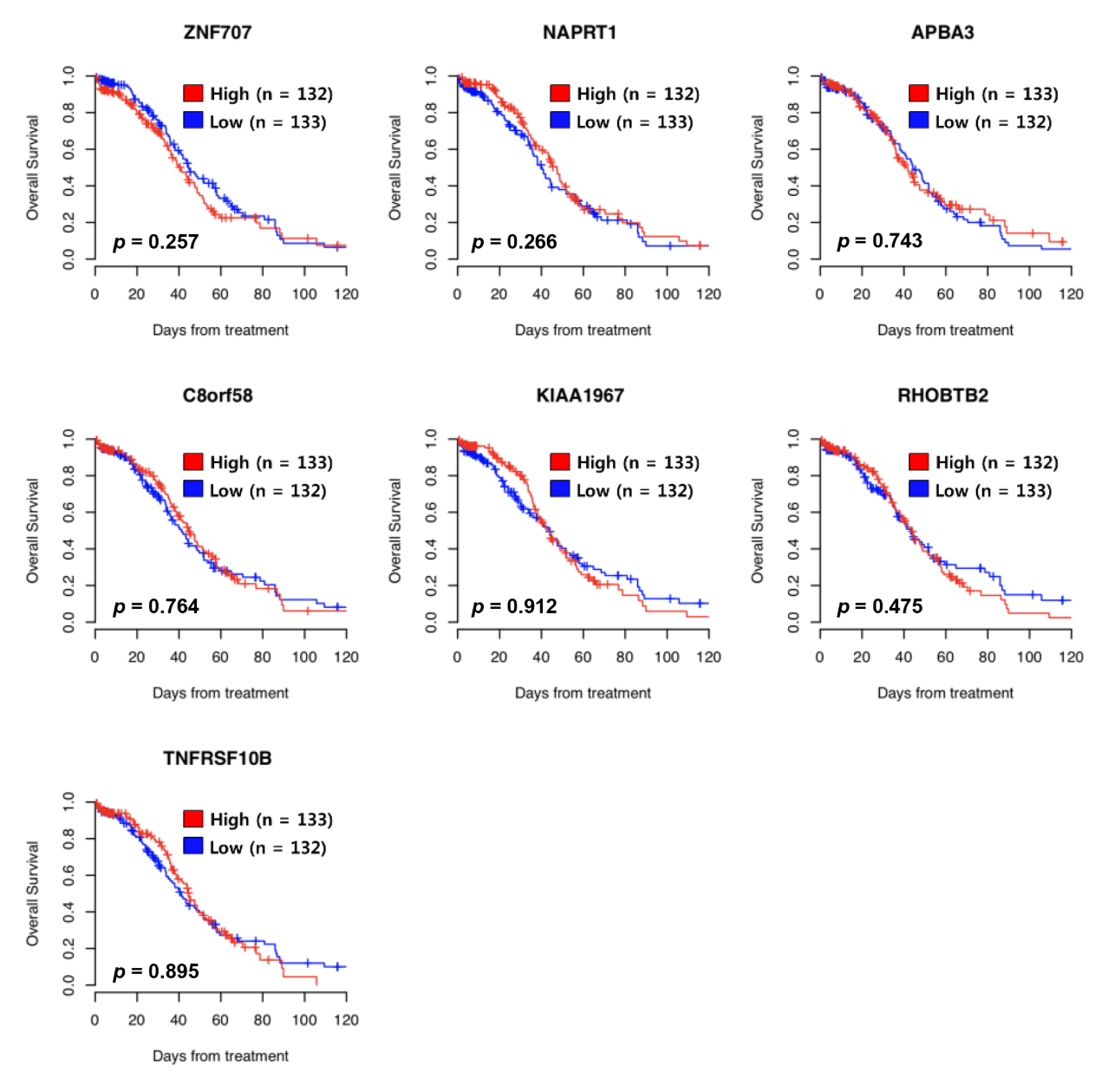


**Figure S4**. Kaplan-Meier survival plot for seven genes in HGSOC populations (*n* = 265) of TCGA datasets. Overall survival analysis was performed using the TCGA-HGSOC dataset. Expression values of each gene were dichotomized into high and low expression using the median as a cut-off. Blue line: low expression and red line: high expression. *p-*values < 0.05 are flagged with an asterisk (*).

**Table S1.** Details of the HapMap3 dataset (release 3)

| **Population** | **Description** | **No. samples** | **No. SNPs QC** | **No. identified SNPs** |
| --- | --- | --- | --- | --- |
| CEU | Utah residents with Northern and Western European ancestry from the CEPH collection | 165 | 1,623,122 | 200 |
| CHB | Han Chinese in Beijing, China | 137 | 1,626,122 | 85 |
| GIH | Gujarati Indians in Houston, Texas | 101 | 1,630,857 | 197 |
| JPT | Japanese in Tokyo, Japan | 113 | 1,634,041 | 68 |
| LWK | Luhya in Webuye, Kenya | 110 | 1,625,159 | 106 |
| MXL | Mexican ancestry in Los Angeles, California | 86 | 1,604,948 | 68 |
| MKK | Maasai in Kinyawa, Kenya | 184 | 1,611,733 | 124 |
| YRI | Yoruba in Ibadan, Nigeria | 203 | 1,625,669 | 87 |
| Consensus |  | 1099 | 1,481,135 | 935 |

No., Numbers; SNP, single nucleotide polymorphism; QC, quality control. 23238 monomorphic SNPs removed from consensus.

**Table S3.** Primers and sequencing result in Promoter assays.

1. Primers used for sequencing

| **Inner primers for TNFRSF10B promoter** | **Sequence** |
| --- | --- |
| Seq-TNFRSF10B-1 (sense)  Seq-TNFRSF10B-2 (sense)  Seq-TNFRSF10B-3 (antisense) | 5’-TTTGTGAATGCACCAATTGAC-3’  5’-AGACACGCCGCCTTTAAGAAC-3’  5’-CTAGATACACAGTGCCAATTG-3’ |
| **Inner primers for ZNF707 promoter** | **Sequence** |
| Seq-ZNF707-1 (sense)  Seq-ZNF707-2 (sense)  Seq-ZNF707-3 (sense)  Seq-ZNF707-4 (antisense) | 5’-GGCTATACTTTGGATGCTTTC-3’  5’-AGTGTCTGGGAAGTCACC-3’  5’-TGGACCCTACACCCTTGA-3’  5’-GTATAGCCAAGAAATATA-3’ |

2. Sequence results

1). CC in rs11136002

CACATCTGATCAAGGATTGTTATCCAAAATATACAAAGAACTCTTTAAACTTAATAAAGAAACTAGTCAGTTTTTTTAAAAATCGGCAATAGACCTGTACAGACATCGCCAAAGATGCACAGATGGTAAATAAACGTATGAAAAGATGCTCCACATTATATCTCCTTAGGGAACCACAAATTAAAACAAGGCACCCATTCCATACCTGGTAGAATAGCCAAAATCCACAACACTTAACCACGCCATATGCTGGTGAGGTTGCAGAGCTGCAGGAACTGGTACAGCCACTTGAGAAGAGAGTTCTTAATAAAATTAAACAGGATTACAAAACCACATACATAATCTTATCATATGGAGCAGCAGTCATACTCCTTGGTGTTTACCCAAAGGGGATGAAAACTCATGTCCACACAAAAGCCTGCACACGGACATTCATAGCAGCTGTATTCATCGTTTCC

* SNP region was highlighted in green color.

2). TT in rs11136002

CACATCTGATCAAGGATTGTTATCCAAAATATACAAAGAACTCTTTAAACTTAATAAAGAAACTAGTCAGTTTTTTTTAAAAATCGGCAATAGACCTGTACAGACATCGCCAAAGATGCACAGATGGTAAATAAACGTATGAAAAGATGCTCCACATTATATCTCCTTAGGGAACCACAAATTAAAACAAGGCACCCATTCCATACCTGGTAGAATAGCCAAAATCCACAACACTTAACCATGCCATATGCTGGTGAGGTTGCAGAGCTGCAGGAACTGGTACAGCCACTTGAGAAGAGAGTTCTTAATAAAATTAAACAGGATTACAAAACCACATACATAATCTTATCATATGGAGCAGCAGTCATACTCCTTGGTGTTTACCCAAAGGGGATGAAAACTCATGTCCACACAAAAGCCTGCACACGGACATTCATAGCAGCTGTATTCATCGTTTCC

* SNP region was highlighted in green color.

3). CC in rs4873815

CTCAATATTGTTATATCCAGGGAAGGGGGAGGCTGGAGGAAAGGGAGAGATGGGGAACAGCCGGTTGGTGGTGGATGTAGGAAACACACATTGATAATTCAGCTTCTTATATGGGTGCGGTTCATGGTGCCCCAAACAGTGACAGTAGTTTTAGATTCCTGTTACCTGCCGGGCACCTGGCATGGGTGTGGAGGGCAGAGTTCTGAGGTGGTCTCTTAGATTCCTGGCCCCTAATGTACACACCCCTTCTTCCAGTTACTCATTCAGACACTCATCTGGTACTGTCATGAAGAGACTTGCAGATGTCGGGAGGTCCCAGGTCAGTGAGGTGAGAAGGGAGGTCGTCTGGGAGAGCCTGACCTAATCACACAGACCCTGGGAAGCAGAGGTTCCTCCATCTGGTTGGAGAACAGAGGCTGAGATTAGATGCGTGAGAAAAATGTGATGTG

* SNP region was highlighted in green color.

4). TT in rs4873815

CTCAATATTGTTATATCCAGGGAAGGGGGAGGCTGGAGGAAAGGGAGAGATGGGGAACAGCCGGTTGGTGGTGGATGTAGGAAACACACATTGATAATTCAGCTTCTTATATGGGTGCGGTTCATGGTGCCCCAAACAGTGACAGTAGTTTTAGATTCCTGTTACCTGCCGGGCACCTGGCATGGGTGTGGAGGGCAGAGTTCTGAGGTGGTCTCTTAGATTCCTGGCTCCTAATGTACACACCCCTTCTTCCAGTTACTCATTCAGACACTCATCTGGTACTGTCATGAAGAGACTTGCAGATGTCGGGAGGTCCCAGGTCAGTGAGGTGAGAAGGGAGGTCGTCTGGGAGAGCCTGACCTAATCACACAGACCCTGGGAAGCAGAGGTTCCTCCATCTGGTTGGAGAACAGAGGCTGAGATTAGATGCGTGAGAAAAATGTGATGTG

* SNP region was highlighted in green color.

5). TNFRSF10B promoter

GCACCCGGGCCAGCGGCTGCGGAGGGTGTACTGGGTCCCCCAGCAGTGCCGGCCCACTGGCGCTGCGCTTGATTTCTCGCCGGGCCTTAGCTGCCTTCCCGACGGGCAGGGCTCGGGACCGCCATGCCTGAGCCTCTCACCCCCTCCGTGGGCTCCTGTGCAGCCGGAGCCTCCCCGACGAGCACCTCCCCCTGCTCCAGGGCGCCCAGTCCCATCGACCACCCAAGGGCTGAGGAGTGCGGGCGCACGGCGCGGGAGCACGGCGCGGGACTGGCAGGCAGCTCCACCTGCAACCCCGGTGCGGGATCCACTGGGTGACGACACCTGGGCTCCTGAGTCTGGTGGGGACGTGGAGAACCTTTATGTTTAGCTAAGGGATTGTAAATACACCAATTGGCACTGTGTATCTAGCTCAAGTTTTGTAAACACACCAATCAGCACCGTGTGTCTAGCTCAGGGTTTGTGAATGCACCAATTGACACTCTGTATCTAGCTAGTCTGGTGGGGCCTTGGAGAACCTTTGTGTCCACACTCTGTATCTAGCTAATCTGGTGGGGAAGTGGAGAACATTTGTGTCTAGCTCAGGGATTGTAAACCACCAATCAGCGCCCTGTCAAAACAGACCACTCCGCTCTACCAATCAGCAGGGTGTGGGTGGGGCCAGATAAGAGAATAAAAGCAGGCTGCCAGAGCCAGCAGTGGTAACACTGTGGAAGCTTCCTTCTTTCCCTCTGCAATAAATCTTGCTACTGCTCACTCTTTGGGTCCACACTGCCTTTATGAGCTGTAACACTCACCTTGAAGGTCTGCAGCTTCACTCTTGAAGCCAGCGAGACCACGAGCCCACCGGGAGGAAAGAACAACTCCAGACCCACTGCCTTAAGAGCTGTAACACTCACTGGGAAGGTCTGCAGCTTCACTCCTGAGCCAGTGAGACCACGAACCCACCAGAAGGAAGAAACTCCGAACACATCCGAACATCAGAAGGAACAAACTCCAGACACGCCGCCTTTAAGAACTGTAACACTCACCGCGAGGGTCCGAGGCTTCATTCTTGAAGGCAGTGAGACCAAGAACCCACCAATTCCGGACACAGTACCATGAAGGAATGAAAATACATAACAATGTGATGTATCATGTTTTATTTCCTAGACTAGTGACAAATGAAAGCTAAGTGTAGCAAGGGTGCAGGGACACAGGCACATTTGTGGACTAGGTGTGAGTGTAAGCTGGGTTCGATGGTCTTTTGGCCAACATAGTGAACCCCTGTGTCTACTAAAAATACAAAAATTAGCCAGGCGTGGTGGTGCAGGCCTGTAGTCCCAGCTACATGGGAGGCTGAGGTGGGAGTATCGCTTGAACCTGGGAGACGGAAGTTGCAGTGAGCCGGGATCACACCACCGCTCACCAATCTGAGCCACAGAGAGACTGTCTCAAAAAATAAACCACAAGGAAGGGAGGTAGGGGGAGGGGGAGGGAGGGAGGAAAGAGAAAGAGAGAAAGGAAGGAAAGAGAAAGCAGGAAGGACGGAAAGAAGACGAAAGAACGAAAGAAAACGAAAGAAAAAAGGAAAGAAGAGAGAAGGAGAGAACAGAAGGGGCAGGTGCCCCTGGGAAGGGGAGAAGATCAAGACGCGCCTGGAAAGCGGACTCTGAACCTCAAGACCCTGTTCACAGCCAAGCGCGCGACCCCGGGAGGCGTCAACTCCCCAAGTGCCTCCCTCAACTCATTTCCCCCAAGTTTCGGTGCCTGTCCTGGCGCGGACAGGACCCAGAAACAAACCACAGCCCGGGGCGCAGCCGCCAGGGCGAAGGTTAGTTCCGGTCCCTTCCCCTCCCCTCCCCACTTGGACGCGCTTGCGGAGGATTGCGTTGACGAGACTCTTATTTATTGTCACCAACCTGTGGTGGAATTTGCAGTTGCACATTGGATCTGATTCGCCCCGCCCCGAATGACGCC

6). ZNF707 promoter

GTGAGGACTAAACTGATCTTTTTCTCTCTTGCTCAAGTTCTTATCTAAAGGGCCTGGGGAGTCTGCCCTACAAACCATAAAATGTCATCAGATAGGTTTTATTTAACCCTATATAATTGGCTTAGTTTCCAACCTGACTCTGGCATAACGTCACATGACAGATAAAGGAAATCAAAATATTTTACTCTTTTTTTTTTTTTTTTTGAGACGGAGTCTCACTCTGTCGCCCAGGCTGGAATGGAGTGGCGCGATCTCGGCTCACTGCAAGCTCCGCCTCTGGGTTTCACACCGTTCTCCTGCCTCAGCCTCCCGAGTAGCTGGGACTACAGGCGCCCGCCACCATGCCCGGCTAATTTGTTGTATTTTTAGTAGAGACGGGTTTCACCATGTTAGCCAGGATGGTCTCGATCTCCTGACCTCGTGATCCACCCGCCTCGGCCTCCCAAAGTGCTGAGATTACAGGCGGGAGCCACCGTGCCCGGCCTATTTTACTTTTAAATATATTTCTTGGCTATACTTTGGATGCTTTCAGGTCATAGGTGGATTCAAAGATTTTCTGATTGGCAATTGGTTGAATAAGTTAAGTGATTACCTAAGACCTGGAATCTATAGAAAGGCGTGTCTAGGTTTGAACAGTTGGCCGCCCTTGTGTGGGTGGAGTATTACATAGGTGCCGAGGCAAGAGACTGAAGCCACAAACTGTTTCAGTATAATAAAGAAAATAGTTAGAATAAGAATAGTCATAATACAAATTAGATATAGAGATGACCATGAACAATTATCAATCATTATTATAAACATTATTAATCATTAGCTTTTACCATTACTCTTTGTTGCATTACTAATATAACCTAGGAATAACCGGCGGATATAGGGTCGGGTGCTGAAGGGACATGGTGAGAAGTGACCTAGAAGGCAAGAGGTGAGCCTTCTGTCACTCCCACATCAGGGCTGCTTGAGGGCTCCTTGGTCAAGCCCTAACGCCAGTGTCTGGGAAGTCACCCGTTGCTTAGTAGACTGCGAAAGGGAGTCTCCTTTCCTTGGAGGAGTCAGGGAACACTCTGCTCCACCAGCTTCTTGTGGGAGGTTGGATATTACCCAGGCCTGGCCGCAGTCATCCGGAGGCCTAAACCCCTCCCTGTGGTGCTTCAATGTTCACGCTCCTTGTCCACTTTCGTGTTCCTCCTGTACTCCTGGTTCCTCTTTGAAGTTCGTAGTCGATAGCGGTAGAAGAAATAGTGAAAGTCTTAAAGTCTTTGATTAATGCTAACTTATGCTGCCTTCTCTCTCTGCTTCCGCTACCTAAGAGGGAAGGGCCCCCTGTCCTGTAATCATGTGACTTGCTTCACCTTGTCAATCACTTAGAAGATTCACCCTCCTTACCCTGCCCCCCCTTGTCTTGTATGCAATAAATATCAGCGCGCCCAGCCGTTTGGGGCCACTACCGGTCTCTGCGTCTTGATGGTAGTGGTCGCCCGGGCCCAGCTGTTTTCTCTTTATCTCTTTGTCTTGTGTCTTTATTTATTACAATCTCTCATCTCCGCACACGGGGACAACACCCGCTTAGCCCCGCAGGGCTGGACCCTACACCCTTGATTGGCTGGAACTGGGTGACTGGCAGGAGAGCAAGTGACAGTCTGTTCACACCTCCAGTTAGGTTACAGTTCACTTTGTAGGATAAACCTTTAGGCCGACTTTAGGTTATGAGGGCCGGGATCGGTGGTTCACGCCTGTAACCCCAGCACTTTGGGAGGCCAAGTCAGGCGGATCACCTGAGGTCAGGAGTTCAAAACCAGCCTGACCAACATGATGAAACCCCCGTCTCTACTAAAATTACAAAAAAATCAGCCGGGCGTCGTGGCGCTTGGCTGTGACCGGGGCGGGGCCTGCCGGCTGCGGCGGAACCACAAGCGGTGCGGGGCGAGGCGGGCGGCCTGGACGGCCTGGAAGGCCAGCGCGCACCACCGAGACGTGGGCTCCTAGAGGGGCCGGAAGCTT

**Table S4.** Four SNPs with different allelic frequency between Japanese and Chinese populations.

|  | ***Log* FC** | **Ave. Exp.** | **t** | ***p*-value** | **Adj. *p*-value** | **B value** |
| --- | --- | --- | --- | --- | --- | --- |
| rs4873815 | -2 | 1.961538 | -50.6085 | 5.78e-44 | 4.38e-41 | 77.68311 |
| rs12976454 | -2 | 1.928571 | -7.20963 | 3.44e-11 | 1.30e-08 | 15.04775 |
| rs11136002 | -0.66667 | 1.981818 | -5.13524 | 1.27e-06 | 0.000321 | 4.611563 |
| rs13259097 | -0.66667 | 1.977528 | -4.6038 | 1.42e-05 | 0.002693 | 2.312036 |

Log FC, Log Fold Change; Ave.Exp, Average expression; Adj. p-value, Adjusted p-value.

**Table S5.** Clinicopathologic characteristics of patients in our sample cohorts.

|  | **HGSOC (*n* = 26)** | **Ov-CCA (*n* = 19)** | ***p-*value** |
| --- | --- | --- | --- |
| Age | 52.1 ± 10.5 | 52.2 ± 8.7 | 0.989 |
| Grade |  |  |  |
| 1 | 1 (3.8%) | 0 (0%) | 0.361 |
| 2 | 5 (19.2%) | 5 (38.5%) |  |
| 3 | 20 (76.9%) | 8 (61.5%) |  |
| Stage |  |  | <0.001* |
| I/II | 0 (0.0%) | 9 (47.4%) |  |
| III/IV | 26 (100.0%) | 10 (52.6%) |  |
| Optimality |  |  |  |
| Optimal | 12 (46.2%) | 18 (94.7%) | 0.002* |
| Suboptimal | 14 (53.8%) | 1 (5.3%) |  |
| Chemosensitivity |  |  | 0.351 |
| Refractory | 8 (30.8%) | 4 (21.1%) |  |
| Resistant | 4 (15.4%) | 1 (5.3%) |  |
| Sensitive | 14 (53.8%) | 14 (73.7%) |  |

HGSOC, high grade serous ovarian carcinoma; Ov-CCA, ovarian clear cell adenocarcinoma. p-values < 0.05 are flagged with asterisk (*).
