# Supplementary material for: Identification of Candidate Genes Associated with Susceptibility to Ovarian Clear Cell Adenocarcinoma Using cis-eQTL Analysis
Source: J Clin Med. 2020 Apr 16;9(4):1137. doi: 10.3390/jcm9041137 (PMC7231141; doi:10.3390/jcm9041137)
Supplement: Supplementary file 1 [file jcm-09-01137-s001.zip › jcm-764339-supple-for conversion/jcm-764339-table s2.docx]

Supplementary Materials: Identification of Candidate Genes Associated with Susceptibility to Ovarian Clear Cell Adenocarcinoma Using *Cis*-eQTL Analysis

Jihye Kim, Joon-Yong Chung, Jae Ryoung Hwang, Yoo-Young Lee, Tae-Joong Kim, Jeong-Won Lee, Byoung-Gie Kim, Duk-Soo Bae, Chel Hun Choi and Stephen M. Hewitt

**Table S2.** Details of the identified 935 SNPs.

| No | RACE | SNP ID | Probe Id | Ensembl Gene | *Gene Symbol* | Chr | SNP Position | Gene Position | Distance | rho | *p*-value |
| --- | --- | --- | --- | --- | --- | --- | --- | --- | --- | --- | --- |
| 1 | MKK | rs10046718 | ILMN_1699473 | ENSG00000158941 | *KIAA1967* | 8 | 21653378 | 22518202 | 864824 | −0.283 | 8.24e−04 |
| 2 | JPT | rs1008076 | ILMN_1673069 | ENSG00000142002 | *DPP9* | 19 | 3919605 | 4674875 | 755270 | −0.409 | 1.36e−04 |
| 3 | JPT | rs1008128 | ILMN_1673069 | ENSG00000142002 | *DPP9* | 19 | 3917520 | 4674875 | 757355 | −0.409 | 1.36e−04 |
| 4 | GIH | rs10086541 | ILMN_1727618 | ENSG00000156170 | *C8orf38* | 8 | 96103126 | 95977217 | 125909 | 0.4 | 1.96e−04 |
| 5 | GIH | rs10087402 | ILMN_1727618 | ENSG00000156170 | *C8orf38* | 8 | 96168842 | 95977217 | 191625 | 0.412 | 1.21e−04 |
| 6 | YRI | rs10088001 | ILMN_1714364 | ENSG00000169398 | *PTK2* | 8 | 141907542 | 142080514 | 172972 | 0.321 | 7.00e−04 |
| 7 | MEX | rs10088992 | ILMN_1769779 | ENSG00000184489 | *PTP4A3* | 8 | 142798611 | 142501189 | 297422 | 0.486 | 8.31e−04 |
| 8 | GIH | rs10089123 | ILMN_1727618 | ENSG00000156170 | *C8orf38* | 8 | 96166789 | 95977217 | 189572 | 0.412 | 1.21e−04 |
| 9 | GIH | rs10089336 | ILMN_1741841 | ENSG00000164944 | *KIAA1429* | 8 | 96439422 | 95634864 | 804558 | 0.406 | 1.52e−04 |
| 10 | YRI | rs10089769 | ILMN_1811433 | ENSG00000161016 | *RPL8* | 8 | 146241933 | 145988609 | 253324 | 0.313 | 9.58e−04 |
| 11 | MEX | rs10090968 | ILMN_1717420 | ENSG00000104343 | *UBE2W* | 8 | 74994516 | 74923937 | 70579 | −0.553 | 9.76e−05 |
| 12 | GIH | rs10092003 | ILMN_1727618 | ENSG00000156170 | *C8orf38* | 8 | 96105598 | 95977217 | 128381 | −0.4 | 1.96e−04 |
| 13 | CHB | rs10092564 | ILMN_1666022 | ENSG00000173530 | *TNFRSF10D* | 8 | 22942175 | 23077485 | 135310 | 0.38 | 5.14e−04 |
| 14 | GIH | rs10093594 | ILMN_1727618 | ENSG00000156170 | *C8orf38* | 8 | 96102412 | 95977217 | 125195 | −0.4 | 1.96e−04 |
| 15 | YRI | rs10094361 | ILMN_1714364 | ENSG00000169398 | *PTK2* | 8 | 141844240 | 142080514 | 236274 | 0.313 | 9.55e−04 |
| 16 | JPT | rs10094655 | ILMN_1725683 | ENSG00000172728 | *FUT10* | 8 | 32513689 | 33450206 | 936517 | −0.365 | 7.58e−04 |
| 17 | GIH | rs10097930 | ILMN_1727618 | ENSG00000156170 | *C8orf38* | 8 | 96094911 | 95977217 | 117694 | −0.4 | 1.96e−04 |
| 18 | CEU | rs10097936 | ILMN_1725683 | ENSG00000172728 | *FUT10* | 8 | 33450112 | 33450206 | 94 | 0.532 | 2.57e−09 |
| 19 | YRI | rs10098159 | ILMN_1768273 | ENSG00000104356 | *POP1* | 8 | 99078367 | 99199244 | 120877 | 0.345 | 2.52e−04 |
| 20 | GIH | rs10098453 | ILMN_1727618 | ENSG00000156170 | *C8orf38* | 8 | 96098932 | 95977217 | 121715 | 0.4 | 1.96e−04 |
| 21 | GIH | rs10098971 | ILMN_1690546 | ENSG00000120910 | *PPP3CC* | 8 | 21364794 | 22354541 | 989747 | −0.415 | 1.06e−04 |
| 22 | GIH | rs10099630 | ILMN_1741841 | ENSG00000164944 | *KIAA1429* | 8 | 96447777 | 95634864 | 812913 | −0.388 | 3.14e−04 |
| 23 | MKK | rs10099996 | ILMN_1659651 | ENSG00000184428 | *TOP1MT* | 8 | 144467771 | 144488425 | 20654 | −0.319 | 1.38e−04 |
| 24 | CEU | rs10100607 | ILMN_1684694 | ENSG00000029534 | *ANK1* | 8 | 41004601 | 41873155 | 868554 | −0.313 | 9.29e−04 |
| 25 | CEU | rs10103002 | ILMN_1684694 | ENSG00000029534 | *ANK1* | 8 | 41072396 | 41873155 | 800759 | 0.343 | 2.83e−04 |
| 26 | GIH | rs10103250 | ILMN_1727142 | ENSG00000104365 | *IKBKB* | 8 | 42071038 | 42247986 | 176948 | 0.357 | 9.84e−04 |
| 27 | MEX | rs10105699 | ILMN_1692158 | ENSG00000083168 | *MYST3* | 8 | 42759051 | 42028635 | 730416 | 0.489 | 6.59e−04 |
| 28 | JPT | rs10105842 | ILMN_1712517 | ENSG00000185730 | *ZNF696* | 8 | 143668617 | 144444971 | 776354 | 0.366 | 7.83e−04 |
| 29 | MKK | rs10107033 | ILMN_1714081 | ENSG00000008853 | *RHOBTB2* | 8 | 22638374 | 22913059 | 274685 | 0.296 | 4.19e−04 |
| 30 | CEU | rs10108150 | ILMN_1811433 | ENSG00000161016 | *RPL8* | 8 | 145658283 | 145988609 | 330326 | −0.369 | 7.84e−05 |
| 31 | CEU | rs10108836 | ILMN_1811433 | ENSG00000161016 | *RPL8* | 8 | 145807035 | 145988609 | 181574 | 0.357 | 1.47e−04 |
| 32 | CHB | rs10109430 | ILMN_1813635 | ENSG00000164944 | *KIAA1429* | 8 | 95801942 | 95634864 | 167078 | 0.391 | 3.70e−04 |
| 33 | CHB | rs10109512 | ILMN_1804789 | ENSG00000158941 | *KIAA1967* | 8 | 23177004 | 22518202 | 658802 | −0.409 | 1.66e−04 |
| 34 | GIH | rs10111154 | ILMN_1727618 | ENSG00000156170 | *C8orf38* | 8 | 96110063 | 95977217 | 132846 | −0.4 | 1.96e−04 |
| 35 | YRI | rs10112548 | ILMN_1740752 | ENSG00000158669 | *AGPAT6* | 8 | 41988078 | 41554876 | 433202 | 0.325 | 6.08e−04 |
| 36 | JPT | rs10112699 | ILMN_1786345 | ENSG00000158941 | *KIAA1967* | 8 | 22621149 | 22518202 | 102947 | 0.371 | 6.10e−04 |
| 37 | YRI | rs10113248 | ILMN_1740752 | ENSG00000158669 | *AGPAT6* | 8 | 41987963 | 41554876 | 433087 | 0.332 | 4.44e−04 |
| 38 | GIH | rs10113699 | ILMN_1725683 | ENSG00000172728 | *FUT10* | 8 | 33389173 | 33450206 | 61033 | −0.37 | 7.38e−04 |
| 39 | MKK | rs10155 | ILMN_1686610 | ENSG00000011132 | *APBA3* | 19 | 3485842 | 3712673 | 226831 | 0.289 | 6.51e−04 |
| 40 | LWK | rs1017943 | ILMN_1659651 | ENSG00000184428 | *TOP1MT* | 8 | 144509891 | 144488425 | 21466 | −0.385 | 3.52e−04 |
| 41 | LWK | rs10406628 | ILMN_1669842 | ENSG00000167670 | *CHAF1A* | 19 | 4481261 | 4353660 | 127601 | 0.359 | 9.39e−04 |
| 42 | GIH | rs10406797 | ILMN_1752423 | ENSG00000126934 | *MAP2K2* | 19 | 4451205 | 4075126 | 376079 | −0.359 | 9.32e−04 |
| 43 | JPT | rs10409289 | ILMN_1794823 | ENSG00000188171 | *ZNF626* | 19 | 20598664 | 20636246 | 37582 | 0.479 | 8.93e−06 |
| 44 | MEX | rs10415416 | ILMN_1669572 | ENSG00000070423 | *RNF126* | 19 | 441081 | 614227 | 173146 | −0.521 | 2.84e−04 |
| 45 | LWK | rs10421633 | ILMN_1794823 | ENSG00000188171 | *ZNF626* | 19 | 20585008 | 20636246 | 51238 | 0.431 | 5.30e−05 |
| 46 | GIH | rs10425311 | ILMN_1794823 | ENSG00000188171 | *ZNF626* | 19 | 20618816 | 20636246 | 17430 | 0.414 | 1.11e−04 |
| 47 | JPT | rs1042541 | ILMN_1654943 | ENSG00000089685 | *BIRC5* | 17 | 73732965 | 73721872 | 11093 | −0.416 | 1.01e−04 |
| 48 | JPT | rs1042542 | ILMN_1654943 | ENSG00000089685 | *BIRC5* | 17 | 73733023 | 73721872 | 11151 | 0.388 | 3.13e−04 |
| 49 | JPT | rs10425463 | ILMN_1794823 | ENSG00000188171 | *ZNF626* | 19 | 20618710 | 20636246 | 17536 | 0.486 | 3.63e−06 |
| 50 | CHB | rs10432039 | ILMN_1791106 | ENSG00000068097 | *HEATR6* | 17 | 55814579 | 55511074 | 303505 | 0.458 | 1.96e−05 |
| 51 | MKK | rs1043789 | ILMN_1685676 | ENSG00000162222 | *TTC9C* | 11 | 61917305 | 62252160 | 334855 | −0.3 | 3.45e−04 |
| 52 | CEU | rs1044115 | ILMN_1725683 | ENSG00000172728 | *FUT10* | 8 | 33576911 | 33450206 | 126705 | −0.395 | 2.11e−05 |
| 53 | CEU | rs10464811 | ILMN_1725683 | ENSG00000172728 | *FUT10* | 8 | 33420315 | 33450206 | 29891 | 0.523 | 7.65e−09 |
| 54 | LWK | rs10481333 | ILMN_1714081 | ENSG00000008853 | *RHOBTB2* | 8 | 23742889 | 22913059 | 829830 | −0.368 | 6.65e−04 |
| 55 | YRI | rs1049832 | ILMN_1690490 | ENSG00000181638 | *GLI4* | 8 | 144768184 | 144400484 | 367700 | 0.32 | 7.36e−04 |
| 56 | GIH | rs10503946 | ILMN_1725683 | ENSG00000172728 | *FUT10* | 8 | 33391963 | 33450206 | 58243 | −0.411 | 1.24e−04 |
| 57 | CEU | rs10504039 | ILMN_1684694 | ENSG00000029534 | *ANK1* | 8 | 41056808 | 41873155 | 816347 | 0.325 | 6.03e−04 |
| 58 | MEX | rs10504047 | ILMN_1740752 | ENSG00000158669 | *AGPAT6* | 8 | 41835520 | 41554876 | 280644 | 0.494 | 5.55e−04 |
| 59 | MKK | rs10504048 | ILMN_1676946 | ENSG00000070718 | *AP3M2* | 8 | 41924406 | 42129748 | 205342 | 0.277 | 9.99e−04 |
| 60 | YRI | rs10504050 | ILMN_1676946 | ENSG00000070718 | *AP3M2* | 8 | 42146421 | 42129748 | 16673 | 0.363 | 1.14e−04 |
| 61 | GIH | rs10505035 | ILMN_1659801 | ENSG00000155097 | *ATP6V1C1* | 8 | 103844351 | 104102445 | 258094 | −0.357 | 9.96e−04 |
| 62 | CEU | rs10515175 | ILMN_1791106 | ENSG00000068097 | *HEATR6* | 17 | 55495735 | 55511074 | 15339 | −0.314 | 8.84e−04 |
| 63 | CEU | rs10515177 | ILMN_1791106 | ENSG00000068097 | *HEATR6* | 17 | 55522267 | 55511074 | 11193 | 0.426 | 3.80e−06 |
| 64 | JPT | rs1054533 | ILMN_1798083 | ENSG00000085872 | *CHERP* | 19 | 16865049 | 16514263 | 350786 | 0.361 | 9.93e−04 |
| 65 | GIH | rs1055181 | ILMN_1782751 | ENSG00000181638 | *GLI4* | 8 | 144537348 | 144400484 | 136864 | −0.37 | 6.30e−04 |
| 66 | LWK | rs1055919 | ILMN_1654246 | ENSG00000077463 | *SIRT6* | 19 | 4803137 | 4133596 | 669541 | −0.375 | 5.13e−04 |
| 67 | LWK | rs1056144 | ILMN_1790741 | ENSG00000070423 | *RNF126* | 19 | 925967 | 614227 | 311740 | 0.385 | 3.58e−04 |
| 68 | MKK | rs1057083 | ILMN_1722390 | ENSG00000104472 | *CHRAC1* | 8 | 141587219 | 141590586 | 3367 | 0.311 | 2.01e−04 |
| 69 | LWK | rs1060832 | ILMN_1722390 | ENSG00000104472 | *CHRAC1* | 8 | 141611070 | 141590586 | 20484 | −0.467 | 9.60e−06 |
| 70 | GIH | rs1062391 | ILMN_1782021 | ENSG00000181135 | *ZNF707* | 8 | 144733496 | 144824516 | 91020 | 0.392 | 2.72e−04 |
| 71 | CEU | rs1063739 | ILMN_1811433 | ENSG00000161016 | *RPL8* | 8 | 145700535 | 145988609 | 288074 | −0.338 | 3.21e−04 |
| 72 | CHB | rs107251 | ILMN_1654246 | ENSG00000077463 | *SIRT6* | 19 | 4127085 | 4133596 | 6511 | 0.5 | 2.66e−06 |
| 73 | LWK | rs1073640 | ILMN_1807501 | ENSG00000147536 | *GINS4* | 8 | 40607130 | 41505925 | 898795 | 0.364 | 7.78e−04 |
| 74 | GIH | rs1075478 | ILMN_1729816 | ENSG00000078668 | *VDAC3* | 8 | 42805929 | 42368547 | 437382 | 0.389 | 3.02e−04 |
| 75 | CEU | rs10791899 | ILMN_1740240 | ENSG00000173599 | *PC* | 11 | 66610041 | 66482423 | 127618 | 0.36 | 1.20e−04 |
| 76 | LWK | rs10852744 | ILMN_1762071 | ENSG00000141219 | *C17orf80* | 17 | 68655122 | 68740953 | 85831 | 0.388 | 3.21e−04 |
| 77 | MKK | rs10866915 | ILMN_1659651 | ENSG00000184428 | *TOP1MT* | 8 | 144961160 | 144488425 | 472735 | −0.279 | 9.20e−04 |
| 78 | MKK | rs10956986 | ILMN_1703635 | ENSG00000104529 | *EEF1D* | 8 | 143883603 | 144750726 | 867123 | −0.291 | 5.68e−04 |
| 79 | MKK | rs10958663 | ILMN_1805221 | ENSG00000147533 | *GOLGA7* | 8 | 41135797 | 41467238 | 331441 | 0.302 | 3.23e−04 |
| 80 | LWK | rs11077912 | ILMN_1781001 | ENSG00000184557 | *SOCS3* | 17 | 73051366 | 73867753 | 816387 | −0.382 | 4.00e−04 |
| 81 | GIH | rs11079372 | ILMN_1687703 | ENSG00000108395 | *TRIM37* | 17 | 54766647 | 54539011 | 227636 | 0.381 | 4.06e−04 |
| 82 | GIH | rs11086017 | ILMN_1708369 | ENSG00000127527 | *EPS15L1* | 19 | 15946596 | 16443768 | 497172 | 0.389 | 3.04e−04 |
| 83 | GIH | rs1113022 | ILMN_1725683 | ENSG00000172728 | *FUT10* | 8 | 33335646 | 33450206 | 114560 | −0.457 | 1.56e−05 |
| 84 | YRI | rs11135693 | ILMN_1699473 | ENSG00000158941 | *KIAA1967* | 8 | 22981099 | 22518202 | 462897 | −0.319 | 7.60e−04 |
| 85 | YRI | rs11135694 | ILMN_1699473 | ENSG00000158941 | *KIAA1967* | 8 | 22981460 | 22518202 | 463258 | 0.326 | 6.09e−04 |
| 86 | GIH | rs11135995 | ILMN_1699265 | ENSG00000120889 | *TNFRSF10B* | 8 | 22256093 | 22982637 | 726544 | −0.388 | 3.20e−04 |
| 87 | CEU | rs11136002 | ILMN_1786345 | ENSG00000158941 | *KIAA1967* | 8 | 22273027 | 22518202 | 245175 | −0.313 | 9.19e−04 |
| 88 | CEU | rs11136295 | ILMN_1696752 | ENSG00000014164 | *ZC3H3* | 8 | 144589192 | 144694746 | 105554 | 0.346 | 2.27e−04 |
| 89 | YRI | rs11136315 | ILMN_1782021 | ENSG00000181135 | *ZNF707* | 8 | 144849455 | 144824516 | 24939 | 0.448 | 1.14e−06 |
| 90 | MEX | rs11136331 | ILMN_1696752 | ENSG00000014164 | *ZC3H3* | 8 | 145038946 | 144694746 | 344200 | −0.479 | 8.80e−04 |
| 91 | MEX | rs11167062 | ILMN_1714364 | ENSG00000169398 | *PTK2* | 8 | 142528214 | 142080514 | 447700 | 0.541 | 1.52e−04 |
| 92 | LWK | rs1118967 | ILMN_1794823 | ENSG00000188171 | *ZNF626* | 19 | 20654281 | 20636246 | 18035 | −0.383 | 3.86e−04 |
| 93 | LWK | rs11248216 | ILMN_1757230 | ENSG00000196150 | *ZNF250* | 8 | 146125907 | 146097650 | 28257 | −0.384 | 3.76e−04 |
| 94 | LWK | rs1127096 | ILMN_1745271 | ENSG00000178896 | *EXOSC4* | 8 | 145212460 | 145205510 | 6950 | 0.399 | 2.03e−04 |
| 95 | YRI | rs11544484 | ILMN_1659651 | ENSG00000184428 | *TOP1MT* | 8 | 144478080 | 144488425 | 10345 | −0.388 | 3.27e−05 |
| 96 | LWK | rs11546144 | ILMN_1745271 | ENSG00000178896 | *EXOSC4* | 8 | 145233811 | 145205510 | 28301 | 0.403 | 1.73e−04 |
| 97 | GIH | rs1158001 | ILMN_1725683 | ENSG00000172728 | *FUT10* | 8 | 33307912 | 33450206 | 142294 | −0.471 | 8.12e−06 |
| 98 | YRI | rs11658299 | ILMN_1654943 | ENSG00000089685 | *BIRC5* | 17 | 74337317 | 73721872 | 615445 | −0.331 | 4.65e−04 |
| 99 | JPT | rs11666306 | ILMN_1702507 | ENSG00000130517 | *PGPEP1* | 19 | 17351013 | 18312408 | 961395 | 0.364 | 7.65e−04 |
| 100 | GIH | rs11666309 | ILMN_1752333 | ENSG00000127526 | *SLC35E1* | 19 | 17312187 | 16544193 | 767994 | −0.357 | 9.85e−04 |
| 101 | YRI | rs11668840 | ILMN_1798083 | ENSG00000085872 | *CHERP* | 19 | 17260625 | 16514263 | 746362 | −0.332 | 4.46e−04 |
| 102 | GIH | rs11773930 | ILMN_1714081 | ENSG00000008853 | *RHOBTB2* | 8 | 22243800 | 22913059 | 669259 | −0.357 | 9.83e−04 |
| 103 | CHB | rs11775009 | ILMN_1724611 | ENSG00000184428 | *TOP1MT* | 8 | 144005183 | 144488425 | 483242 | −0.364 | 9.10e−04 |
| 104 | MEX | rs11775176 | ILMN_1699265 | ENSG00000120889 | *TNFRSF10B* | 8 | 22948979 | 22982637 | 33658 | −0.484 | 7.53e−04 |
| 105 | LWK | rs11775256 | ILMN_1786345 | ENSG00000158941 | *KIAA1967* | 8 | 23107430 | 22518202 | 589228 | −0.389 | 3.02e−04 |
| 106 | YRI | rs11775744 | ILMN_1715668 | ENSG00000147804 | *SLC39A4* | 8 | 144720335 | 145613081 | 892746 | −0.317 | 8.78e−04 |
| 107 | GIH | rs11776189 | ILMN_1727618 | ENSG00000156170 | *C8orf38* | 8 | 96105917 | 95977217 | 128700 | 0.4 | 1.96e−04 |
| 108 | LWK | rs11776272 | ILMN_1690546 | ENSG00000120910 | *PPP3CC* | 8 | 21940490 | 22354541 | 414051 | −0.416 | 1.02e−04 |
| 109 | YRI | rs11776602 | ILMN_1768273 | ENSG00000104356 | *POP1* | 8 | 99083471 | 99199244 | 115773 | 0.326 | 5.69e−04 |
| 110 | MKK | rs11777351 | ILMN_1796508 | ENSG00000184428 | *TOP1MT* | 8 | 144671686 | 144488425 | 183261 | −0.302 | 3.18e−04 |
| 111 | MEX | rs11778831 | ILMN_1666022 | ENSG00000173530 | *TNFRSF10D* | 8 | 22391033 | 23077485 | 686452 | 0.49 | 6.38e−04 |
| 112 | CEU | rs11778875 | ILMN_1684694 | ENSG00000029534 | *ANK1* | 8 | 41020375 | 41873155 | 852780 | 0.332 | 4.49e−04 |
| 113 | GIH | rs11779506 | ILMN_1727618 | ENSG00000156170 | *C8orf38* | 8 | 96144078 | 95977217 | 166861 | 0.384 | 3.64e−04 |
| 114 | LWK | rs11780345 | ILMN_1786345 | ENSG00000158941 | *KIAA1967* | 8 | 23107911 | 22518202 | 589709 | 0.386 | 3.40e−04 |
| 115 | GIH | rs11780931 | ILMN_1727618 | ENSG00000156170 | *C8orf38* | 8 | 96113005 | 95977217 | 135788 | 0.4 | 1.96e−04 |
| 116 | YRI | rs11781035 | ILMN_1722390 | ENSG00000104472 | *CHRAC1* | 8 | 140840343 | 141590586 | 750243 | −0.32 | 7.31e−04 |
| 117 | GIH | rs11781082 | ILMN_1659651 | ENSG00000184428 | *TOP1MT* | 8 | 143996903 | 144488425 | 491522 | 0.358 | 9.69e−04 |
| 118 | LWK | rs11781095 | ILMN_1804789 | ENSG00000158941 | *KIAA1967* | 8 | 22657732 | 22518202 | 139530 | 0.361 | 8.76e−04 |
| 119 | MEX | rs11781250 | ILMN_1714364 | ENSG00000169398 | *PTK2* | 8 | 142529677 | 142080514 | 449163 | −0.528 | 1.96e−04 |
| 120 | CEU | rs11781871 | ILMN_1715969 | ENSG00000147454 | *SLC25A37* | 8 | 23313035 | 23442308 | 129273 | −0.313 | 9.04e−04 |
| 121 | CHB | rs11782029 | ILMN_1690490 | ENSG00000181638 | *GLI4* | 8 | 144295968 | 144400484 | 104516 | 0.366 | 8.30e−04 |
| 122 | CHB | rs11782054 | ILMN_1722390 | ENSG00000104472 | *CHRAC1* | 8 | 141567314 | 141590586 | 23272 | −0.385 | 4.23e−04 |
| 123 | CEU | rs11783095 | ILMN_1695404 | ENSG00000160932 | *LY6E* | 8 | 144579911 | 144171274 | 408637 | −0.328 | 4.96e−04 |
| 124 | MEX | rs11783414 | ILMN_1804789 | ENSG00000158941 | *KIAA1967* | 8 | 21829330 | 22518202 | 688872 | 0.521 | 2.41e−04 |
| 125 | MEX | rs11783730 | ILMN_1666022 | ENSG00000173530 | *TNFRSF10D* | 8 | 22300842 | 23077485 | 776643 | 0.478 | 8.92e−04 |
| 126 | MKK | rs11784090 | ILMN_1806304 | ENSG00000197217 | *ENTPD4* | 8 | 23433106 | 23371115 | 61991 | −0.278 | 9.68e−04 |
| 127 | JPT | rs11784421 | ILMN_1811433 | ENSG00000161016 | *RPL8* | 8 | 145856601 | 145988609 | 132008 | 0.363 | 8.02e−04 |
| 128 | MEX | rs11784679 | ILMN_1715969 | ENSG00000147454 | *SLC25A37* | 8 | 22887095 | 23442308 | 555213 | −0.49 | 6.38e−04 |
| 129 | CEU | rs11784860 | ILMN_1811433 | ENSG00000161016 | *RPL8* | 8 | 145984603 | 145988609 | 4006 | −0.625 | 3.93e−13 |
| 130 | MEX | rs11785599 | ILMN_1690546 | ENSG00000120910 | *PPP3CC* | 8 | 22948219 | 22354541 | 593678 | −0.487 | 6.96e−04 |
| 131 | YRI | rs11785807 | ILMN_1768273 | ENSG00000104356 | *POP1* | 8 | 99080874 | 99199244 | 118370 | 0.353 | 1.75e−04 |
| 132 | GIH | rs11787224 | ILMN_1806304 | ENSG00000197217 | *ENTPD4* | 8 | 23357285 | 23371115 | 13830 | 0.403 | 1.74e−04 |
| 133 | GIH | rs11870711 | ILMN_1781001 | ENSG00000184557 | *SOCS3* | 17 | 74858863 | 73867753 | 991110 | −0.385 | 3.55e−04 |
| 134 | GIH | rs11878868 | ILMN_1752423 | ENSG00000126934 | *MAP2K2* | 19 | 4124637 | 4075126 | 49511 | 0.371 | 5.96e−04 |
| 135 | MEX | rs11880324 | ILMN_1752333 | ENSG00000127526 | *SLC35E1* | 19 | 16946341 | 16544193 | 402148 | −0.489 | 6.56e−04 |
| 136 | MKK | rs11986960 | ILMN_1764721 | ENSG00000161016 | *RPL8* | 8 | 145172093 | 145988609 | 816516 | 0.28 | 8.72e−04 |
| 137 | MKK | rs11987209 | ILMN_1870804 | ENSG00000204763 | *CYHR1* | 8 | 144766196 | 145653832 | 887636 | −0.31 | 2.21e−04 |
| 138 | MKK | rs11988619 | ILMN_1759084 | ENSG00000164941 | *INTS8* | 8 | 96618980 | 95904710 | 714270 | 0.332 | 7.08e−05 |
| 139 | GIH | rs11989052 | ILMN_1811433 | ENSG00000161016 | *RPL8* | 8 | 145130025 | 145988609 | 858584 | 0.358 | 9.64e−04 |
| 140 | MKK | rs11990460 | ILMN_1676946 | ENSG00000070718 | *AP3M2* | 8 | 41957660 | 42129748 | 172088 | −0.337 | 5.45e−05 |
| 141 | MKK | rs11991402 | ILMN_1676946 | ENSG00000070718 | *AP3M2* | 8 | 41930005 | 42129748 | 199743 | 0.337 | 5.45e−05 |
| 142 | YRI | rs11992718 | ILMN_1722390 | ENSG00000104472 | *CHRAC1* | 8 | 140831665 | 141590586 | 758921 | 0.39 | 3.33e−05 |
| 143 | MKK | rs11993268 | ILMN_1764721 | ENSG00000161016 | *RPL8* | 8 | 145182465 | 145988609 | 806144 | −0.308 | 2.42e−04 |
| 144 | YRI | rs11994710 | ILMN_1811433 | ENSG00000161016 | *RPL8* | 8 | 146241465 | 145988609 | 252856 | 0.327 | 5.77e−04 |
| 145 | GIH | rs11994923 | ILMN_1725683 | ENSG00000172728 | *FUT10* | 8 | 33377907 | 33450206 | 72299 | 0.391 | 2.84e−04 |
| 146 | JPT | rs11995026 | ILMN_1727618 | ENSG00000156170 | *C8orf38* | 8 | 96826619 | 95977217 | 849402 | 0.36 | 8.81e−04 |
| 147 | MKK | rs11995708 | ILMN_1811433 | ENSG00000161016 | *RPL8* | 8 | 146025077 | 145988609 | 36468 | 0.278 | 9.60e−04 |
| 148 | MKK | rs11997383 | ILMN_1805221 | ENSG00000147533 | *GOLGA7* | 8 | 40574573 | 41467238 | 892665 | −0.28 | 9.37e−04 |
| 149 | CEU | rs1209853 | ILMN_1811433 | ENSG00000161016 | *RPL8* | 8 | 146073714 | 145988609 | 85105 | 0.354 | 1.62e−04 |
| 150 | CEU | rs1209857 | ILMN_1811433 | ENSG00000161016 | *RPL8* | 8 | 146070655 | 145988609 | 82046 | 0.372 | 6.81e−05 |
| 151 | CEU | rs1209874 | ILMN_1811433 | ENSG00000161016 | *RPL8* | 8 | 146049668 | 145988609 | 61059 | 0.338 | 3.25e−04 |
| 152 | CEU | rs1209879 | ILMN_1811433 | ENSG00000161016 | *RPL8* | 8 | 146047512 | 145988609 | 58903 | 0.367 | 8.59e−05 |
| 153 | CEU | rs1209881 | ILMN_1811433 | ENSG00000161016 | *RPL8* | 8 | 146046636 | 145988609 | 58027 | −0.385 | 3.63e−05 |
| 154 | CEU | rs12114096 | ILMN_1684694 | ENSG00000029534 | *ANK1* | 8 | 40984447 | 41873155 | 888708 | −0.313 | 9.29e−04 |
| 155 | CEU | rs12155581 | ILMN_1725683 | ENSG00000172728 | *FUT10* | 8 | 33407344 | 33450206 | 42862 | −0.518 | 7.86e−09 |
| 156 | YRI | rs12334514 | ILMN_1714364 | ENSG00000169398 | *PTK2* | 8 | 142168429 | 142080514 | 87915 | −0.321 | 7.15e−04 |
| 157 | CEU | rs12386778 | ILMN_1725683 | ENSG00000172728 | *FUT10* | 8 | 33503569 | 33450206 | 53363 | −0.417 | 8.95e−06 |
| 158 | MEX | rs12451377 | ILMN_1710082 | ENSG00000089685 | *BIRC5* | 17 | 74189805 | 73721872 | 467933 | −0.487 | 6.90e−04 |
| 159 | LWK | rs12461827 | ILMN_1686610 | ENSG00000011132 | *APBA3* | 19 | 3096887 | 3712673 | 615786 | 0.416 | 1.04e−04 |
| 160 | YRI | rs12540996 | ILMN_1811433 | ENSG00000161016 | *RPL8* | 8 | 146233985 | 145988609 | 245376 | 0.327 | 5.77e−04 |
| 161 | GIH | rs12541516 | ILMN_1725683 | ENSG00000172728 | *FUT10* | 8 | 33311895 | 33450206 | 138311 | 0.405 | 1.59e−04 |
| 162 | YRI | rs12542316 | ILMN_1811433 | ENSG00000161016 | *RPL8* | 8 | 146235518 | 145988609 | 246909 | 0.313 | 9.58e−04 |
| 163 | CEU | rs12542335 | ILMN_1811433 | ENSG00000161016 | *RPL8* | 8 | 146090023 | 145988609 | 101414 | 0.397 | 1.91e−05 |
| 164 | GIH | rs12542920 | ILMN_1725683 | ENSG00000172728 | *FUT10* | 8 | 33304753 | 33450206 | 145453 | −0.471 | 8.12e−06 |
| 165 | CHB | rs12544414 | ILMN_1786345 | ENSG00000158941 | *KIAA1967* | 8 | 23166729 | 22518202 | 648527 | −0.371 | 7.05e−04 |
| 166 | GIH | rs12545121 | ILMN_1775573 | ENSG00000156162 | *DPY19L4* | 8 | 96553323 | 95801327 | 751996 | −0.37 | 6.63e−04 |
| 167 | YRI | rs12545575 | ILMN_1699473 | ENSG00000158941 | *KIAA1967* | 8 | 22330562 | 22518202 | 187640 | 0.315 | 9.08e−04 |
| 168 | CEU | rs12545740 | ILMN_1725683 | ENSG00000172728 | *FUT10* | 8 | 33574842 | 33450206 | 124636 | 0.452 | 8.32e−07 |
| 169 | MEX | rs12545909 | ILMN_1666022 | ENSG00000173530 | *TNFRSF10D* | 8 | 22365997 | 23077485 | 711488 | −0.483 | 7.85e−04 |
| 170 | GIH | rs12546032 | ILMN_1725683 | ENSG00000172728 | *FUT10* | 8 | 33306644 | 33450206 | 143562 | −0.471 | 8.12e−06 |
| 171 | GIH | rs12546272 | ILMN_1804169 | ENSG00000179526 | *SHARPIN* | 8 | 144727330 | 145231128 | 503798 | −0.369 | 7.03e−04 |
| 172 | YRI | rs12546486 | ILMN_1727618 | ENSG00000156170 | *C8orf38* | 8 | 95195882 | 95977217 | 781335 | 0.322 | 6.85e−04 |
| 173 | LWK | rs12547064 | ILMN_1676946 | ENSG00000070718 | *AP3M2* | 8 | 42138770 | 42129748 | 9022 | 0.394 | 2.52e−04 |
| 174 | GIH | rs12547379 | ILMN_1659801 | ENSG00000155097 | *ATP6V1C1* | 8 | 103775535 | 104102445 | 326910 | 0.359 | 9.39e−04 |
| 175 | MKK | rs12547483 | ILMN_1717420 | ENSG00000104343 | *UBE2W* | 8 | 75217559 | 74923937 | 293622 | 0.282 | 8.20e−04 |
| 176 | CEU | rs12548006 | ILMN_1789138 | ENSG00000169499 | *PLEKHA2* | 8 | 39031824 | 38877986 | 153838 | −0.33 | 6.70e−04 |
| 177 | MKK | rs12549294 | ILMN_1807501 | ENSG00000147536 | *GINS4* | 8 | 41625537 | 41505925 | 119612 | 0.283 | 7.89e−04 |
| 178 | GIH | rs12549316 | ILMN_1725683 | ENSG00000172728 | *FUT10* | 8 | 33384253 | 33450206 | 65953 | −0.412 | 1.23e−04 |
| 179 | CEU | rs12549460 | ILMN_1684694 | ENSG00000029534 | *ANK1* | 8 | 41062994 | 41873155 | 810161 | 0.325 | 5.64e−04 |
| 180 | MKK | rs12549902 | ILMN_1807501 | ENSG00000147536 | *GINS4* | 8 | 41628416 | 41505925 | 122491 | −0.312 | 1.94e−04 |
| 181 | LWK | rs12550574 | ILMN_1690546 | ENSG00000120910 | *PPP3CC* | 8 | 21772807 | 22354541 | 581734 | 0.372 | 5.88e−04 |
| 182 | LWK | rs12550729 | ILMN_1745271 | ENSG00000178896 | *EXOSC4* | 8 | 145227396 | 145205510 | 21886 | −0.403 | 1.73e−04 |
| 183 | MKK | rs12601766 | ILMN_1654943 | ENSG00000089685 | *BIRC5* | 17 | 73062655 | 73721872 | 659217 | 0.278 | 9.71e−04 |
| 184 | GIH | rs12602498 | ILMN_1711327 | ENSG00000108395 | *TRIM37* | 17 | 53628207 | 54539011 | 910804 | 0.369 | 6.51e−04 |
| 185 | LWK | rs12675383 | ILMN_1741841 | ENSG00000164944 | *KIAA1429* | 8 | 96541266 | 95634864 | 906402 | 0.382 | 4.01e−04 |
| 186 | MKK | rs12677785 | ILMN_1722390 | ENSG00000104472 | *CHRAC1* | 8 | 142322438 | 141590586 | 731852 | 0.311 | 2.12e−04 |
| 187 | CHB | rs12677931 | ILMN_1767839 | ENSG00000181638 | *GLI4* | 8 | 144273039 | 144400484 | 127445 | −0.363 | 9.48e−04 |
| 188 | MEX | rs12681533 | ILMN_1725683 | ENSG00000172728 | *FUT10* | 8 | 33259628 | 33450206 | 190578 | 0.493 | 5.81e−04 |
| 189 | CEU | rs12682118 | ILMN_1725683 | ENSG00000172728 | *FUT10* | 8 | 33490838 | 33450206 | 40632 | 0.483 | 1.02e−07 |
| 190 | LWK | rs1273530 | ILMN_1708369 | ENSG00000127527 | *EPS15L1* | 19 | 15510534 | 16443768 | 933234 | −0.365 | 7.39e−04 |
| 191 | CEU | rs12788752 | ILMN_1740240 | ENSG00000173599 | *PC* | 11 | 66894303 | 66482423 | 411880 | 0.324 | 5.93e−04 |
| 192 | MKK | rs1292067 | ILMN_1791106 | ENSG00000068097 | *HEATR6* | 17 | 55285428 | 55511074 | 225646 | −0.342 | 4.42e−05 |
| 193 | GIH | rs12940527 | ILMN_1762071 | ENSG00000141219 | *C17orf80* | 17 | 68496877 | 68740953 | 244076 | −0.369 | 8.25e−04 |
| 194 | GIH | rs12941308 | ILMN_1762071 | ENSG00000141219 | *C17orf80* | 17 | 69078552 | 68740953 | 337599 | 0.382 | 3.93e−04 |
| 195 | YRI | rs12971592 | ILMN_1794823 | ENSG00000188171 | *ZNF626* | 19 | 20643736 | 20636246 | 7490 | 0.366 | 9.65e−05 |
| 196 | JPT | rs12972417 | ILMN_1702507 | ENSG00000130517 | *PGPEP1* | 19 | 17386314 | 18312408 | 926094 | −0.358 | 9.54e−04 |
| 197 | MKK | rs12976454 | ILMN_1686610 | ENSG00000011132 | *APBA3* | 19 | 3495971 | 3712673 | 216702 | −0.305 | 3.40e−04 |
| 198 | MKK | rs12984683 | ILMN_1794823 | ENSG00000188171 | *ZNF626* | 19 | 20810397 | 20636246 | 174151 | 0.323 | 1.19e−04 |
| 199 | MKK | rs13248647 | ILMN_1676946 | ENSG00000070718 | *AP3M2* | 8 | 41886411 | 42129748 | 243337 | −0.332 | 6.93e−05 |
| 200 | GIH | rs13248759 | ILMN_1806304 | ENSG00000197217 | *ENTPD4* | 8 | 23381978 | 23371115 | 10863 | 0.429 | 5.69e−05 |
| 201 | MKK | rs13248926 | ILMN_1806304 | ENSG00000197217 | *ENTPD4* | 8 | 23231197 | 23371115 | 139918 | 0.303 | 3.03e−04 |
| 202 | MEX | rs13249908 | ILMN_1704873 | ENSG00000154582 | *TCEB1* | 8 | 75176984 | 75046956 | 130028 | −0.499 | 4.78e−04 |
| 203 | LWK | rs13250446 | ILMN_1745271 | ENSG00000178896 | *EXOSC4* | 8 | 145236835 | 145205510 | 31325 | −0.369 | 6.56e−04 |
| 204 | MKK | rs13252406 | ILMN_1796508 | ENSG00000184428 | *TOP1MT* | 8 | 144984291 | 144488425 | 495866 | 0.281 | 8.50e−04 |
| 205 | CEU | rs13254911 | ILMN_1811433 | ENSG00000161016 | *RPL8* | 8 | 145699543 | 145988609 | 289066 | 0.35 | 2.07e−04 |
| 206 | GIH | rs13255110 | ILMN_1706386 | ENSG00000147804 | *SLC39A4* | 8 | 145017780 | 145613081 | 595301 | −0.404 | 1.68e−04 |
| 207 | MKK | rs13257452 | ILMN_1676946 | ENSG00000070718 | *AP3M2* | 8 | 41974358 | 42129748 | 155390 | −0.297 | 4.04e−04 |
| 208 | GIH | rs13259097 | ILMN_1714081 | ENSG00000008853 | *RHOBTB2* | 8 | 22189689 | 22913059 | 723370 | −0.383 | 3.87e−04 |
| 209 | MKK | rs13259317 | ILMN_1806304 | ENSG00000197217 | *ENTPD4* | 8 | 23231036 | 23371115 | 140079 | 0.294 | 4.73e−04 |
| 210 | CEU | rs13260283 | ILMN_1690546 | ENSG00000120910 | *PPP3CC* | 8 | 21767137 | 22354541 | 587404 | 0.312 | 9.44e−04 |
| 211 | YRI | rs13260358 | ILMN_1740752 | ENSG00000158669 | *AGPAT6* | 8 | 41926532 | 41554876 | 371656 | 0.317 | 8.32e−04 |
| 212 | JPT | rs13262489 | ILMN_1806304 | ENSG00000197217 | *ENTPD4* | 8 | 23748595 | 23371115 | 377480 | −0.379 | 4.54e−04 |
| 213 | JPT | rs13265349 | ILMN_1695404 | ENSG00000160932 | *LY6E* | 8 | 143414319 | 144171274 | 756955 | 0.37 | 6.18e−04 |
| 214 | JPT | rs13269890 | ILMN_1727618 | ENSG00000156170 | *C8orf38* | 8 | 96831450 | 95977217 | 854233 | −0.375 | 5.70e−04 |
| 215 | LWK | rs13274431 | ILMN_1695404 | ENSG00000160932 | *LY6E* | 8 | 144385752 | 144171274 | 214478 | 0.376 | 4.94e−04 |
| 216 | MKK | rs13274943 | ILMN_1717420 | ENSG00000104343 | *UBE2W* | 8 | 75564650 | 74923937 | 640713 | 0.297 | 4.79e−04 |
| 217 | CEU | rs13275065 | ILMN_1811433 | ENSG00000161016 | *RPL8* | 8 | 145789489 | 145988609 | 199120 | 0.367 | 9.37e−05 |
| 218 | MKK | rs13276409 | ILMN_1676946 | ENSG00000070718 | *AP3M2* | 8 | 41880964 | 42129748 | 248784 | −0.282 | 7.99e−04 |
| 219 | CEU | rs13276958 | ILMN_1676305 | ENSG00000169398 | *PTK2* | 8 | 141652078 | 142080514 | 428436 | −0.326 | 5.40e−04 |
| 220 | GIH | rs13279040 | ILMN_1727618 | ENSG00000156170 | *C8orf38* | 8 | 96149565 | 95977217 | 172348 | −0.384 | 3.64e−04 |
| 221 | GIH | rs13281023 | ILMN_1662427 | ENSG00000184489 | *PTP4A3* | 8 | 143411862 | 142501189 | 910673 | −0.365 | 7.39e−04 |
| 222 | MKK | rs13282703 | ILMN_1676946 | ENSG00000070718 | *AP3M2* | 8 | 41945974 | 42129748 | 183774 | 0.335 | 5.98e−05 |
| 223 | CHB | rs1355250 | ILMN_1791106 | ENSG00000068097 | *HEATR6* | 17 | 55860381 | 55511074 | 349307 | −0.458 | 1.96e−05 |
| 224 | MKK | rs13748 | ILMN_1676946 | ENSG00000070718 | *AP3M2* | 8 | 41914091 | 42129748 | 215657 | 0.335 | 5.98e−05 |
| 225 | MEX | rs1385229 | ILMN_1714108 | ENSG00000164938 | *TP53INP1* | 8 | 96840723 | 96030770 | 809953 | −0.477 | 9.21e−04 |
| 226 | MKK | rs1398270 | ILMN_1659801 | ENSG00000155097 | *ATP6V1C1* | 8 | 104136181 | 104102445 | 33736 | −0.292 | 5.02e−04 |
| 227 | MKK | rs1398271 | ILMN_1659801 | ENSG00000155097 | *ATP6V1C1* | 8 | 104136328 | 104102445 | 33883 | −0.292 | 5.02e−04 |
| 228 | LWK | rs1429949 | ILMN_1778673 | ENSG00000147533 | *GOLGA7* | 8 | 40536590 | 41467238 | 930648 | 0.409 | 1.39e−04 |
| 229 | CEU | rs1440530 | ILMN_1725683 | ENSG00000172728 | *FUT10* | 8 | 33534036 | 33450206 | 83830 | 0.493 | 5.28e−08 |
| 230 | CEU | rs1465959 | ILMN_1725683 | ENSG00000172728 | *FUT10* | 8 | 33582222 | 33450206 | 132016 | 0.363 | 1.04e−04 |
| 231 | LWK | rs1469587 | ILMN_1762071 | ENSG00000141219 | *C17orf80* | 17 | 68247582 | 68740953 | 493371 | −0.364 | 7.75e−04 |
| 232 | GIH | rs1474034 | ILMN_1725683 | ENSG00000172728 | *FUT10* | 8 | 33326114 | 33450206 | 124092 | 0.475 | 6.44e−06 |
| 233 | GIH | rs1474035 | ILMN_1725683 | ENSG00000172728 | *FUT10* | 8 | 33347704 | 33450206 | 102502 | 0.378 | 4.69e−04 |
| 234 | CEU | rs1480000 | ILMN_1811433 | ENSG00000161016 | *RPL8* | 8 | 145672815 | 145988609 | 315794 | −0.361 | 1.22e−04 |
| 235 | MEX | rs1482337 | ILMN_1666022 | ENSG00000173530 | *TNFRSF10D* | 8 | 22451454 | 23077485 | 626031 | −0.539 | 1.32e−04 |
| 236 | MKK | rs1494272 | ILMN_1663257 | ENSG00000155097 | *ATP6V1C1* | 8 | 104264149 | 104102445 | 161704 | 0.29 | 6.38e−04 |
| 237 | JPT | rs1508146 | ILMN_1654943 | ENSG00000089685 | *BIRC5* | 17 | 73734158 | 73721872 | 12286 | 0.388 | 3.13e−04 |
| 238 | MKK | rs1512350 | ILMN_1659801 | ENSG00000155097 | *ATP6V1C1* | 8 | 104129436 | 104102445 | 26991 | −0.284 | 7.29e−04 |
| 239 | CEU | rs1530205 | ILMN_1725683 | ENSG00000172728 | *FUT10* | 8 | 33522604 | 33450206 | 72398 | 0.503 | 2.53e−08 |
| 240 | CEU | rs1530344 | ILMN_1725683 | ENSG00000172728 | *FUT10* | 8 | 33562618 | 33450206 | 112412 | 0.411 | 8.83e−06 |
| 241 | CEU | rs1533470 | ILMN_1725683 | ENSG00000172728 | *FUT10* | 8 | 33405912 | 33450206 | 44294 | 0.472 | 2.16e−07 |
| 242 | GIH | rs1545480 | ILMN_1727618 | ENSG00000156170 | *C8orf38* | 8 | 96141615 | 95977217 | 164398 | −0.376 | 5.01e−04 |
| 243 | MEX | rs1550186 | ILMN_1781001 | ENSG00000184557 | *SOCS3* | 17 | 73215285 | 73867753 | 652468 | 0.493 | 5.83e−04 |
| 244 | YRI | rs1551760 | ILMN_1722390 | ENSG00000104472 | *CHRAC1* | 8 | 140846769 | 141590586 | 743817 | 0.37 | 8.01e−05 |
| 245 | LWK | rs1551805 | ILMN_1722390 | ENSG00000104472 | *CHRAC1* | 8 | 140957737 | 141590586 | 632849 | 0.457 | 1.62e−05 |
| 246 | JPT | rs1579445 | ILMN_1794823 | ENSG00000188171 | *ZNF626* | 19 | 20591559 | 20636246 | 44687 | −0.472 | 7.57e−06 |
| 247 | GIH | rs1585804 | ILMN_1762071 | ENSG00000141219 | *C17orf80* | 17 | 69345596 | 68740953 | 604643 | 0.377 | 4.78e−04 |
| 248 | GIH | rs1620525 | ILMN_1727142 | ENSG00000104365 | *IKBKB* | 8 | 42984073 | 42247986 | 736087 | 0.361 | 8.53e−04 |
| 249 | CEU | rs1625919 | ILMN_1811433 | ENSG00000161016 | *RPL8* | 8 | 146036857 | 145988609 | 48248 | −0.366 | 8.96e−05 |
| 250 | MEX | rs1675262 | ILMN_1654943 | ENSG00000089685 | *BIRC5* | 17 | 74673240 | 73721872 | 951368 | −0.528 | 1.94e−04 |
| 251 | GIH | rs16880849 | ILMN_1725683 | ENSG00000172728 | *FUT10* | 8 | 33366080 | 33450206 | 84126 | 0.378 | 4.69e−04 |
| 252 | JPT | rs16880852 | ILMN_1725683 | ENSG00000172728 | *FUT10* | 8 | 33366227 | 33450206 | 83979 | −0.389 | 3.08e−04 |
| 253 | GIH | rs16880933 | ILMN_1725683 | ENSG00000172728 | *FUT10* | 8 | 33390878 | 33450206 | 59328 | −0.415 | 1.29e−04 |
| 254 | LWK | rs16880994 | ILMN_1725683 | ENSG00000172728 | *FUT10* | 8 | 33430477 | 33450206 | 19729 | −0.423 | 7.53e−05 |
| 255 | MKK | rs16889640 | ILMN_1805221 | ENSG00000147533 | *GOLGA7* | 8 | 40571715 | 41467238 | 895523 | 0.314 | 1.73e−04 |
| 256 | GIH | rs16889988 | ILMN_1805221 | ENSG00000147533 | *GOLGA7* | 8 | 40795932 | 41467238 | 671306 | −0.359 | 9.35e−04 |
| 257 | JPT | rs16890009 | ILMN_1675070 | ENSG00000147533 | *GOLGA7* | 8 | 40807645 | 41467238 | 659593 | −0.358 | 9.49e−04 |
| 258 | CEU | rs16911045 | ILMN_1722390 | ENSG00000104472 | *CHRAC1* | 8 | 140673825 | 141590586 | 916761 | −0.331 | 4.62e−04 |
| 259 | CHB | rs16916221 | ILMN_1775573 | ENSG00000156162 | *DPY19L4* | 8 | 94896836 | 95801327 | 904491 | −0.38 | 5.06e−04 |
| 260 | JPT | rs16916461 | ILMN_1714108 | ENSG00000164938 | *TP53INP1* | 8 | 95159961 | 96030770 | 870809 | −0.366 | 7.19e−04 |
| 261 | JPT | rs16916513 | ILMN_1714108 | ENSG00000164938 | *TP53INP1* | 8 | 95182419 | 96030770 | 848351 | −0.434 | 4.57e−05 |
| 262 | LWK | rs16916972 | ILMN_1714108 | ENSG00000164938 | *TP53INP1* | 8 | 95755390 | 96030770 | 275380 | 0.372 | 5.74e−04 |
| 263 | GIH | rs16917233 | ILMN_1727618 | ENSG00000156170 | *C8orf38* | 8 | 96114143 | 95977217 | 136926 | −0.4 | 1.96e−04 |
| 264 | GIH | rs16917360 | ILMN_1813635 | ENSG00000164944 | *KIAA1429* | 8 | 96196308 | 95634864 | 561444 | 0.376 | 5.04e−04 |
| 265 | GIH | rs16917761 | ILMN_1775573 | ENSG00000156162 | *DPY19L4* | 8 | 96567252 | 95801327 | 765925 | −0.372 | 6.20e−04 |
| 266 | CEU | rs16938859 | ILMN_1717420 | ENSG00000104343 | *UBE2W* | 8 | 75375900 | 74923937 | 451963 | 0.339 | 3.09e−04 |
| 267 | CHB | rs16943468 | ILMN_1687703 | ENSG00000108395 | *TRIM37* | 17 | 54799891 | 54539011 | 260880 | 0.409 | 1.63e−04 |
| 268 | CEU | rs16943973 | ILMN_1791106 | ENSG00000068097 | *HEATR6* | 17 | 55472211 | 55511074 | 38863 | −0.314 | 8.84e−04 |
| 269 | CEU | rs16943991 | ILMN_1791106 | ENSG00000068097 | *HEATR6* | 17 | 55476235 | 55511074 | 34839 | −0.314 | 8.84e−04 |
| 270 | CEU | rs16943994 | ILMN_1791106 | ENSG00000068097 | *HEATR6* | 17 | 55490984 | 55511074 | 20090 | −0.314 | 8.84e−04 |
| 271 | LWK | rs16944022 | ILMN_1791106 | ENSG00000068097 | *HEATR6* | 17 | 55517984 | 55511074 | 6910 | −0.424 | 7.26e−05 |
| 272 | CHB | rs16944295 | ILMN_1791106 | ENSG00000068097 | *HEATR6* | 17 | 55798087 | 55511074 | 287013 | −0.458 | 1.96e−05 |
| 273 | JPT | rs16970304 | ILMN_1781001 | ENSG00000184557 | *SOCS3* | 17 | 73084384 | 73867753 | 783369 | 0.386 | 3.48e−04 |
| 274 | MKK | rs16981320 | ILMN_1754923 | ENSG00000105085 | *MED26* | 19 | 16564437 | 16600015 | 35578 | −0.278 | 9.52e−04 |
| 275 | LWK | rs16995436 | ILMN_1752333 | ENSG00000127526 | *SLC35E1* | 19 | 16691424 | 16544193 | 147231 | 0.363 | 8.66e−04 |
| 276 | YRI | rs16995469 | ILMN_1702507 | ENSG00000130517 | *PGPEP1* | 19 | 17763208 | 18312408 | 549200 | −0.346 | 2.50e−04 |
| 277 | CHB | rs17088621 | ILMN_1714081 | ENSG00000008853 | *RHOBTB2* | 8 | 22699488 | 22913059 | 213571 | −0.378 | 5.48e−04 |
| 278 | YRI | rs17088737 | ILMN_1715969 | ENSG00000147454 | *SLC25A37* | 8 | 22817408 | 23442308 | 624900 | 0.317 | 8.19e−04 |
| 279 | CHB | rs17089022 | ILMN_1714081 | ENSG00000008853 | *RHOBTB2* | 8 | 23192620 | 22913059 | 279561 | 0.416 | 1.25e−04 |
| 280 | CHB | rs17089043 | ILMN_1714081 | ENSG00000008853 | *RHOBTB2* | 8 | 23222226 | 22913059 | 309167 | −0.386 | 4.02e−04 |
| 281 | CHB | rs17089055 | ILMN_1714081 | ENSG00000008853 | *RHOBTB2* | 8 | 23223799 | 22913059 | 310740 | 0.386 | 4.02e−04 |
| 282 | MEX | rs17210378 | ILMN_1707391 | ENSG00000166263 | *STXBP4* | 17 | 50679504 | 50401125 | 278379 | 0.488 | 7.83e−04 |
| 283 | CEU | rs1735166 | ILMN_1811433 | ENSG00000161016 | *RPL8* | 8 | 146035977 | 145988609 | 47368 | 0.385 | 3.63e−05 |
| 284 | CEU | rs1735169 | ILMN_1811433 | ENSG00000161016 | *RPL8* | 8 | 146037858 | 145988609 | 49249 | −0.385 | 3.63e−05 |
| 285 | CEU | rs1735189 | ILMN_1811433 | ENSG00000161016 | *RPL8* | 8 | 146016258 | 145988609 | 27649 | 0.361 | 1.24e−04 |
| 286 | CEU | rs1735192 | ILMN_1811433 | ENSG00000161016 | *RPL8* | 8 | 146016672 | 145988609 | 28063 | −0.385 | 3.63e−05 |
| 287 | CEU | rs1735404 | ILMN_1811433 | ENSG00000161016 | *RPL8* | 8 | 146042717 | 145988609 | 54108 | −0.357 | 1.41e−04 |
| 288 | CEU | rs1735405 | ILMN_1811433 | ENSG00000161016 | *RPL8* | 8 | 146041358 | 145988609 | 52749 | 0.375 | 5.86e−05 |
| 289 | CEU | rs1735412 | ILMN_1811433 | ENSG00000161016 | *RPL8* | 8 | 146031789 | 145988609 | 43180 | 0.385 | 3.63e−05 |
| 290 | CEU | rs1735430 | ILMN_1811433 | ENSG00000161016 | *RPL8* | 8 | 146022990 | 145988609 | 34381 | 0.388 | 3.58e−05 |
| 291 | CEU | rs1735433 | ILMN_1811433 | ENSG00000161016 | *RPL8* | 8 | 146021800 | 145988609 | 33191 | −0.385 | 3.63e−05 |
| 292 | MKK | rs17515835 | ILMN_1705871 | ENSG00000085788 | *DDHD2* | 8 | 38884099 | 38208264 | 675835 | −0.285 | 7.05e−04 |
| 293 | GIH | rs17562411 | ILMN_1805221 | ENSG00000147533 | *GOLGA7* | 8 | 40818998 | 41467238 | 648240 | −0.365 | 7.54e−04 |
| 294 | MKK | rs17607741 | ILMN_1676946 | ENSG00000070718 | *AP3M2* | 8 | 41932516 | 42129748 | 197232 | −0.277 | 9.99e−04 |
| 295 | CEU | rs17700954 | ILMN_1715969 | ENSG00000147454 | *SLC25A37* | 8 | 23503416 | 23442308 | 61108 | −0.324 | 5.92e−04 |
| 296 | MKK | rs17746436 | ILMN_1741841 | ENSG00000164944 | *KIAA1429* | 8 | 96522881 | 95634864 | 888017 | 0.278 | 9.57e−04 |
| 297 | MEX | rs17817797 | ILMN_1707391 | ENSG00000166263 | *STXBP4* | 17 | 50687496 | 50401125 | 286371 | 0.533 | 1.61e−04 |
| 298 | CEU | rs17842773 | ILMN_1791106 | ENSG00000068097 | *HEATR6* | 17 | 55438497 | 55511074 | 72577 | −0.345 | 2.36e−04 |
| 299 | MEX | rs1785631 | ILMN_1736689 | ENSG00000173599 | *PC* | 11 | 65849306 | 66482423 | 633117 | 0.545 | 1.07e−04 |
| 300 | MKK | rs181242 | ILMN_1687703 | ENSG00000108395 | *TRIM37* | 17 | 53558784 | 54539011 | 980227 | 0.281 | 8.56e−04 |
| 301 | MKK | rs181246 | ILMN_1687703 | ENSG00000108395 | *TRIM37* | 17 | 53561087 | 54539011 | 977924 | −0.283 | 7.79e−04 |
| 302 | MKK | rs1812642 | ILMN_1794823 | ENSG00000188171 | *ZNF626* | 19 | 20533429 | 20636246 | 102817 | −0.278 | 9.98e−04 |
| 303 | CHB | rs1823059 | ILMN_1656118 | ENSG00000178951 | *ZBTB7A* | 19 | 4066993 | 4017816 | 49177 | 0.37 | 7.91e−04 |
| 304 | CEU | rs1829189 | ILMN_1725683 | ENSG00000172728 | *FUT10* | 8 | 33469838 | 33450206 | 19632 | −0.455 | 8.25e−07 |
| 305 | GIH | rs1866844 | ILMN_1813635 | ENSG00000164944 | *KIAA1429* | 8 | 95600595 | 95634864 | 34269 | −0.365 | 7.47e−04 |
| 306 | MKK | rs1869440 | ILMN_1724611 | ENSG00000184428 | *TOP1MT* | 8 | 144673840 | 144488425 | 185415 | −0.278 | 9.58e−04 |
| 307 | JPT | rs187048 | ILMN_1669572 | ENSG00000070423 | *RNF126* | 19 | 880257 | 614227 | 266030 | 0.371 | 6.06e−04 |
| 308 | YRI | rs1871991 | ILMN_1740752 | ENSG00000158669 | *AGPAT6* | 8 | 41942695 | 41554876 | 387819 | −0.332 | 4.44e−04 |
| 309 | JPT | rs1877674 | ILMN_1715969 | ENSG00000147454 | *SLC25A37* | 8 | 22662237 | 23442308 | 780071 | 0.368 | 6.76e−04 |
| 310 | LWK | rs198444 | ILMN_1685676 | ENSG00000162222 | *TTC9C* | 11 | 61261744 | 62252160 | 990416 | 0.395 | 2.42e−04 |
| 311 | GIH | rs1985502 | ILMN_1708369 | ENSG00000127527 | *EPS15L1* | 19 | 15993558 | 16443768 | 450210 | 0.413 | 1.16e−04 |
| 312 | YRI | rs199219 | ILMN_1722390 | ENSG00000104472 | *CHRAC1* | 8 | 140795371 | 141590586 | 795215 | −0.334 | 4.04e−04 |
| 313 | CEU | rs1994448 | ILMN_1695404 | ENSG00000160932 | *LY6E* | 8 | 144585526 | 144171274 | 414252 | −0.318 | 7.95e−04 |
| 314 | CEU | rs2001635 | ILMN_1740240 | ENSG00000173599 | *PC* | 11 | 66618971 | 66482423 | 136548 | 0.324 | 5.93e−04 |
| 315 | YRI | rs2011883 | ILMN_1811433 | ENSG00000161016 | *RPL8* | 8 | 146233475 | 145988609 | 244866 | −0.313 | 9.58e−04 |
| 316 | LWK | rs2016792 | ILMN_1654943 | ENSG00000089685 | *BIRC5* | 17 | 73040909 | 73721872 | 680963 | −0.365 | 7.39e−04 |
| 317 | CEU | rs2037213 | ILMN_1725683 | ENSG00000172728 | *FUT10* | 8 | 33461560 | 33450206 | 11354 | 0.532 | 2.57e−09 |
| 318 | CEU | rs2043967 | ILMN_1725683 | ENSG00000172728 | *FUT10* | 8 | 33516574 | 33450206 | 66368 | −0.503 | 2.53e−08 |
| 319 | GIH | rs2048528 | ILMN_1806304 | ENSG00000197217 | *ENTPD4* | 8 | 23429625 | 23371115 | 58510 | −0.383 | 3.90e−04 |
| 320 | LWK | rs2060261 | ILMN_1708369 | ENSG00000127527 | *EPS15L1* | 19 | 15482180 | 16443768 | 961588 | 0.364 | 7.77e−04 |
| 321 | CEU | rs2070713 | ILMN_1676946 | ENSG00000070718 | *AP3M2* | 8 | 42164812 | 42129748 | 35064 | 0.327 | 5.28e−04 |
| 322 | MKK | rs2071213 | ILMN_1685676 | ENSG00000162222 | *TTC9C* | 11 | 61290586 | 62252160 | 961574 | −0.287 | 6.32e−04 |
| 323 | JPT | rs2071214 | ILMN_1654943 | ENSG00000089685 | *BIRC5* | 17 | 73731186 | 73721872 | 9314 | 0.403 | 1.78e−04 |
| 324 | YRI | rs2081890 | ILMN_1794823 | ENSG00000188171 | *ZNF626* | 19 | 20647438 | 20636246 | 11192 | 0.366 | 9.65e−05 |
| 325 | CEU | rs2087840 | ILMN_1791106 | ENSG00000068097 | *HEATR6* | 17 | 55511209 | 55511074 | 135 | −0.426 | 3.80e−06 |
| 326 | JPT | rs2112176 | ILMN_1702507 | ENSG00000130517 | *PGPEP1* | 19 | 18316679 | 18312408 | 4271 | 0.397 | 2.24e−04 |
| 327 | LWK | rs2115097 | ILMN_1794823 | ENSG00000188171 | *ZNF626* | 19 | 20682138 | 20636246 | 45892 | 0.375 | 5.61e−04 |
| 328 | YRI | rs2126128 | ILMN_1722390 | ENSG00000104472 | *CHRAC1* | 8 | 140857481 | 141590586 | 733105 | −0.336 | 3.80e−04 |
| 329 | YRI | rs2132864 | ILMN_1659801 | ENSG00000155097 | *ATP6V1C1* | 8 | 104155041 | 104102445 | 52596 | −0.357 | 1.47e−04 |
| 330 | CEU | rs2142182 | ILMN_1717420 | ENSG00000104343 | *UBE2W* | 8 | 75372632 | 74923937 | 448695 | −0.335 | 3.93e−04 |
| 331 | CHB | rs2159983 | ILMN_1654246 | ENSG00000077463 | *SIRT6* | 19 | 4137395 | 4133596 | 3799 | −0.469 | 1.13e−05 |
| 332 | GIH | rs216271 | ILMN_1686610 | ENSG00000011132 | *APBA3* | 19 | 2964616 | 3712673 | 748057 | 0.367 | 7.00e−04 |
| 333 | CEU | rs2235116 | ILMN_1715969 | ENSG00000147454 | *SLC25A37* | 8 | 23393355 | 23442308 | 48953 | 0.331 | 4.67e−04 |
| 334 | LWK | rs2238639 | ILMN_1673069 | ENSG00000142002 | *DPP9* | 19 | 5212230 | 4674875 | 537355 | 0.372 | 5.86e−04 |
| 335 | LWK | rs2238640 | ILMN_1673069 | ENSG00000142002 | *DPP9* | 19 | 5212506 | 4674875 | 537631 | −0.359 | 9.93e−04 |
| 336 | GIH | rs2239680 | ILMN_1710082 | ENSG00000089685 | *BIRC5* | 17 | 73731378 | 73721872 | 9506 | 0.365 | 7.37e−04 |
| 337 | LWK | rs2241260 | ILMN_1714081 | ENSG00000008853 | *RHOBTB2* | 8 | 22931930 | 22913059 | 18871 | 0.381 | 4.16e−04 |
| 338 | JPT | rs2242196 | ILMN_1768273 | ENSG00000104356 | *POP1* | 8 | 99187830 | 99199244 | 11414 | −0.388 | 3.17e−04 |
| 339 | MKK | rs2242462 | ILMN_1776088 | ENSG00000109065 | *NAT9* | 17 | 70029419 | 70284065 | 254646 | −0.28 | 8.75e−04 |
| 340 | CEU | rs2242650 | ILMN_1811433 | ENSG00000161016 | *RPL8* | 8 | 146037222 | 145988609 | 48613 | −0.385 | 3.63e−05 |
| 341 | CEU | rs2242651 | ILMN_1811433 | ENSG00000161016 | *RPL8* | 8 | 146037312 | 145988609 | 48703 | −0.385 | 3.63e−05 |
| 342 | MEX | rs2249098 | ILMN_1666022 | ENSG00000173530 | *TNFRSF10D* | 8 | 22456043 | 23077485 | 621442 | −0.483 | 7.85e−04 |
| 343 | LWK | rs2253138 | ILMN_1778673 | ENSG00000147533 | *GOLGA7* | 8 | 40538301 | 41467238 | 928937 | 0.397 | 2.22e−04 |
| 344 | LWK | rs225579 | ILMN_1762071 | ENSG00000141219 | *C17orf80* | 17 | 69206917 | 68740953 | 465964 | 0.373 | 7.63e−04 |
| 345 | CEU | rs2256154 | ILMN_1740240 | ENSG00000173599 | *PC* | 11 | 66847147 | 66482423 | 364724 | −0.366 | 8.99e−05 |
| 346 | YRI | rs2272635 | ILMN_1659651 | ENSG00000184428 | *TOP1MT* | 8 | 144471440 | 144488425 | 16985 | −0.375 | 6.44e−05 |
| 347 | GIH | rs2272640 | ILMN_1806304 | ENSG00000197217 | *ENTPD4* | 8 | 23358109 | 23371115 | 13006 | 0.363 | 7.96e−04 |
| 348 | MEX | rs2279863 | ILMN_1736689 | ENSG00000173599 | *PC* | 11 | 66004272 | 66482423 | 478151 | 0.491 | 7.10e−04 |
| 349 | MKK | rs2280838 | ILMN_1706386 | ENSG00000147804 | *SLC39A4* | 8 | 145612810 | 145613081 | 271 | 0.283 | 7.57e−04 |
| 350 | CEU | rs2280861 | ILMN_1715969 | ENSG00000147454 | *SLC25A37* | 8 | 23460730 | 23442308 | 18422 | 0.313 | 9.20e−04 |
| 351 | YRI | rs2288464 | ILMN_1798083 | ENSG00000085872 | *CHERP* | 19 | 17278252 | 16514263 | 763989 | −0.352 | 1.89e−04 |
| 352 | MKK | rs2289071 | ILMN_1724611 | ENSG00000184428 | *TOP1MT* | 8 | 143848424 | 144488425 | 640001 | 0.283 | 7.59e−04 |
| 353 | CEU | rs2292741 | ILMN_1789138 | ENSG00000169499 | *PLEKHA2* | 8 | 38815153 | 38877986 | 62833 | 0.324 | 5.93e−04 |
| 354 | LWK | rs2293939 | ILMN_1722390 | ENSG00000104472 | *CHRAC1* | 8 | 141620589 | 141590586 | 30003 | −0.393 | 2.65e−04 |
| 355 | CEU | rs2294117 | ILMN_1696752 | ENSG00000014164 | *ZC3H3* | 8 | 144591290 | 144694746 | 103456 | −0.372 | 6.75e−05 |
| 356 | CEU | rs2294120 | ILMN_1811433 | ENSG00000161016 | *RPL8* | 8 | 145974371 | 145988609 | 14238 | 0.599 | 9.81e−12 |
| 357 | CHB | rs2294126 | ILMN_1714081 | ENSG00000008853 | *RHOBTB2* | 8 | 23223380 | 22913059 | 310321 | 0.386 | 4.02e−04 |
| 358 | CEU | rs2302202 | ILMN_1791106 | ENSG00000068097 | *HEATR6* | 17 | 55480242 | 55511074 | 30832 | −0.314 | 8.84e−04 |
| 359 | CEU | rs2304748 | ILMN_1725683 | ENSG00000172728 | *FUT10* | 8 | 33489486 | 33450206 | 39280 | 0.543 | 1.10e−09 |
| 360 | MKK | rs2304875 | ILMN_1729816 | ENSG00000078668 | *VDAC3* | 8 | 41694596 | 42368547 | 673951 | −0.281 | 8.61e−04 |
| 361 | YRI | rs2306383 | ILMN_1706386 | ENSG00000147804 | *SLC39A4* | 8 | 145636324 | 145613081 | 23243 | −0.318 | 7.92e−04 |
| 362 | JPT | rs2313167 | ILMN_1786345 | ENSG00000158941 | *KIAA1967* | 8 | 22618699 | 22518202 | 100497 | 0.371 | 6.10e−04 |
| 363 | JPT | rs2340015 | ILMN_1714108 | ENSG00000164938 | *TP53INP1* | 8 | 95184445 | 96030770 | 846325 | −0.372 | 5.69e−04 |
| 364 | CEU | rs2348613 | ILMN_1725683 | ENSG00000172728 | *FUT10* | 8 | 33414066 | 33450206 | 36140 | 0.48 | 1.51e−07 |
| 365 | GIH | rs2376493 | ILMN_1729816 | ENSG00000078668 | *VDAC3* | 8 | 42805356 | 42368547 | 436809 | 0.389 | 3.02e−04 |
| 366 | GIH | rs2377402 | ILMN_1781001 | ENSG00000184557 | *SOCS3* | 17 | 74843041 | 73867753 | 975288 | 0.403 | 1.73e−04 |
| 367 | CHB | rs237954 | ILMN_1791106 | ENSG00000068097 | *HEATR6* | 17 | 55698601 | 55511074 | 187527 | −0.414 | 1.34e−04 |
| 368 | CHB | rs237956 | ILMN_1791106 | ENSG00000068097 | *HEATR6* | 17 | 55695726 | 55511074 | 184652 | 0.414 | 1.34e−04 |
| 369 | CHB | rs237967 | ILMN_1791106 | ENSG00000068097 | *HEATR6* | 17 | 55686029 | 55511074 | 174955 | −0.414 | 1.34e−04 |
| 370 | MKK | rs2382954 | ILMN_1796508 | ENSG00000184428 | *TOP1MT* | 8 | 144684227 | 144488425 | 195802 | 0.279 | 9.36e−04 |
| 371 | GIH | rs2404332 | ILMN_1714081 | ENSG00000008853 | *RHOBTB2* | 8 | 22244275 | 22913059 | 668784 | 0.357 | 9.83e−04 |
| 372 | GIH | rs2404336 | ILMN_1699265 | ENSG00000120889 | *TNFRSF10B* | 8 | 22259379 | 22982637 | 723258 | −0.373 | 5.94e−04 |
| 373 | MEX | rs2430802 | ILMN_1804789 | ENSG00000158941 | *KIAA1967* | 8 | 22893066 | 22518202 | 374864 | 0.513 | 3.13e−04 |
| 374 | CEU | rs2430822 | ILMN_1722390 | ENSG00000104472 | *CHRAC1* | 8 | 140679423 | 141590586 | 911163 | −0.394 | 2.25e−05 |
| 375 | GIH | rs2431801 | ILMN_1754923 | ENSG00000105085 | *MED26* | 19 | 16766527 | 16600015 | 166512 | −0.393 | 2.55e−04 |
| 376 | GIH | rs2438201 | ILMN_1754303 | ENSG00000156482 | *RPL30* | 8 | 98842647 | 99129034 | 286387 | −0.371 | 6.04e−04 |
| 377 | MEX | rs2443500 | ILMN_1666022 | ENSG00000173530 | *TNFRSF10D* | 8 | 22408287 | 23077485 | 669198 | 0.539 | 1.32e−04 |
| 378 | MEX | rs2443502 | ILMN_1666022 | ENSG00000173530 | *TNFRSF10D* | 8 | 22396444 | 23077485 | 681041 | −0.483 | 7.85e−04 |
| 379 | MEX | rs244391 | ILMN_1707391 | ENSG00000166263 | *STXBP4* | 17 | 50666660 | 50401125 | 265535 | 0.519 | 2.63e−04 |
| 380 | MEX | rs244400 | ILMN_1707391 | ENSG00000166263 | *STXBP4* | 17 | 50674595 | 50401125 | 273470 | −0.49 | 6.35e−04 |
| 381 | MEX | rs244401 | ILMN_1707391 | ENSG00000166263 | *STXBP4* | 17 | 50675059 | 50401125 | 273934 | 0.49 | 6.35e−04 |
| 382 | YRI | rs2444889 | ILMN_1768273 | ENSG00000104356 | *POP1* | 8 | 98977374 | 99199244 | 221870 | −0.331 | 5.55e−04 |
| 383 | MEX | rs2449340 | ILMN_1666022 | ENSG00000173530 | *TNFRSF10D* | 8 | 22445845 | 23077485 | 631640 | −0.483 | 7.85e−04 |
| 384 | MKK | rs2450749 | ILMN_1724611 | ENSG00000184428 | *TOP1MT* | 8 | 144505931 | 144488425 | 17506 | −0.279 | 9.23e−04 |
| 385 | MKK | rs2450758 | ILMN_1659651 | ENSG00000184428 | *TOP1MT* | 8 | 144509422 | 144488425 | 20997 | 0.402 | 9.99e−07 |
| 386 | MKK | rs2450763 | ILMN_1659651 | ENSG00000184428 | *TOP1MT* | 8 | 144519768 | 144488425 | 31343 | −0.43 | 1.42e−07 |
| 387 | MKK | rs2450764 | ILMN_1724611 | ENSG00000184428 | *TOP1MT* | 8 | 144520567 | 144488425 | 32142 | 0.285 | 6.99e−04 |
| 388 | MKK | rs2450772 | ILMN_1659651 | ENSG00000184428 | *TOP1MT* | 8 | 144484791 | 144488425 | 3634 | −0.467 | 7.78e−09 |
| 389 | LWK | rs2450776 | ILMN_1659651 | ENSG00000184428 | *TOP1MT* | 8 | 144488815 | 144488425 | 390 | −0.419 | 8.86e−05 |
| 390 | YRI | rs2453997 | ILMN_1659801 | ENSG00000155097 | *ATP6V1C1* | 8 | 104154620 | 104102445 | 52175 | 0.359 | 1.35e−04 |
| 391 | LWK | rs2457429 | ILMN_1666022 | ENSG00000173530 | *TNFRSF10D* | 8 | 22843831 | 23077485 | 233654 | −0.373 | 5.60e−04 |
| 392 | CEU | rs2459516 | ILMN_1725683 | ENSG00000172728 | *FUT10* | 8 | 33403549 | 33450206 | 46657 | 0.469 | 3.12e−07 |
| 393 | MEX | rs2461491 | ILMN_1666022 | ENSG00000173530 | *TNFRSF10D* | 8 | 22417197 | 23077485 | 660288 | 0.475 | 9.83e−04 |
| 394 | YRI | rs2466215 | ILMN_1804789 | ENSG00000158941 | *KIAA1967* | 8 | 22831364 | 22518202 | 313162 | −0.339 | 3.58e−04 |
| 395 | MEX | rs2466236 | ILMN_1804789 | ENSG00000158941 | *KIAA1967* | 8 | 22892733 | 22518202 | 374531 | 0.496 | 6.10e−04 |
| 396 | MKK | rs2467898 | ILMN_1659651 | ENSG00000184428 | *TOP1MT* | 8 | 144524691 | 144488425 | 36266 | −0.332 | 6.97e−05 |
| 397 | LWK | rs2467933 | ILMN_1659651 | ENSG00000184428 | *TOP1MT* | 8 | 144499560 | 144488425 | 11135 | 0.443 | 3.06e−05 |
| 398 | GIH | rs2467938 | ILMN_1782751 | ENSG00000181638 | *GLI4* | 8 | 144517660 | 144400484 | 117176 | 0.37 | 6.30e−04 |
| 399 | MKK | rs2467950 | ILMN_1796508 | ENSG00000184428 | *TOP1MT* | 8 | 144520566 | 144488425 | 32141 | 0.327 | 1.09e−04 |
| 400 | MEX | rs2469747 | ILMN_1666022 | ENSG00000173530 | *TNFRSF10D* | 8 | 22411258 | 23077485 | 666227 | −0.483 | 7.87e−04 |
| 401 | MEX | rs2469749 | ILMN_1666022 | ENSG00000173530 | *TNFRSF10D* | 8 | 22413543 | 23077485 | 663942 | −0.557 | 7.00e−05 |
| 402 | MEX | rs2469760 | ILMN_1666022 | ENSG00000173530 | *TNFRSF10D* | 8 | 22421890 | 23077485 | 655595 | 0.515 | 4.12e−04 |
| 403 | MEX | rs2469776 | ILMN_1666022 | ENSG00000173530 | *TNFRSF10D* | 8 | 22459396 | 23077485 | 618089 | 0.507 | 3.83e−04 |
| 404 | LWK | rs2471094 | ILMN_1722390 | ENSG00000104472 | *CHRAC1* | 8 | 140704528 | 141590586 | 886058 | 0.408 | 1.41e−04 |
| 405 | JPT | rs2513759 | ILMN_1714108 | ENSG00000164938 | *TP53INP1* | 8 | 95173673 | 96030770 | 857097 | −0.364 | 7.67e−04 |
| 406 | JPT | rs2514344 | ILMN_1754303 | ENSG00000156482 | *RPL30* | 8 | 99170315 | 99129034 | 41281 | −0.36 | 8.95e−04 |
| 407 | GIH | rs2547108 | ILMN_1754923 | ENSG00000105085 | *MED26* | 19 | 16760731 | 16600015 | 160716 | −0.394 | 2.48e−04 |
| 408 | CHB | rs2554399 | ILMN_1813635 | ENSG00000164944 | *KIAA1429* | 8 | 95769864 | 95634864 | 135000 | −0.373 | 6.66e−04 |
| 409 | CEU | rs2581896 | ILMN_1725683 | ENSG00000172728 | *FUT10* | 8 | 33490527 | 33450206 | 40321 | −0.516 | 9.02e−09 |
| 410 | CEU | rs2581898 | ILMN_1725683 | ENSG00000172728 | *FUT10* | 8 | 33490692 | 33450206 | 40486 | −0.483 | 1.02e−07 |
| 411 | LWK | rs2599658 | ILMN_1807501 | ENSG00000147536 | *GINS4* | 8 | 40620325 | 41505925 | 885600 | −0.375 | 5.15e−04 |
| 412 | LWK | rs2599662 | ILMN_1807501 | ENSG00000147536 | *GINS4* | 8 | 40634761 | 41505925 | 871164 | −0.387 | 3.25e−04 |
| 413 | YRI | rs2599707 | ILMN_1714108 | ENSG00000164938 | *TP53INP1* | 8 | 96146551 | 96030770 | 115781 | −0.332 | 4.97e−04 |
| 414 | GIH | rs2608725 | ILMN_1754923 | ENSG00000105085 | *MED26* | 19 | 16775952 | 16600015 | 175937 | −0.368 | 6.79e−04 |
| 415 | GIH | rs2608738 | ILMN_1754923 | ENSG00000105085 | *MED26* | 19 | 16760865 | 16600015 | 160850 | −0.39 | 2.88e−04 |
| 416 | MKK | rs2628305 | ILMN_1707391 | ENSG00000166263 | *STXBP4* | 17 | 50495426 | 50401125 | 94301 | 0.294 | 4.68e−04 |
| 417 | GIH | rs2661694 | ILMN_1710082 | ENSG00000089685 | *BIRC5* | 17 | 73732603 | 73721872 | 10731 | 0.365 | 7.37e−04 |
| 418 | MKK | rs2666002 | ILMN_1802190 | ENSG00000129654 | *FOXJ1* | 17 | 71639931 | 71648966 | 9035 | −0.28 | 8.91e−04 |
| 419 | CEU | rs2676403 | ILMN_1725683 | ENSG00000172728 | *FUT10* | 8 | 33467542 | 33450206 | 17336 | −0.523 | 8.94e−09 |
| 420 | CEU | rs2676415 | ILMN_1725683 | ENSG00000172728 | *FUT10* | 8 | 33450154 | 33450206 | 52 | 0.352 | 2.15e−04 |
| 421 | CEU | rs2676419 | ILMN_1725683 | ENSG00000172728 | *FUT10* | 8 | 33475428 | 33450206 | 25222 | −0.514 | 1.49e−08 |
| 422 | GIH | rs2676434 | ILMN_1725683 | ENSG00000172728 | *FUT10* | 8 | 33395335 | 33450206 | 54871 | −0.397 | 2.22e−04 |
| 423 | GIH | rs2678823 | ILMN_1727618 | ENSG00000156170 | *C8orf38* | 8 | 96115270 | 95977217 | 138053 | −0.391 | 2.82e−04 |
| 424 | GIH | rs2678829 | ILMN_1727618 | ENSG00000156170 | *C8orf38* | 8 | 96097356 | 95977217 | 120139 | 0.391 | 2.82e−04 |
| 425 | CEU | rs2721173 | ILMN_1811433 | ENSG00000161016 | *RPL8* | 8 | 145715237 | 145988609 | 273372 | 0.338 | 3.21e−04 |
| 426 | CEU | rs2721195 | ILMN_1811433 | ENSG00000161016 | *RPL8* | 8 | 145647819 | 145988609 | 340790 | 0.337 | 3.62e−04 |
| 427 | CEU | rs2721197 | ILMN_1811433 | ENSG00000161016 | *RPL8* | 8 | 145698513 | 145988609 | 290096 | 0.35 | 2.20e−04 |
| 428 | CEU | rs2722469 | ILMN_1811433 | ENSG00000161016 | *RPL8* | 8 | 145926351 | 145988609 | 62258 | 0.424 | 4.43e−06 |
| 429 | CEU | rs2722495 | ILMN_1811433 | ENSG00000161016 | *RPL8* | 8 | 145942473 | 145988609 | 46136 | −0.607 | 2.63e−12 |
| 430 | CEU | rs2727229 | ILMN_1811433 | ENSG00000161016 | *RPL8* | 8 | 146041300 | 145988609 | 52691 | −0.375 | 5.86e−05 |
| 431 | CEU | rs2727237 | ILMN_1811433 | ENSG00000161016 | *RPL8* | 8 | 146022007 | 145988609 | 33398 | −0.385 | 3.63e−05 |
| 432 | MKK | rs2727252 | ILMN_1811433 | ENSG00000161016 | *RPL8* | 8 | 145904828 | 145988609 | 83781 | 0.289 | 6.29e−04 |
| 433 | CEU | rs2730059 | ILMN_1811433 | ENSG00000161016 | *RPL8* | 8 | 145952565 | 145988609 | 36044 | −0.576 | 5.61e−11 |
| 434 | MKK | rs2730070 | ILMN_1811433 | ENSG00000161016 | *RPL8* | 8 | 145912613 | 145988609 | 75996 | −0.286 | 6.78e−04 |
| 435 | CEU | rs2732259 | ILMN_1725683 | ENSG00000172728 | *FUT10* | 8 | 33411574 | 33450206 | 38632 | −0.486 | 8.34e−08 |
| 436 | CEU | rs2732274 | ILMN_1725683 | ENSG00000172728 | *FUT10* | 8 | 33467449 | 33450206 | 17243 | 0.462 | 4.75e−07 |
| 437 | YRI | rs2732286 | ILMN_1725683 | ENSG00000172728 | *FUT10* | 8 | 33424099 | 33450206 | 26107 | 0.374 | 6.82e−05 |
| 438 | YRI | rs2732303 | ILMN_1725683 | ENSG00000172728 | *FUT10* | 8 | 33422558 | 33450206 | 27648 | 0.374 | 6.82e−05 |
| 439 | CEU | rs2732315 | ILMN_1725683 | ENSG00000172728 | *FUT10* | 8 | 33432871 | 33450206 | 17335 | 0.479 | 1.57e−07 |
| 440 | CEU | rs2732316 | ILMN_1725683 | ENSG00000172728 | *FUT10* | 8 | 33433647 | 33450206 | 16559 | 0.444 | 1.90e−06 |
| 441 | JPT | rs274815 | ILMN_1794823 | ENSG00000188171 | *ZNF626* | 19 | 20625937 | 20636246 | 10309 | 0.426 | 6.46e−05 |
| 442 | MKK | rs28475718 | ILMN_1764721 | ENSG00000161016 | *RPL8* | 8 | 145178168 | 145988609 | 810441 | −0.303 | 3.09e−04 |
| 443 | MKK | rs28483871 | ILMN_1764721 | ENSG00000161016 | *RPL8* | 8 | 145173369 | 145988609 | 815240 | −0.303 | 3.09e−04 |
| 444 | MKK | rs2872716 | ILMN_1666022 | ENSG00000173530 | *TNFRSF10D* | 8 | 23454378 | 23077485 | 376893 | 0.29 | 5.51e−04 |
| 445 | GIH | rs2885321 | ILMN_1754923 | ENSG00000105085 | *MED26* | 19 | 16768371 | 16600015 | 168356 | 0.366 | 7.22e−04 |
| 446 | LWK | rs2889 | ILMN_1714081 | ENSG00000008853 | *RHOBTB2* | 8 | 22931854 | 22913059 | 18795 | 0.381 | 4.16e−04 |
| 447 | GIH | rs2890827 | ILMN_1813635 | ENSG00000164944 | *KIAA1429* | 8 | 95607644 | 95634864 | 27220 | −0.363 | 7.95e−04 |
| 448 | YRI | rs2892452 | ILMN_1663257 | ENSG00000155097 | *ATP6V1C1* | 8 | 103600977 | 104102445 | 501468 | 0.319 | 8.99e−04 |
| 449 | MKK | rs2907633 | ILMN_1707391 | ENSG00000166263 | *STXBP4* | 17 | 50229366 | 50401125 | 171759 | 0.288 | 6.13e−04 |
| 450 | MKK | rs2907647 | ILMN_1707391 | ENSG00000166263 | *STXBP4* | 17 | 50242780 | 50401125 | 158345 | 0.288 | 6.13e−04 |
| 451 | CEU | rs2928665 | ILMN_1699473 | ENSG00000158941 | *KIAA1967* | 8 | 23476388 | 22518202 | 958186 | 0.319 | 7.55e−04 |
| 452 | GIH | rs2931710 | ILMN_1782751 | ENSG00000181638 | *GLI4* | 8 | 144512231 | 144400484 | 111747 | 0.37 | 6.30e−04 |
| 453 | MKK | rs2942219 | ILMN_1715969 | ENSG00000147454 | *SLC25A37* | 8 | 23495253 | 23442308 | 52945 | −0.283 | 8.16e−04 |
| 454 | CEU | rs2953854 | ILMN_1811433 | ENSG00000161016 | *RPL8* | 8 | 145961284 | 145988609 | 27325 | −0.592 | 1.19e−11 |
| 455 | CEU | rs2953857 | ILMN_1811433 | ENSG00000161016 | *RPL8* | 8 | 145971225 | 145988609 | 17384 | −0.592 | 1.19e−11 |
| 456 | JPT | rs2953874 | ILMN_1811433 | ENSG00000161016 | *RPL8* | 8 | 146100072 | 145988609 | 111463 | 0.389 | 3.02e−04 |
| 457 | CEU | rs2954660 | ILMN_1811433 | ENSG00000161016 | *RPL8* | 8 | 145994727 | 145988609 | 6118 | −0.316 | 9.70e−04 |
| 458 | MKK | rs2954675 | ILMN_1811433 | ENSG00000161016 | *RPL8* | 8 | 146041390 | 145988609 | 52781 | −0.288 | 6.24e−04 |
| 459 | CEU | rs2955195 | ILMN_1811433 | ENSG00000161016 | *RPL8* | 8 | 145959996 | 145988609 | 28613 | −0.604 | 3.66e−12 |
| 460 | CEU | rs2955202 | ILMN_1811433 | ENSG00000161016 | *RPL8* | 8 | 145970933 | 145988609 | 17676 | −0.591 | 1.37e−11 |
| 461 | CEU | rs2955203 | ILMN_1811433 | ENSG00000161016 | *RPL8* | 8 | 145973393 | 145988609 | 15216 | −0.319 | 7.12e−04 |
| 462 | CEU | rs2956173 | ILMN_1811433 | ENSG00000161016 | *RPL8* | 8 | 145897441 | 145988609 | 91168 | 0.386 | 3.32e−05 |
| 463 | CEU | rs2958482 | ILMN_1811433 | ENSG00000161016 | *RPL8* | 8 | 145955419 | 145988609 | 33190 | 0.574 | 8.16e−11 |
| 464 | CEU | rs2958483 | ILMN_1811433 | ENSG00000161016 | *RPL8* | 8 | 145957852 | 145988609 | 30757 | −0.607 | 2.63e−12 |
| 465 | CEU | rs2958492 | ILMN_1811433 | ENSG00000161016 | *RPL8* | 8 | 145889935 | 145988609 | 98674 | 0.343 | 2.66e−04 |
| 466 | CEU | rs2958516 | ILMN_1811433 | ENSG00000161016 | *RPL8* | 8 | 145989530 | 145988609 | 921 | −0.601 | 4.78e−12 |
| 467 | CEU | rs2958522 | ILMN_1811433 | ENSG00000161016 | *RPL8* | 8 | 145995756 | 145988609 | 7147 | −0.371 | 6.98e−05 |
| 468 | MEX | rs2975212 | ILMN_1704873 | ENSG00000154582 | *TCEB1* | 8 | 75404977 | 75046956 | 358021 | 0.552 | 8.50e−05 |
| 469 | JPT | rs2976464 | ILMN_1811433 | ENSG00000161016 | *RPL8* | 8 | 146099144 | 145988609 | 110535 | 0.439 | 3.62e−05 |
| 470 | CEU | rs2976602 | ILMN_1811433 | ENSG00000161016 | *RPL8* | 8 | 145815920 | 145988609 | 172689 | 0.364 | 9.96e−05 |
| 471 | CHB | rs2978388 | ILMN_1706386 | ENSG00000147804 | *SLC39A4* | 8 | 146124805 | 145613081 | 511724 | 0.366 | 8.47e−04 |
| 472 | MKK | rs2980268 | ILMN_1659651 | ENSG00000184428 | *TOP1MT* | 8 | 144491520 | 144488425 | 3095 | 0.5 | 4.16e−10 |
| 473 | MEX | rs2981269 | ILMN_1704873 | ENSG00000154582 | *TCEB1* | 8 | 75408539 | 75046956 | 361583 | 0.519 | 3.67e−04 |
| 474 | LWK | rs306947 | ILMN_1722390 | ENSG00000104472 | *CHRAC1* | 8 | 142085967 | 141590586 | 495381 | 0.411 | 1.51e−04 |
| 475 | CEU | rs3098657 | ILMN_1725683 | ENSG00000172728 | *FUT10* | 8 | 33489536 | 33450206 | 39330 | −0.483 | 1.02e−07 |
| 476 | GIH | rs3098705 | ILMN_1813635 | ENSG00000164944 | *KIAA1429* | 8 | 95585684 | 95634864 | 49180 | −0.363 | 7.95e−04 |
| 477 | GIH | rs3099418 | ILMN_1813635 | ENSG00000164944 | *KIAA1429* | 8 | 95623605 | 95634864 | 11259 | −0.363 | 7.95e−04 |
| 478 | GIH | rs3102494 | ILMN_1775573 | ENSG00000156162 | *DPY19L4* | 8 | 96739101 | 95801327 | 937774 | 0.396 | 2.34e−04 |
| 479 | GIH | rs3102495 | ILMN_1775573 | ENSG00000156162 | *DPY19L4* | 8 | 96738723 | 95801327 | 937396 | 0.396 | 2.34e−04 |
| 480 | GIH | rs3102499 | ILMN_1775573 | ENSG00000156162 | *DPY19L4* | 8 | 96736589 | 95801327 | 935262 | −0.396 | 2.34e−04 |
| 481 | GIH | rs3102854 | ILMN_1813635 | ENSG00000164944 | *KIAA1429* | 8 | 95567165 | 95634864 | 67699 | 0.365 | 7.47e−04 |
| 482 | GIH | rs3104888 | ILMN_1775573 | ENSG00000156162 | *DPY19L4* | 8 | 96690344 | 95801327 | 889017 | −0.406 | 1.57e−04 |
| 483 | GIH | rs3104961 | ILMN_1775573 | ENSG00000156162 | *DPY19L4* | 8 | 96668742 | 95801327 | 867415 | −0.399 | 2.08e−04 |
| 484 | GIH | rs3104969 | ILMN_1775573 | ENSG00000156162 | *DPY19L4* | 8 | 96662151 | 95801327 | 860824 | −0.427 | 6.42e−05 |
| 485 | GIH | rs3104993 | ILMN_1775573 | ENSG00000156162 | *DPY19L4* | 8 | 96641338 | 95801327 | 840011 | 0.427 | 6.42e−05 |
| 486 | GIH | rs3104996 | ILMN_1775573 | ENSG00000156162 | *DPY19L4* | 8 | 96632981 | 95801327 | 831654 | 0.427 | 6.42e−05 |
| 487 | LWK | rs3115924 | ILMN_1813635 | ENSG00000164944 | *KIAA1429* | 8 | 95026123 | 95634864 | 608741 | 0.373 | 5.48e−04 |
| 488 | LWK | rs3115940 | ILMN_1813635 | ENSG00000164944 | *KIAA1429* | 8 | 95040067 | 95634864 | 594797 | 0.395 | 2.84e−04 |
| 489 | GIH | rs311621 | ILMN_1654246 | ENSG00000077463 | *SIRT6* | 19 | 3149777 | 4133596 | 983819 | −0.391 | 2.77e−04 |
| 490 | GIH | rs3133659 | ILMN_1813635 | ENSG00000164944 | *KIAA1429* | 8 | 95634938 | 95634864 | 74 | 0.363 | 7.95e−04 |
| 491 | LWK | rs3134010 | ILMN_1813635 | ENSG00000164944 | *KIAA1429* | 8 | 95042388 | 95634864 | 592476 | −0.366 | 7.10e−04 |
| 492 | GIH | rs3134190 | ILMN_1775573 | ENSG00000156162 | *DPY19L4* | 8 | 96669266 | 95801327 | 867939 | −0.399 | 2.08e−04 |
| 493 | GIH | rs3134191 | ILMN_1775573 | ENSG00000156162 | *DPY19L4* | 8 | 96737024 | 95801327 | 935697 | −0.396 | 2.34e−04 |
| 494 | GIH | rs3134211 | ILMN_1775573 | ENSG00000156162 | *DPY19L4* | 8 | 96694726 | 95801327 | 893399 | −0.391 | 2.78e−04 |
| 495 | GIH | rs3134236 | ILMN_1775573 | ENSG00000156162 | *DPY19L4* | 8 | 96679147 | 95801327 | 877820 | −0.399 | 2.08e−04 |
| 496 | CEU | rs313776 | ILMN_1752423 | ENSG00000126934 | *MAP2K2* | 19 | 3266718 | 4075126 | 808408 | −0.335 | 3.76e−04 |
| 497 | CHB | rs3214050 | ILMN_1741841 | ENSG00000164944 | *KIAA1429* | 8 | 95255558 | 95634864 | 379306 | 0.362 | 9.85e−04 |
| 498 | MKK | rs34303577 | ILMN_1724611 | ENSG00000184428 | *TOP1MT* | 8 | 144991037 | 144488425 | 502612 | −0.291 | 5.32e−04 |
| 499 | CHB | rs345184 | ILMN_1791106 | ENSG00000068097 | *HEATR6* | 17 | 55594073 | 55511074 | 82999 | −0.414 | 1.34e−04 |
| 500 | CHB | rs345186 | ILMN_1791106 | ENSG00000068097 | *HEATR6* | 17 | 55590125 | 55511074 | 79051 | 0.37 | 7.32e−04 |
| 501 | CEU | rs345187 | ILMN_1791106 | ENSG00000068097 | *HEATR6* | 17 | 55588298 | 55511074 | 77224 | 0.316 | 8.05e−04 |
| 502 | CHB | rs350844 | ILMN_1654246 | ENSG00000077463 | *SIRT6* | 19 | 4128051 | 4133596 | 5545 | −0.503 | 2.31e−06 |
| 503 | CHB | rs350852 | ILMN_1654246 | ENSG00000077463 | *SIRT6* | 19 | 4120998 | 4133596 | 12598 | 0.405 | 1.93e−04 |
| 504 | MKK | rs350879 | ILMN_1673069 | ENSG00000142002 | *DPP9* | 19 | 4104659 | 4674875 | 570216 | 0.28 | 8.97e−04 |
| 505 | MEX | rs350890 | ILMN_1654246 | ENSG00000077463 | *SIRT6* | 19 | 4068705 | 4133596 | 64891 | −0.554 | 7.99e−05 |
| 506 | MEX | rs350891 | ILMN_1654246 | ENSG00000077463 | *SIRT6* | 19 | 4068290 | 4133596 | 65306 | −0.554 | 7.99e−05 |
| 507 | MEX | rs350892 | ILMN_1654246 | ENSG00000077463 | *SIRT6* | 19 | 4067652 | 4133596 | 65944 | 0.554 | 7.99e−05 |
| 508 | CHB | rs352493 | ILMN_1654246 | ENSG00000077463 | *SIRT6* | 19 | 4131836 | 4133596 | 1760 | 0.405 | 1.96e−04 |
| 509 | CEU | rs35770914 | ILMN_1811433 | ENSG00000161016 | *RPL8* | 8 | 145701138 | 145988609 | 287471 | 0.326 | 5.32e−04 |
| 510 | MKK | rs369051 | ILMN_1659651 | ENSG00000184428 | *TOP1MT* | 8 | 144619949 | 144488425 | 131524 | −0.347 | 3.47e−05 |
| 511 | CEU | rs3735950 | ILMN_1725683 | ENSG00000172728 | *FUT10* | 8 | 33535731 | 33450206 | 85525 | 0.472 | 2.24e−07 |
| 512 | CEU | rs3735951 | ILMN_1725683 | ENSG00000172728 | *FUT10* | 8 | 33535764 | 33450206 | 85558 | −0.495 | 4.44e−08 |
| 513 | CHB | rs3736022 | ILMN_1786345 | ENSG00000158941 | *KIAA1967* | 8 | 23171591 | 22518202 | 653389 | −0.394 | 2.99e−04 |
| 514 | LWK | rs3736025 | ILMN_1786345 | ENSG00000158941 | *KIAA1967* | 8 | 23168607 | 22518202 | 650405 | 0.42 | 8.60e−05 |
| 515 | JPT | rs3739253 | ILMN_1705871 | ENSG00000085788 | *DDHD2* | 8 | 38237495 | 38208264 | 29231 | 0.361 | 8.76e−04 |
| 516 | CHB | rs3744166 | ILMN_1781001 | ENSG00000184557 | *SOCS3* | 17 | 74482474 | 73867753 | 614721 | 0.492 | 3.59e−06 |
| 517 | LWK | rs3745164 | ILMN_1798083 | ENSG00000085872 | *CHERP* | 19 | 17198733 | 16514263 | 684470 | −0.364 | 7.72e−04 |
| 518 | YRI | rs3745194 | ILMN_1798083 | ENSG00000085872 | *CHERP* | 19 | 17313224 | 16514263 | 798961 | −0.352 | 1.89e−04 |
| 519 | JPT | rs3746185 | ILMN_1702507 | ENSG00000130517 | *PGPEP1* | 19 | 18335542 | 18312408 | 23134 | 0.392 | 2.75e−04 |
| 520 | MEX | rs3750203 | ILMN_1782021 | ENSG00000181135 | *ZNF707* | 8 | 144803169 | 144824516 | 21347 | 0.484 | 7.65e−04 |
| 521 | JPT | rs3757964 | ILMN_1811433 | ENSG00000161016 | *RPL8* | 8 | 145748909 | 145988609 | 239700 | 0.357 | 9.99e−04 |
| 522 | CEU | rs3757966 | ILMN_1811433 | ENSG00000161016 | *RPL8* | 8 | 145715426 | 145988609 | 273183 | −0.362 | 1.07e−04 |
| 523 | JPT | rs3760894 | ILMN_1654246 | ENSG00000077463 | *SIRT6* | 19 | 3921070 | 4133596 | 212526 | 0.383 | 3.83e−04 |
| 524 | JPT | rs3760895 | ILMN_1654246 | ENSG00000077463 | *SIRT6* | 19 | 3921435 | 4133596 | 212161 | 0.374 | 5.32e−04 |
| 525 | CHB | rs3760905 | ILMN_1654246 | ENSG00000077463 | *SIRT6* | 19 | 4133939 | 4133596 | 343 | 0.465 | 1.37e−05 |
| 526 | CHB | rs3760908 | ILMN_1654246 | ENSG00000077463 | *SIRT6* | 19 | 4135512 | 4133596 | 1916 | 0.465 | 1.60e−05 |
| 527 | GIH | rs3764383 | ILMN_1710082 | ENSG00000089685 | *BIRC5* | 17 | 73720446 | 73721872 | 1426 | 0.438 | 3.88e−05 |
| 528 | JPT | rs3764384 | ILMN_1654943 | ENSG00000089685 | *BIRC5* | 17 | 73719323 | 73721872 | 2549 | 0.376 | 5.05e−04 |
| 529 | GIH | rs3780032 | ILMN_1722390 | ENSG00000104472 | *CHRAC1* | 8 | 140764557 | 141590586 | 826029 | 0.4 | 1.94e−04 |
| 530 | CEU | rs3781942 | ILMN_1740240 | ENSG00000173599 | *PC* | 11 | 66893778 | 66482423 | 411355 | −0.325 | 6.00e−04 |
| 531 | GIH | rs3785496 | ILMN_1711327 | ENSG00000108395 | *TRIM37* | 17 | 53629132 | 54539011 | 909879 | 0.382 | 3.93e−04 |
| 532 | JPT | rs3787012 | ILMN_1669572 | ENSG00000070423 | *RNF126* | 19 | 612781 | 614227 | 1446 | −0.37 | 6.23e−04 |
| 533 | GIH | rs3793371 | ILMN_1769702 | ENSG00000197858 | *GPAA1* | 8 | 144735442 | 145209512 | 474070 | −0.379 | 4.47e−04 |
| 534 | MEX | rs3802199 | ILMN_1666022 | ENSG00000173530 | *TNFRSF10D* | 8 | 22323330 | 23077485 | 754155 | 0.478 | 8.92e−04 |
| 535 | MKK | rs3812436 | ILMN_1659651 | ENSG00000184428 | *TOP1MT* | 8 | 144483134 | 144488425 | 5291 | −0.354 | 2.05e−05 |
| 536 | MKK | rs3814772 | ILMN_1659651 | ENSG00000184428 | *TOP1MT* | 8 | 144536428 | 144488425 | 48003 | −0.366 | 1.19e−05 |
| 537 | JPT | rs3815904 | ILMN_1702507 | ENSG00000130517 | *PGPEP1* | 19 | 17363895 | 18312408 | 948513 | 0.358 | 9.54e−04 |
| 538 | CEU | rs3816732 | ILMN_1811433 | ENSG00000161016 | *RPL8* | 8 | 145695083 | 145988609 | 293526 | −0.368 | 8.13e−05 |
| 539 | CHB | rs3824217 | ILMN_1786345 | ENSG00000158941 | *KIAA1967* | 8 | 23175529 | 22518202 | 657327 | −0.374 | 6.28e−04 |
| 540 | GIH | rs3826700 | ILMN_1754923 | ENSG00000105085 | *MED26* | 19 | 17287501 | 16600015 | 687486 | 0.402 | 1.82e−04 |
| 541 | CEU | rs3866995 | ILMN_1811433 | ENSG00000161016 | *RPL8* | 8 | 146052971 | 145988609 | 64362 | −0.367 | 8.53e−05 |
| 542 | CEU | rs3882690 | ILMN_1811433 | ENSG00000161016 | *RPL8* | 8 | 146014266 | 145988609 | 25657 | 0.366 | 8.96e−05 |
| 543 | GIH | rs3923439 | ILMN_1699265 | ENSG00000120889 | *TNFRSF10B* | 8 | 22226024 | 22982637 | 756613 | 0.388 | 3.20e−04 |
| 544 | GIH | rs3936211 | ILMN_1811433 | ENSG00000161016 | *RPL8* | 8 | 145133612 | 145988609 | 854997 | 0.358 | 9.64e−04 |
| 545 | MKK | rs4076358 | ILMN_1659651 | ENSG00000184428 | *TOP1MT* | 8 | 144982227 | 144488425 | 493802 | 0.286 | 6.67e−04 |
| 546 | JPT | rs4102214 | ILMN_1685676 | ENSG00000162222 | *TTC9C* | 11 | 62969296 | 62252160 | 717136 | 0.389 | 3.33e−04 |
| 547 | CHB | rs412611 | ILMN_1781001 | ENSG00000184557 | *SOCS3* | 17 | 73643854 | 73867753 | 223899 | 0.4 | 2.36e−04 |
| 548 | LWK | rs41367351 | ILMN_1794823 | ENSG00000188171 | *ZNF626* | 19 | 20566187 | 20636246 | 70059 | −0.366 | 7.30e−04 |
| 549 | CHB | rs41421651 | ILMN_1717420 | ENSG00000104343 | *UBE2W* | 8 | 75199459 | 74923937 | 275522 | 0.394 | 2.99e−04 |
| 550 | CEU | rs418886 | ILMN_1752423 | ENSG00000126934 | *MAP2K2* | 19 | 3259076 | 4075126 | 816050 | −0.318 | 8.10e−04 |
| 551 | CHB | rs4242396 | ILMN_1786345 | ENSG00000158941 | *KIAA1967* | 8 | 23156852 | 22518202 | 638650 | −0.371 | 7.05e−04 |
| 552 | CHB | rs4242397 | ILMN_1786345 | ENSG00000158941 | *KIAA1967* | 8 | 23156908 | 22518202 | 638706 | 0.371 | 7.05e−04 |
| 553 | GIH | rs4242431 | ILMN_1699265 | ENSG00000120889 | *TNFRSF10B* | 8 | 22231478 | 22982637 | 751159 | 0.415 | 1.06e−04 |
| 554 | CEU | rs4244611 | ILMN_1811433 | ENSG00000161016 | *RPL8* | 8 | 145710732 | 145988609 | 277877 | −0.362 | 1.07e−04 |
| 555 | CEU | rs4244613 | ILMN_1811433 | ENSG00000161016 | *RPL8* | 8 | 145712573 | 145988609 | 276036 | −0.314 | 9.94e−04 |
| 556 | LWK | rs4246129 | ILMN_1722390 | ENSG00000104472 | *CHRAC1* | 8 | 141582254 | 141590586 | 8332 | 0.416 | 1.50e−04 |
| 557 | CEU | rs4251689 | ILMN_1811433 | ENSG00000161016 | *RPL8* | 8 | 145711938 | 145988609 | 276671 | 0.35 | 1.93e−04 |
| 558 | GIH | rs4266653 | ILMN_1699265 | ENSG00000120889 | *TNFRSF10B* | 8 | 22225032 | 22982637 | 757605 | −0.387 | 3.30e−04 |
| 559 | JPT | rs4268128 | ILMN_1714081 | ENSG00000008853 | *RHOBTB2* | 8 | 22993801 | 22913059 | 80742 | −0.381 | 4.06e−04 |
| 560 | GIH | rs4279640 | ILMN_1696752 | ENSG00000014164 | *ZC3H3* | 8 | 145502413 | 144694746 | 807667 | 0.384 | 3.74e−04 |
| 561 | CEU | rs4284750 | ILMN_1794823 | ENSG00000188171 | *ZNF626* | 19 | 19671050 | 20636246 | 965196 | −0.334 | 3.86e−04 |
| 562 | MKK | rs4316144 | ILMN_1717420 | ENSG00000104343 | *UBE2W* | 8 | 74475119 | 74923937 | 448818 | −0.289 | 5.74e−04 |
| 563 | MKK | rs4317621 | ILMN_1807501 | ENSG00000147536 | *GINS4* | 8 | 41635738 | 41505925 | 129813 | 0.346 | 3.26e−05 |
| 564 | GIH | rs4366053 | ILMN_1775573 | ENSG00000156162 | *DPY19L4* | 8 | 96706436 | 95801327 | 905109 | −0.407 | 1.46e−04 |
| 565 | GIH | rs4375723 | ILMN_1707391 | ENSG00000166263 | *STXBP4* | 17 | 49804242 | 50401125 | 596883 | 0.361 | 8.73e−04 |
| 566 | LWK | rs4380978 | ILMN_1703635 | ENSG00000104529 | *EEF1D* | 8 | 145570274 | 144750726 | 819548 | −0.386 | 3.41e−04 |
| 567 | MKK | rs4392912 | ILMN_1659651 | ENSG00000184428 | *TOP1MT* | 8 | 144459294 | 144488425 | 29131 | 0.323 | 1.35e−04 |
| 568 | CHB | rs4415303 | ILMN_1717420 | ENSG00000104343 | *UBE2W* | 8 | 74249632 | 74923937 | 674305 | 0.369 | 7.51e−04 |
| 569 | JPT | rs4421324 | ILMN_1714108 | ENSG00000164938 | *TP53INP1* | 8 | 95193232 | 96030770 | 837538 | −0.372 | 5.69e−04 |
| 570 | GIH | rs4430053 | ILMN_1725683 | ENSG00000172728 | *FUT10* | 8 | 33358523 | 33450206 | 91683 | −0.378 | 4.69e−04 |
| 571 | YRI | rs4442934 | ILMN_1794823 | ENSG00000188171 | *ZNF626* | 19 | 20648294 | 20636246 | 12048 | −0.369 | 9.22e−05 |
| 572 | GIH | rs4460355 | ILMN_1805221 | ENSG00000147533 | *GOLGA7* | 8 | 40735146 | 41467238 | 732092 | −0.374 | 5.29e−04 |
| 573 | MEX | rs447294 | ILMN_1802190 | ENSG00000129654 | *FOXJ1* | 17 | 71819720 | 71648966 | 170754 | −0.517 | 2.75e−04 |
| 574 | CEU | rs4484658 | ILMN_1714108 | ENSG00000164938 | *TP53INP1* | 8 | 96277902 | 96030770 | 247132 | −0.311 | 9.86e−04 |
| 575 | GIH | rs4492338 | ILMN_1775573 | ENSG00000156162 | *DPY19L4* | 8 | 96708759 | 95801327 | 907432 | 0.407 | 1.46e−04 |
| 576 | CHB | rs4495442 | ILMN_1712517 | ENSG00000185730 | *ZNF696* | 8 | 144405211 | 144444971 | 39760 | −0.362 | 9.84e−04 |
| 577 | CHB | rs450474 | ILMN_1654943 | ENSG00000089685 | *BIRC5* | 17 | 73640937 | 73721872 | 80935 | 0.378 | 5.88e−04 |
| 578 | GIH | rs4510862 | ILMN_1714081 | ENSG00000008853 | *RHOBTB2* | 8 | 22220576 | 22913059 | 692483 | 0.366 | 7.19e−04 |
| 579 | MKK | rs4512387 | ILMN_1806304 | ENSG00000197217 | *ENTPD4* | 8 | 23242944 | 23371115 | 128171 | 0.313 | 1.88e−04 |
| 580 | GIH | rs4525614 | ILMN_1656118 | ENSG00000178951 | *ZBTB7A* | 19 | 4066684 | 4017816 | 48868 | −0.377 | 4.77e−04 |
| 581 | JPT | rs4534122 | ILMN_1778673 | ENSG00000147533 | *GOLGA7* | 8 | 40681414 | 41467238 | 785824 | 0.361 | 8.72e−04 |
| 582 | GIH | rs4541108 | ILMN_1781001 | ENSG00000184557 | *SOCS3* | 17 | 74840204 | 73867753 | 972451 | 0.403 | 1.73e−04 |
| 583 | GIH | rs4565458 | ILMN_1699265 | ENSG00000120889 | *TNFRSF10B* | 8 | 22210078 | 22982637 | 772559 | 0.388 | 3.20e−04 |
| 584 | LWK | rs4567028 | ILMN_1786345 | ENSG00000158941 | *KIAA1967* | 8 | 21624290 | 22518202 | 893912 | 0.365 | 7.35e−04 |
| 585 | LWK | rs4581040 | ILMN_1676946 | ENSG00000070718 | *AP3M2* | 8 | 42151183 | 42129748 | 21435 | −0.456 | 1.68e−05 |
| 586 | GIH | rs4592695 | ILMN_1781001 | ENSG00000184557 | *SOCS3* | 17 | 74843242 | 73867753 | 975489 | 0.403 | 1.73e−04 |
| 587 | GIH | rs4606077 | ILMN_1804169 | ENSG00000179526 | *SHARPIN* | 8 | 144727897 | 145231128 | 503231 | 0.386 | 3.38e−04 |
| 588 | GIH | rs4610723 | ILMN_1722390 | ENSG00000104472 | *CHRAC1* | 8 | 141596488 | 141590586 | 5902 | 0.388 | 3.15e−04 |
| 589 | CEU | rs4610795 | ILMN_1811433 | ENSG00000161016 | *RPL8* | 8 | 145852141 | 145988609 | 136468 | −0.415 | 7.03e−06 |
| 590 | YRI | rs4733951 | ILMN_1705871 | ENSG00000085788 | *DDHD2* | 8 | 38488988 | 38208264 | 280724 | −0.314 | 9.49e−04 |
| 591 | MEX | rs4736168 | ILMN_1714364 | ENSG00000169398 | *PTK2* | 8 | 141364520 | 142080514 | 715994 | 0.556 | 7.28e−05 |
| 592 | CEU | rs4736790 | ILMN_1684694 | ENSG00000029534 | *ANK1* | 8 | 41015205 | 41873155 | 857950 | 0.341 | 2.89e−04 |
| 593 | YRI | rs4736824 | ILMN_1740752 | ENSG00000158669 | *AGPAT6* | 8 | 41999118 | 41554876 | 444242 | 0.332 | 4.44e−04 |
| 594 | YRI | rs4736829 | ILMN_1676946 | ENSG00000070718 | *AP3M2* | 8 | 42170311 | 42129748 | 40563 | −0.423 | 5.24e−06 |
| 595 | MKK | rs4737000 | ILMN_1807501 | ENSG00000147536 | *GINS4* | 8 | 41628884 | 41505925 | 122959 | 0.312 | 1.94e−04 |
| 596 | MEX | rs4737018 | ILMN_1740752 | ENSG00000158669 | *AGPAT6* | 8 | 41833519 | 41554876 | 278643 | 0.494 | 5.55e−04 |
| 597 | MKK | rs4737038 | ILMN_1676946 | ENSG00000070718 | *AP3M2* | 8 | 42130496 | 42129748 | 748 | −0.293 | 4.91e−04 |
| 598 | MEX | rs4738443 | ILMN_1704873 | ENSG00000154582 | *TCEB1* | 8 | 75370208 | 75046956 | 323252 | −0.498 | 5.84e−04 |
| 599 | MKK | rs4789073 | ILMN_1776088 | ENSG00000109065 | *NAT9* | 17 | 70043973 | 70284065 | 240092 | 0.298 | 4.31e−04 |
| 600 | LWK | rs4789155 | ILMN_1762071 | ENSG00000141219 | *C17orf80* | 17 | 69070014 | 68740953 | 329061 | 0.387 | 3.25e−04 |
| 601 | LWK | rs4789353 | ILMN_1762071 | ENSG00000141219 | *C17orf80* | 17 | 69214827 | 68740953 | 473874 | 0.372 | 6.86e−04 |
| 602 | YRI | rs4789470 | ILMN_1806037 | ENSG00000167900 | *TK1* | 17 | 73114698 | 73694726 | 580028 | 0.35 | 2.02e−04 |
| 603 | YRI | rs4789504 | ILMN_1806037 | ENSG00000167900 | *TK1* | 17 | 73347240 | 73694726 | 347486 | −0.323 | 6.55e−04 |
| 604 | CHB | rs4789908 | ILMN_1781001 | ENSG00000184557 | *SOCS3* | 17 | 74473654 | 73867753 | 605901 | −0.378 | 5.44e−04 |
| 605 | CHB | rs4789910 | ILMN_1781001 | ENSG00000184557 | *SOCS3* | 17 | 74471493 | 73867753 | 603740 | 0.403 | 2.56e−04 |
| 606 | LWK | rs4789933 | ILMN_1781001 | ENSG00000184557 | *SOCS3* | 17 | 74435021 | 73867753 | 567268 | −0.375 | 5.24e−04 |
| 607 | MKK | rs4789949 | ILMN_1806037 | ENSG00000167900 | *TK1* | 17 | 74655055 | 73694726 | 960329 | 0.319 | 2.07e−04 |
| 608 | LWK | rs4789979 | ILMN_1781001 | ENSG00000184557 | *SOCS3* | 17 | 74774551 | 73867753 | 906798 | −0.396 | 2.73e−04 |
| 609 | GIH | rs4790015 | ILMN_1781001 | ENSG00000184557 | *SOCS3* | 17 | 74861315 | 73867753 | 993562 | 0.387 | 3.55e−04 |
| 610 | GIH | rs4796827 | ILMN_1710082 | ENSG00000089685 | *BIRC5* | 17 | 74145560 | 73721872 | 423688 | 0.422 | 7.69e−05 |
| 611 | CEU | rs4807437 | ILMN_1752423 | ENSG00000126934 | *MAP2K2* | 19 | 3260021 | 4075126 | 815105 | 0.332 | 4.39e−04 |
| 612 | CHB | rs4807546 | ILMN_1654246 | ENSG00000077463 | *SIRT6* | 19 | 4133060 | 4133596 | 536 | −0.483 | 5.69e−06 |
| 613 | GIH | rs4871876 | ILMN_1806304 | ENSG00000197217 | *ENTPD4* | 8 | 23427983 | 23371115 | 56868 | 0.383 | 3.90e−04 |
| 614 | GIH | rs4871992 | ILMN_1699265 | ENSG00000120889 | *TNFRSF10B* | 8 | 22202330 | 22982637 | 780307 | 0.388 | 3.20e−04 |
| 615 | GIH | rs4871994 | ILMN_1699265 | ENSG00000120889 | *TNFRSF10B* | 8 | 22261983 | 22982637 | 720654 | 0.388 | 3.20e−04 |
| 616 | MKK | rs4872011 | ILMN_1699265 | ENSG00000120889 | *TNFRSF10B* | 8 | 22691521 | 22982637 | 291116 | 0.288 | 6.03e−04 |
| 617 | CHB | rs4872092 | ILMN_1786345 | ENSG00000158941 | *KIAA1967* | 8 | 23150362 | 22518202 | 632160 | 0.371 | 7.05e−04 |
| 618 | GIH | rs4872129 | ILMN_1806304 | ENSG00000197217 | *ENTPD4* | 8 | 23333586 | 23371115 | 37529 | −0.403 | 1.74e−04 |
| 619 | JPT | rs4872196 | ILMN_1806304 | ENSG00000197217 | *ENTPD4* | 8 | 23748012 | 23371115 | 376897 | −0.379 | 4.54e−04 |
| 620 | GIH | rs4872463 | ILMN_1699265 | ENSG00000120889 | *TNFRSF10B* | 8 | 22203278 | 22982637 | 779359 | −0.388 | 3.20e−04 |
| 621 | GIH | rs4872466 | ILMN_1714081 | ENSG00000008853 | *RHOBTB2* | 8 | 22220146 | 22913059 | 692913 | 0.366 | 7.19e−04 |
| 622 | GIH | rs4872471 | ILMN_1714081 | ENSG00000008853 | *RHOBTB2* | 8 | 22248637 | 22913059 | 664422 | −0.372 | 6.83e−04 |
| 623 | GIH | rs4872476 | ILMN_1714081 | ENSG00000008853 | *RHOBTB2* | 8 | 22266179 | 22913059 | 646880 | −0.397 | 2.25e−04 |
| 624 | GIH | rs4872477 | ILMN_1714081 | ENSG00000008853 | *RHOBTB2* | 8 | 22273766 | 22913059 | 639293 | 0.366 | 7.19e−04 |
| 625 | GIH | rs4873792 | ILMN_1769702 | ENSG00000197858 | *GPAA1* | 8 | 144553999 | 145209512 | 655513 | −0.394 | 2.52e−04 |
| 626 | MEX | rs4873815 | ILMN_1782021 | ENSG00000181135 | *ZNF707* | 8 | 144796206 | 144824516 | 28310 | 0.478 | 8.92e−04 |
| 627 | CEU | rs4874118 | ILMN_1695404 | ENSG00000160932 | *LY6E* | 8 | 144586782 | 144171274 | 415508 | −0.334 | 4.91e−04 |
| 628 | MKK | rs4874137 | ILMN_1724611 | ENSG00000184428 | *TOP1MT* | 8 | 144614303 | 144488425 | 125878 | 0.282 | 8.06e−04 |
| 629 | CEU | rs4925811 | ILMN_1811433 | ENSG00000161016 | *RPL8* | 8 | 145773255 | 145988609 | 215354 | −0.368 | 8.12e−05 |
| 630 | CEU | rs4925848 | ILMN_1811433 | ENSG00000161016 | *RPL8* | 8 | 146116506 | 145988609 | 127897 | 0.326 | 5.42e−04 |
| 631 | CEU | rs4925849 | ILMN_1811433 | ENSG00000161016 | *RPL8* | 8 | 146118298 | 145988609 | 129689 | −0.402 | 1.64e−05 |
| 632 | MEX | rs4930355 | ILMN_1736689 | ENSG00000173599 | *PC* | 11 | 65873487 | 66482423 | 608936 | 0.517 | 2.74e−04 |
| 633 | LWK | rs4930497 | ILMN_1736689 | ENSG00000173599 | *PC* | 11 | 67232177 | 66482423 | 749754 | −0.383 | 3.88e−04 |
| 634 | LWK | rs4961309 | ILMN_1722390 | ENSG00000104472 | *CHRAC1* | 8 | 141583366 | 141590586 | 7220 | −0.442 | 3.25e−05 |
| 635 | LWK | rs4961323 | ILMN_1722390 | ENSG00000104472 | *CHRAC1* | 8 | 141595413 | 141590586 | 4827 | 0.442 | 3.23e−05 |
| 636 | JPT | rs4961329 | ILMN_1722390 | ENSG00000104472 | *CHRAC1* | 8 | 142173996 | 141590586 | 583410 | −0.394 | 2.46e−04 |
| 637 | CHB | rs4977033 | ILMN_1690490 | ENSG00000181638 | *GLI4* | 8 | 143590947 | 144400484 | 809537 | 0.381 | 4.83e−04 |
| 638 | LWK | rs4977165 | ILMN_1745271 | ENSG00000178896 | *EXOSC4* | 8 | 145211715 | 145205510 | 6205 | 0.371 | 6.41e−04 |
| 639 | CHB | rs4977199 | ILMN_1782543 | ENSG00000104529 | *EEF1D* | 8 | 145479499 | 144750726 | 728773 | −0.383 | 4.46e−04 |
| 640 | CEU | rs5029824 | ILMN_1811433 | ENSG00000161016 | *RPL8* | 8 | 146014859 | 145988609 | 26250 | 0.366 | 8.96e−05 |
| 641 | CHB | rs510889 | ILMN_1778673 | ENSG00000147533 | *GOLGA7* | 8 | 41725136 | 41467238 | 257898 | −0.373 | 6.52e−04 |
| 642 | MEX | rs531784 | ILMN_1736689 | ENSG00000173599 | *PC* | 11 | 65821426 | 66482423 | 660997 | −0.484 | 7.51e−04 |
| 643 | JPT | rs612578 | ILMN_1794823 | ENSG00000188171 | *ZNF626* | 19 | 20635716 | 20636246 | 530 | −0.4 | 2.00e−04 |
| 644 | GIH | rs642612 | ILMN_1710082 | ENSG00000089685 | *BIRC5* | 17 | 74033121 | 73721872 | 311249 | −0.373 | 5.50e−04 |
| 645 | CHB | rs6431 | ILMN_1724611 | ENSG00000184428 | *TOP1MT* | 8 | 143990767 | 144488425 | 497658 | 0.364 | 9.10e−04 |
| 646 | CHB | rs6433 | ILMN_1724611 | ENSG00000184428 | *TOP1MT* | 8 | 143990642 | 144488425 | 497783 | −0.364 | 9.10e−04 |
| 647 | CEU | rs6468171 | ILMN_1725683 | ENSG00000172728 | *FUT10* | 8 | 33475616 | 33450206 | 25410 | −0.472 | 2.16e−07 |
| 648 | CEU | rs6468174 | ILMN_1725683 | ENSG00000172728 | *FUT10* | 8 | 33521482 | 33450206 | 71276 | −0.468 | 2.96e−07 |
| 649 | CEU | rs6468175 | ILMN_1725683 | ENSG00000172728 | *FUT10* | 8 | 33540795 | 33450206 | 90589 | −0.481 | 1.23e−07 |
| 650 | LWK | rs6471526 | ILMN_1813635 | ENSG00000164944 | *KIAA1429* | 8 | 96397214 | 95634864 | 762350 | −0.373 | 5.68e−04 |
| 651 | MEX | rs6472810 | ILMN_1717420 | ENSG00000104343 | *UBE2W* | 8 | 74992271 | 74923937 | 68334 | −0.515 | 2.91e−04 |
| 652 | CHB | rs6472819 | ILMN_1704873 | ENSG00000154582 | *TCEB1* | 8 | 75169384 | 75046956 | 122428 | −0.365 | 8.72e−04 |
| 653 | MKK | rs6474182 | ILMN_1789138 | ENSG00000169499 | *PLEKHA2* | 8 | 39841614 | 38877986 | 963628 | −0.282 | 8.09e−04 |
| 654 | MKK | rs6474373 | ILMN_1676946 | ENSG00000070718 | *AP3M2* | 8 | 41916851 | 42129748 | 212897 | −0.297 | 4.04e−04 |
| 655 | YRI | rs6501238 | ILMN_1710082 | ENSG00000089685 | *BIRC5* | 17 | 74181334 | 73721872 | 459462 | −0.322 | 7.52e−04 |
| 656 | MKK | rs6503915 | ILMN_1791106 | ENSG00000068097 | *HEATR6* | 17 | 54738981 | 55511074 | 772093 | 0.279 | 9.08e−04 |
| 657 | CEU | rs6503942 | ILMN_1791106 | ENSG00000068097 | *HEATR6* | 17 | 55477521 | 55511074 | 33553 | −0.314 | 8.84e−04 |
| 658 | JPT | rs6503943 | ILMN_1791106 | ENSG00000068097 | *HEATR6* | 17 | 55482786 | 55511074 | 28288 | 0.492 | 2.67e−06 |
| 659 | CEU | rs6503944 | ILMN_1791106 | ENSG00000068097 | *HEATR6* | 17 | 55494741 | 55511074 | 16333 | 0.314 | 8.84e−04 |
| 660 | CHB | rs6503958 | ILMN_1791106 | ENSG00000068097 | *HEATR6* | 17 | 55737950 | 55511074 | 226876 | 0.414 | 1.34e−04 |
| 661 | CHB | rs6503961 | ILMN_1791106 | ENSG00000068097 | *HEATR6* | 17 | 55803884 | 55511074 | 292810 | 0.458 | 1.96e−05 |
| 662 | CHB | rs6503965 | ILMN_1791106 | ENSG00000068097 | *HEATR6* | 17 | 55829097 | 55511074 | 318023 | −0.37 | 7.29e−04 |
| 663 | CHB | rs6503966 | ILMN_1791106 | ENSG00000068097 | *HEATR6* | 17 | 55857642 | 55511074 | 346568 | 0.398 | 2.52e−04 |
| 664 | YRI | rs6510985 | ILMN_1669572 | ENSG00000070423 | *RNF126* | 19 | 914695 | 614227 | 300468 | −0.322 | 8.69e−04 |
| 665 | YRI | rs6511148 | ILMN_1794823 | ENSG00000188171 | *ZNF626* | 19 | 20601016 | 20636246 | 35230 | 0.367 | 9.53e−05 |
| 666 | LWK | rs6512042 | ILMN_1708369 | ENSG00000127527 | *EPS15L1* | 19 | 15486172 | 16443768 | 957596 | 0.364 | 7.77e−04 |
| 667 | MKK | rs6557621 | ILMN_1666022 | ENSG00000173530 | *TNFRSF10D* | 8 | 23080615 | 23077485 | 3130 | −0.302 | 3.11e−04 |
| 668 | LWK | rs6557634 | ILMN_1714081 | ENSG00000008853 | *RHOBTB2* | 8 | 23116201 | 22913059 | 203142 | 0.375 | 5.21e−04 |
| 669 | GIH | rs6557678 | ILMN_1806304 | ENSG00000197217 | *ENTPD4* | 8 | 23438930 | 23371115 | 67815 | 0.37 | 6.19e−04 |
| 670 | LWK | rs6558295 | ILMN_1745271 | ENSG00000178896 | *EXOSC4* | 8 | 145211510 | 145205510 | 6000 | 0.383 | 3.84e−04 |
| 671 | LWK | rs6558297 | ILMN_1745271 | ENSG00000178896 | *EXOSC4* | 8 | 145215922 | 145205510 | 10412 | 0.377 | 5.13e−04 |
| 672 | LWK | rs6558321 | ILMN_1804169 | ENSG00000179526 | *SHARPIN* | 8 | 145331394 | 145231128 | 100266 | 0.412 | 1.20e−04 |
| 673 | MKK | rs6558337 | ILMN_1659651 | ENSG00000184428 | *TOP1MT* | 8 | 144393981 | 144488425 | 94444 | −0.304 | 3.69e−04 |
| 674 | MKK | rs6578111 | ILMN_1722390 | ENSG00000104472 | *CHRAC1* | 8 | 141589763 | 141590586 | 823 | −0.311 | 2.01e−04 |
| 675 | YRI | rs6583617 | ILMN_1676305 | ENSG00000169398 | *PTK2* | 8 | 142927999 | 142080514 | 847485 | −0.34 | 3.13e−04 |
| 676 | CEU | rs6599536 | ILMN_1811433 | ENSG00000161016 | *RPL8* | 8 | 145904495 | 145988609 | 84114 | 0.386 | 3.32e−05 |
| 677 | GIH | rs6651272 | ILMN_1727618 | ENSG00000156170 | *C8orf38* | 8 | 96117109 | 95977217 | 139892 | −0.4 | 1.96e−04 |
| 678 | MKK | rs6651422 | ILMN_1659651 | ENSG00000184428 | *TOP1MT* | 8 | 144448256 | 144488425 | 40169 | 0.283 | 7.80e−04 |
| 679 | LWK | rs6676680 | ILMN_1707337 | ENSG00000125459 | *MSTO1* | 1 | 152928827 | 153846631 | 917804 | 0.379 | 6.14e−04 |
| 680 | CEU | rs674499 | ILMN_1740240 | ENSG00000173599 | *PC* | 11 | 66720001 | 66482423 | 237578 | −0.349 | 2.82e−04 |
| 681 | CEU | rs676696 | ILMN_1781001 | ENSG00000184557 | *SOCS3* | 17 | 73958284 | 73867753 | 90531 | −0.362 | 1.09e−04 |
| 682 | GIH | rs6903 | ILMN_1754923 | ENSG00000105085 | *MED26* | 19 | 17278565 | 16600015 | 678550 | −0.366 | 7.09e−04 |
| 683 | LWK | rs6980609 | ILMN_1659651 | ENSG00000184428 | *TOP1MT* | 8 | 143862067 | 144488425 | 626358 | 0.38 | 4.27e−04 |
| 684 | LWK | rs6981680 | ILMN_1757230 | ENSG00000196150 | *ZNF250* | 8 | 146122283 | 146097650 | 24633 | 0.38 | 4.37e−04 |
| 685 | CEU | rs6982159 | ILMN_1725683 | ENSG00000172728 | *FUT10* | 8 | 33412011 | 33450206 | 38195 | −0.555 | 4.41e−10 |
| 686 | CEU | rs6983747 | ILMN_1778673 | ENSG00000147533 | *GOLGA7* | 8 | 40880943 | 41467238 | 586295 | −0.312 | 9.47e−04 |
| 687 | GIH | rs6983758 | ILMN_1659801 | ENSG00000155097 | *ATP6V1C1* | 8 | 103581961 | 104102445 | 520484 | 0.361 | 8.61e−04 |
| 688 | YRI | rs6985239 | ILMN_1725683 | ENSG00000172728 | *FUT10* | 8 | 33451456 | 33450206 | 1250 | 0.39 | 3.03e−05 |
| 689 | CEU | rs6989048 | ILMN_1725683 | ENSG00000172728 | *FUT10* | 8 | 33488624 | 33450206 | 38418 | 0.483 | 1.02e−07 |
| 690 | CEU | rs6989368 | ILMN_1811433 | ENSG00000161016 | *RPL8* | 8 | 145784265 | 145988609 | 204344 | 0.361 | 1.41e−04 |
| 691 | CEU | rs6993806 | ILMN_1725683 | ENSG00000172728 | *FUT10* | 8 | 33444713 | 33450206 | 5493 | 0.532 | 2.57e−09 |
| 692 | GIH | rs6993855 | ILMN_1699265 | ENSG00000120889 | *TNFRSF10B* | 8 | 22219742 | 22982637 | 762895 | −0.388 | 3.20e−04 |
| 693 | LWK | rs6995408 | ILMN_1714081 | ENSG00000008853 | *RHOBTB2* | 8 | 23117823 | 22913059 | 204764 | 0.399 | 2.23e−04 |
| 694 | CEU | rs6995454 | ILMN_1725683 | ENSG00000172728 | *FUT10* | 8 | 33423035 | 33450206 | 27171 | −0.473 | 2.01e−07 |
| 695 | LWK | rs6995534 | ILMN_1804169 | ENSG00000179526 | *SHARPIN* | 8 | 145327784 | 145231128 | 96656 | 0.412 | 1.20e−04 |
| 696 | CEU | rs6996294 | ILMN_1725683 | ENSG00000172728 | *FUT10* | 8 | 33557468 | 33450206 | 107262 | −0.438 | 1.95e−06 |
| 697 | YRI | rs6996541 | ILMN_1714364 | ENSG00000169398 | *PTK2* | 8 | 142169696 | 142080514 | 89182 | −0.321 | 7.15e−04 |
| 698 | CEU | rs6996562 | ILMN_1725683 | ENSG00000172728 | *FUT10* | 8 | 33511565 | 33450206 | 61359 | −0.42 | 5.38e−06 |
| 699 | MKK | rs6996628 | ILMN_1695404 | ENSG00000160932 | *LY6E* | 8 | 144975189 | 144171274 | 803915 | 0.28 | 8.77e−04 |
| 700 | CEU | rs7000195 | ILMN_1811433 | ENSG00000161016 | *RPL8* | 8 | 146114607 | 145988609 | 125998 | −0.404 | 1.33e−05 |
| 701 | LWK | rs7001472 | ILMN_1727618 | ENSG00000156170 | *C8orf38* | 8 | 96432591 | 95977217 | 455374 | 0.404 | 1.64e−04 |
| 702 | LWK | rs7001673 | ILMN_1722390 | ENSG00000104472 | *CHRAC1* | 8 | 142267778 | 141590586 | 677192 | −0.412 | 1.23e−04 |
| 703 | CEU | rs7001991 | ILMN_1676305 | ENSG00000169398 | *PTK2* | 8 | 142502439 | 142080514 | 421925 | 0.336 | 3.56e−04 |
| 704 | GIH | rs7003478 | ILMN_1699265 | ENSG00000120889 | *TNFRSF10B* | 8 | 22257068 | 22982637 | 725569 | −0.388 | 3.20e−04 |
| 705 | GIH | rs7003617 | ILMN_1725683 | ENSG00000172728 | *FUT10* | 8 | 33311183 | 33450206 | 139023 | −0.473 | 7.33e−06 |
| 706 | CEU | rs7004029 | ILMN_1811433 | ENSG00000161016 | *RPL8* | 8 | 145916846 | 145988609 | 71763 | −0.392 | 2.47e−05 |
| 707 | LWK | rs7004186 | ILMN_1745271 | ENSG00000178896 | *EXOSC4* | 8 | 145207715 | 145205510 | 2205 | 0.406 | 1.53e−04 |
| 708 | GIH | rs7004581 | ILMN_1714364 | ENSG00000169398 | *PTK2* | 8 | 143069975 | 142080514 | 989461 | −0.364 | 7.71e−04 |
| 709 | YRI | rs7004872 | ILMN_1759084 | ENSG00000164941 | *INTS8* | 8 | 95728818 | 95904710 | 175892 | 0.361 | 1.26e−04 |
| 710 | CEU | rs7005993 | ILMN_1699265 | ENSG00000120889 | *TNFRSF10B* | 8 | 22845367 | 22982637 | 137270 | −0.318 | 7.64e−04 |
| 711 | CEU | rs7006163 | ILMN_1725683 | ENSG00000172728 | *FUT10* | 8 | 33512592 | 33450206 | 62386 | 0.384 | 3.69e−05 |
| 712 | YRI | rs7008265 | ILMN_1811433 | ENSG00000161016 | *RPL8* | 8 | 146258870 | 145988609 | 270261 | 0.369 | 8.54e−05 |
| 713 | MKK | rs7009632 | ILMN_1676946 | ENSG00000070718 | *AP3M2* | 8 | 42127343 | 42129748 | 2405 | −0.293 | 4.91e−04 |
| 714 | GIH | rs7009973 | ILMN_1806304 | ENSG00000197217 | *ENTPD4* | 8 | 23430399 | 23371115 | 59284 | 0.383 | 3.90e−04 |
| 715 | CEU | rs7014773 | ILMN_1811433 | ENSG00000161016 | *RPL8* | 8 | 145943477 | 145988609 | 45132 | −0.583 | 2.85e−11 |
| 716 | LWK | rs7015048 | ILMN_1751328 | ENSG00000180921 | *FAM83H* | 8 | 145056271 | 144887902 | 168369 | −0.377 | 5.65e−04 |
| 717 | GIH | rs7016698 | ILMN_1813635 | ENSG00000164944 | *KIAA1429* | 8 | 95080308 | 95634864 | 554556 | −0.366 | 7.32e−04 |
| 718 | MKK | rs7016856 | ILMN_1805221 | ENSG00000147533 | *GOLGA7* | 8 | 40559570 | 41467238 | 907668 | −0.284 | 7.41e−04 |
| 719 | YRI | rs714925 | ILMN_1762071 | ENSG00000141219 | *C17orf80* | 17 | 68675264 | 68740953 | 65689 | 0.337 | 3.58e−04 |
| 720 | GIH | rs716317 | ILMN_1687703 | ENSG00000108395 | *TRIM37* | 17 | 54765423 | 54539011 | 226412 | −0.381 | 4.06e−04 |
| 721 | CEU | rs7210612 | ILMN_1791106 | ENSG00000068097 | *HEATR6* | 17 | 55516088 | 55511074 | 5014 | 0.314 | 8.84e−04 |
| 722 | MEX | rs7211960 | ILMN_1781001 | ENSG00000184557 | *SOCS3* | 17 | 74315703 | 73867753 | 447950 | 0.505 | 4.73e−04 |
| 723 | GIH | rs7212305 | ILMN_1781001 | ENSG00000184557 | *SOCS3* | 17 | 74845144 | 73867753 | 977391 | 0.373 | 5.60e−04 |
| 724 | MKK | rs7212590 | ILMN_1791106 | ENSG00000068097 | *HEATR6* | 17 | 55240491 | 55511074 | 270583 | 0.295 | 4.41e−04 |
| 725 | CEU | rs7212702 | ILMN_1791106 | ENSG00000068097 | *HEATR6* | 17 | 55495402 | 55511074 | 15672 | −0.314 | 8.84e−04 |
| 726 | GIH | rs7212846 | ILMN_1687703 | ENSG00000108395 | *TRIM37* | 17 | 54775202 | 54539011 | 236191 | −0.363 | 8.04e−04 |
| 727 | GIH | rs7214890 | ILMN_1781001 | ENSG00000184557 | *SOCS3* | 17 | 74839969 | 73867753 | 972216 | 0.403 | 1.73e−04 |
| 728 | LWK | rs7215765 | ILMN_1781001 | ENSG00000184557 | *SOCS3* | 17 | 74770188 | 73867753 | 902435 | −0.405 | 1.63e−04 |
| 729 | CEU | rs7215860 | ILMN_1791106 | ENSG00000068097 | *HEATR6* | 17 | 55538338 | 55511074 | 27264 | −0.314 | 8.84e−04 |
| 730 | CHB | rs7216167 | ILMN_1791106 | ENSG00000068097 | *HEATR6* | 17 | 55625877 | 55511074 | 114803 | −0.379 | 5.27e−04 |
| 731 | CHB | rs7216838 | ILMN_1791106 | ENSG00000068097 | *HEATR6* | 17 | 55735724 | 55511074 | 224650 | −0.414 | 1.34e−04 |
| 732 | MKK | rs7216862 | ILMN_1707391 | ENSG00000166263 | *STXBP4* | 17 | 50224657 | 50401125 | 176468 | 0.32 | 1.30e−04 |
| 733 | JPT | rs7217280 | ILMN_1791106 | ENSG00000068097 | *HEATR6* | 17 | 55517520 | 55511074 | 6446 | −0.494 | 2.78e−06 |
| 734 | JPT | rs7218114 | ILMN_1791106 | ENSG00000068097 | *HEATR6* | 17 | 55504859 | 55511074 | 6215 | −0.441 | 5.34e−05 |
| 735 | LWK | rs7218589 | ILMN_1654943 | ENSG00000089685 | *BIRC5* | 17 | 73622798 | 73721872 | 99074 | −0.386 | 4.07e−04 |
| 736 | CHB | rs7218904 | ILMN_1791106 | ENSG00000068097 | *HEATR6* | 17 | 55836280 | 55511074 | 325206 | −0.458 | 2.22e−05 |
| 737 | GIH | rs7219860 | ILMN_1711327 | ENSG00000108395 | *TRIM37* | 17 | 53676270 | 54539011 | 862741 | −0.36 | 9.05e−04 |
| 738 | CHB | rs7220716 | ILMN_1791106 | ENSG00000068097 | *HEATR6* | 17 | 55785079 | 55511074 | 274005 | −0.458 | 1.96e−05 |
| 739 | CHB | rs7221641 | ILMN_1791106 | ENSG00000068097 | *HEATR6* | 17 | 55848986 | 55511074 | 337912 | 0.458 | 1.96e−05 |
| 740 | YRI | rs7222118 | ILMN_1711327 | ENSG00000108395 | *TRIM37* | 17 | 54842320 | 54539011 | 303309 | −0.343 | 2.73e−04 |
| 741 | CHB | rs7223111 | ILMN_1791106 | ENSG00000068097 | *HEATR6* | 17 | 55733187 | 55511074 | 222113 | 0.414 | 1.34e−04 |
| 742 | CEU | rs7223281 | ILMN_1791106 | ENSG00000068097 | *HEATR6* | 17 | 55527229 | 55511074 | 16155 | −0.426 | 3.80e−06 |
| 743 | MKK | rs7224035 | ILMN_1711327 | ENSG00000108395 | *TRIM37* | 17 | 54727880 | 54539011 | 188869 | −0.305 | 2.79e−04 |
| 744 | MEX | rs7225739 | ILMN_1707391 | ENSG00000166263 | *STXBP4* | 17 | 50685040 | 50401125 | 283915 | 0.49 | 6.35e−04 |
| 745 | MKK | rs7226145 | ILMN_1776088 | ENSG00000109065 | *NAT9* | 17 | 70312022 | 70284065 | 27957 | 0.287 | 6.49e−04 |
| 746 | MKK | rs724037 | ILMN_1659651 | ENSG00000184428 | *TOP1MT* | 8 | 144473221 | 144488425 | 15204 | −0.319 | 1.38e−04 |
| 747 | YRI | rs7245503 | ILMN_1794823 | ENSG00000188171 | *ZNF626* | 19 | 20674089 | 20636246 | 37843 | −0.322 | 6.90e−04 |
| 748 | YRI | rs7248564 | ILMN_1754923 | ENSG00000105085 | *MED26* | 19 | 17338318 | 16600015 | 738303 | 0.353 | 2.09e−04 |
| 749 | LWK | rs7250811 | ILMN_1794823 | ENSG00000188171 | *ZNF626* | 19 | 20540614 | 20636246 | 95632 | 0.366 | 7.30e−04 |
| 750 | JPT | rs7251507 | ILMN_1794823 | ENSG00000188171 | *ZNF626* | 19 | 20588919 | 20636246 | 47327 | −0.497 | 2.02e−06 |
| 751 | LWK | rs7251627 | ILMN_1654246 | ENSG00000077463 | *SIRT6* | 19 | 4799593 | 4133596 | 665997 | −0.39 | 2.98e−04 |
| 752 | LWK | rs7254139 | ILMN_1794823 | ENSG00000188171 | *ZNF626* | 19 | 20654467 | 20636246 | 18221 | −0.366 | 7.13e−04 |
| 753 | LWK | rs7255631 | ILMN_1794823 | ENSG00000188171 | *ZNF626* | 19 | 20581096 | 20636246 | 55150 | 0.359 | 9.34e−04 |
| 754 | CHB | rs7255851 | ILMN_1669842 | ENSG00000167670 | *CHAF1A* | 19 | 4683167 | 4353660 | 329507 | 0.374 | 6.22e−04 |
| 755 | MKK | rs7255905 | ILMN_1794823 | ENSG00000188171 | *ZNF626* | 19 | 20580727 | 20636246 | 55519 | 0.289 | 5.82e−04 |
| 756 | LWK | rs7258003 | ILMN_1794823 | ENSG00000188171 | *ZNF626* | 19 | 20691124 | 20636246 | 54878 | 0.39 | 2.93e−04 |
| 757 | GIH | rs7260518 | ILMN_1752423 | ENSG00000126934 | *MAP2K2* | 19 | 4462955 | 4075126 | 387829 | 0.408 | 1.54e−04 |
| 758 | GIH | rs7357478 | ILMN_1699265 | ENSG00000120889 | *TNFRSF10B* | 8 | 22214095 | 22982637 | 768542 | −0.383 | 3.90e−04 |
| 759 | CHB | rs7361 | ILMN_1804789 | ENSG00000158941 | *KIAA1967* | 8 | 23175150 | 22518202 | 656948 | 0.409 | 1.66e−04 |
| 760 | MKK | rs7387207 | ILMN_1659651 | ENSG00000184428 | *TOP1MT* | 8 | 144429147 | 144488425 | 59278 | −0.278 | 9.83e−04 |
| 761 | YRI | rs7388266 | ILMN_1676305 | ENSG00000169398 | *PTK2* | 8 | 142928311 | 142080514 | 847797 | 0.34 | 3.13e−04 |
| 762 | JPT | rs740646 | ILMN_1791106 | ENSG00000068097 | *HEATR6* | 17 | 55496972 | 55511074 | 14102 | 0.408 | 1.42e−04 |
| 763 | MKK | rs740683 | ILMN_1686610 | ENSG00000011132 | *APBA3* | 19 | 3484803 | 3712673 | 227870 | −0.282 | 8.89e−04 |
| 764 | CEU | rs741844 | ILMN_1717420 | ENSG00000104343 | *UBE2W* | 8 | 75367850 | 74923937 | 443913 | −0.332 | 4.20e−04 |
| 765 | MEX | rs7430 | ILMN_1666022 | ENSG00000173530 | *TNFRSF10D* | 8 | 22454359 | 23077485 | 623126 | −0.483 | 7.85e−04 |
| 766 | JPT | rs745414 | ILMN_1654246 | ENSG00000077463 | *SIRT6* | 19 | 3925965 | 4133596 | 207631 | −0.375 | 5.25e−04 |
| 767 | MKK | rs7460258 | ILMN_1725683 | ENSG00000172728 | *FUT10* | 8 | 33079487 | 33450206 | 370719 | 0.282 | 7.94e−04 |
| 768 | YRI | rs7462044 | ILMN_1811433 | ENSG00000161016 | *RPL8* | 8 | 146239225 | 145988609 | 250616 | 0.313 | 9.58e−04 |
| 769 | CHB | rs7464232 | ILMN_1786345 | ENSG00000158941 | *KIAA1967* | 8 | 23159150 | 22518202 | 640948 | −0.371 | 7.05e−04 |
| 770 | JPT | rs747820 | ILMN_1669572 | ENSG00000070423 | *RNF126* | 19 | 245526 | 614227 | 368701 | 0.417 | 1.06e−04 |
| 771 | CEU | rs748193 | ILMN_1811433 | ENSG00000161016 | *RPL8* | 8 | 145652923 | 145988609 | 335686 | 0.369 | 7.84e−05 |
| 772 | CEU | rs748195 | ILMN_1811433 | ENSG00000161016 | *RPL8* | 8 | 145653322 | 145988609 | 335287 | −0.361 | 1.44e−04 |
| 773 | CEU | rs750472 | ILMN_1811433 | ENSG00000161016 | *RPL8* | 8 | 145672261 | 145988609 | 316348 | −0.335 | 3.72e−04 |
| 774 | GIH | rs753133 | ILMN_1805221 | ENSG00000147533 | *GOLGA7* | 8 | 40507475 | 41467238 | 959763 | −0.376 | 4.92e−04 |
| 775 | CHB | rs754291 | ILMN_1798083 | ENSG00000085872 | *CHERP* | 19 | 16561051 | 16514263 | 46788 | 0.361 | 9.89e−04 |
| 776 | CEU | rs754437 | ILMN_1725683 | ENSG00000172728 | *FUT10* | 8 | 33541076 | 33450206 | 90870 | 0.434 | 2.49e−06 |
| 777 | MEX | rs754696 | ILMN_1752333 | ENSG00000127526 | *SLC35E1* | 19 | 15763419 | 16544193 | 780774 | −0.491 | 9.70e−04 |
| 778 | CEU | rs756627 | ILMN_1811433 | ENSG00000161016 | *RPL8* | 8 | 145708094 | 145988609 | 280515 | −0.36 | 1.21e−04 |
| 779 | GIH | rs7812312 | ILMN_1727618 | ENSG00000156170 | *C8orf38* | 8 | 96089576 | 95977217 | 112359 | 0.4 | 1.96e−04 |
| 780 | CEU | rs7812958 | ILMN_1725683 | ENSG00000172728 | *FUT10* | 8 | 33526883 | 33450206 | 76677 | 0.515 | 1.01e−08 |
| 781 | GIH | rs7813318 | ILMN_1725683 | ENSG00000172728 | *FUT10* | 8 | 33389287 | 33450206 | 60919 | 0.414 | 1.48e−04 |
| 782 | GIH | rs7813840 | ILMN_1806304 | ENSG00000197217 | *ENTPD4* | 8 | 23411404 | 23371115 | 40289 | −0.422 | 7.85e−05 |
| 783 | GIH | rs7815717 | ILMN_1741841 | ENSG00000164944 | *KIAA1429* | 8 | 96448662 | 95634864 | 813798 | 0.388 | 3.14e−04 |
| 784 | GIH | rs7816555 | ILMN_1775573 | ENSG00000156162 | *DPY19L4* | 8 | 96712719 | 95801327 | 911392 | −0.413 | 1.15e−04 |
| 785 | YRI | rs7816830 | ILMN_1725683 | ENSG00000172728 | *FUT10* | 8 | 33460314 | 33450206 | 10108 | 0.39 | 3.03e−05 |
| 786 | YRI | rs7816891 | ILMN_1722390 | ENSG00000104472 | *CHRAC1* | 8 | 140821201 | 141590586 | 769385 | −0.356 | 1.59e−04 |
| 787 | YRI | rs7817915 | ILMN_1811433 | ENSG00000161016 | *RPL8* | 8 | 146244526 | 145988609 | 255917 | 0.317 | 9.25e−04 |
| 788 | MKK | rs7819278 | ILMN_1807501 | ENSG00000147536 | *GINS4* | 8 | 41703398 | 41505925 | 197473 | −0.315 | 1.64e−04 |
| 789 | MKK | rs7819636 | ILMN_1676946 | ENSG00000070718 | *AP3M2* | 8 | 42433839 | 42129748 | 304091 | −0.377 | 6.98e−06 |
| 790 | CEU | rs7821146 | ILMN_1715969 | ENSG00000147454 | *SLC25A37* | 8 | 23495847 | 23442308 | 53539 | −0.356 | 1.47e−04 |
| 791 | MKK | rs7821261 | ILMN_1806304 | ENSG00000197217 | *ENTPD4* | 8 | 23247428 | 23371115 | 123687 | −0.305 | 2.77e−04 |
| 792 | LWK | rs7822232 | ILMN_1745271 | ENSG00000178896 | *EXOSC4* | 8 | 145217675 | 145205510 | 12165 | 0.461 | 1.48e−05 |
| 793 | LWK | rs7822430 | ILMN_1796508 | ENSG00000184428 | *TOP1MT* | 8 | 144952912 | 144488425 | 464487 | −0.387 | 3.31e−04 |
| 794 | YRI | rs7822726 | ILMN_1811433 | ENSG00000161016 | *RPL8* | 8 | 146245512 | 145988609 | 256903 | −0.313 | 9.58e−04 |
| 795 | GIH | rs7823170 | ILMN_1741841 | ENSG00000164944 | *KIAA1429* | 8 | 96440135 | 95634864 | 805271 | −0.406 | 1.52e−04 |
| 796 | JPT | rs7823327 | ILMN_1786345 | ENSG00000158941 | *KIAA1967* | 8 | 22618297 | 22518202 | 100095 | −0.371 | 6.10e−04 |
| 797 | GIH | rs7823585 | ILMN_1806304 | ENSG00000197217 | *ENTPD4* | 8 | 23331729 | 23371115 | 39386 | 0.363 | 7.96e−04 |
| 798 | GIH | rs7823779 | ILMN_1725683 | ENSG00000172728 | *FUT10* | 8 | 33385822 | 33450206 | 64384 | 0.412 | 1.23e−04 |
| 799 | MEX | rs7825202 | ILMN_1740752 | ENSG00000158669 | *AGPAT6* | 8 | 41832771 | 41554876 | 277895 | −0.494 | 5.55e−04 |
| 800 | GIH | rs7826196 | ILMN_1727618 | ENSG00000156170 | *C8orf38* | 8 | 96098178 | 95977217 | 120961 | −0.4 | 1.96e−04 |
| 801 | CEU | rs7826247 | ILMN_1715969 | ENSG00000147454 | *SLC25A37* | 8 | 23454180 | 23442308 | 11872 | 0.32 | 7.05e−04 |
| 802 | CEU | rs7826311 | ILMN_1725683 | ENSG00000172728 | *FUT10* | 8 | 33483377 | 33450206 | 33171 | −0.483 | 1.02e−07 |
| 803 | GIH | rs7826414 | ILMN_1699265 | ENSG00000120889 | *TNFRSF10B* | 8 | 22237308 | 22982637 | 745329 | 0.383 | 3.90e−04 |
| 804 | LWK | rs7827093 | ILMN_1690546 | ENSG00000120910 | *PPP3CC* | 8 | 22390555 | 22354541 | 36014 | −0.421 | 8.03e−05 |
| 805 | MKK | rs7827271 | ILMN_1699473 | ENSG00000158941 | *KIAA1967* | 8 | 21641798 | 22518202 | 876404 | 0.284 | 7.25e−04 |
| 806 | CHB | rs7828303 | ILMN_1782543 | ENSG00000104529 | *EEF1D* | 8 | 145479936 | 144750726 | 729210 | 0.383 | 4.46e−04 |
| 807 | CEU | rs7829094 | ILMN_1715969 | ENSG00000147454 | *SLC25A37* | 8 | 23458220 | 23442308 | 15912 | 0.313 | 9.20e−04 |
| 808 | YRI | rs7830593 | ILMN_1699265 | ENSG00000120889 | *TNFRSF10B* | 8 | 23000640 | 22982637 | 18003 | −0.314 | 9.40e−04 |
| 809 | YRI | rs7832111 | ILMN_1804789 | ENSG00000158941 | *KIAA1967* | 8 | 22832159 | 22518202 | 313957 | −0.314 | 9.42e−04 |
| 810 | CEU | rs7833754 | ILMN_1699473 | ENSG00000158941 | *KIAA1967* | 8 | 23475795 | 22518202 | 957593 | 0.319 | 7.55e−04 |
| 811 | CEU | rs7833780 | ILMN_1811433 | ENSG00000161016 | *RPL8* | 8 | 145981915 | 145988609 | 6694 | −0.376 | 5.68e−05 |
| 812 | CEU | rs7834946 | ILMN_1722390 | ENSG00000104472 | *CHRAC1* | 8 | 141035897 | 141590586 | 554689 | −0.334 | 3.90e−04 |
| 813 | GIH | rs7835431 | ILMN_1714364 | ENSG00000169398 | *PTK2* | 8 | 142718373 | 142080514 | 637859 | 0.382 | 3.91e−04 |
| 814 | MKK | rs7836244 | ILMN_1676946 | ENSG00000070718 | *AP3M2* | 8 | 41866919 | 42129748 | 262829 | −0.309 | 2.21e−04 |
| 815 | CEU | rs7836942 | ILMN_1725683 | ENSG00000172728 | *FUT10* | 8 | 33500227 | 33450206 | 50021 | 0.532 | 2.57e−09 |
| 816 | LWK | rs7837375 | ILMN_1745271 | ENSG00000178896 | *EXOSC4* | 8 | 145250863 | 145205510 | 45353 | −0.359 | 9.24e−04 |
| 817 | GIH | rs7837764 | ILMN_1806304 | ENSG00000197217 | *ENTPD4* | 8 | 23431647 | 23371115 | 60532 | −0.425 | 6.87e−05 |
| 818 | GIH | rs7837994 | ILMN_1806304 | ENSG00000197217 | *ENTPD4* | 8 | 23429351 | 23371115 | 58236 | −0.383 | 3.90e−04 |
| 819 | CEU | rs7838624 | ILMN_1725683 | ENSG00000172728 | *FUT10* | 8 | 33585940 | 33450206 | 135734 | 0.312 | 9.57e−04 |
| 820 | GIH | rs7838717 | ILMN_1745271 | ENSG00000178896 | *EXOSC4* | 8 | 145475151 | 145205510 | 269641 | −0.357 | 9.78e−04 |
| 821 | CEU | rs7841080 | ILMN_1725683 | ENSG00000172728 | *FUT10* | 8 | 33547819 | 33450206 | 97613 | 0.444 | 1.34e−06 |
| 822 | CEU | rs7841518 | ILMN_1725683 | ENSG00000172728 | *FUT10* | 8 | 33483162 | 33450206 | 32956 | 0.483 | 1.02e−07 |
| 823 | CEU | rs7842778 | ILMN_1725683 | ENSG00000172728 | *FUT10* | 8 | 33510922 | 33450206 | 60716 | −0.395 | 2.11e−05 |
| 824 | YRI | rs7844641 | ILMN_1659651 | ENSG00000184428 | *TOP1MT* | 8 | 144456063 | 144488425 | 32362 | −0.347 | 2.35e−04 |
| 825 | MEX | rs7844699 | ILMN_1666022 | ENSG00000173530 | *TNFRSF10D* | 8 | 22304865 | 23077485 | 772620 | 0.485 | 7.32e−04 |
| 826 | GIH | rs7844784 | ILMN_1775573 | ENSG00000156162 | *DPY19L4* | 8 | 96553820 | 95801327 | 752493 | 0.372 | 5.69e−04 |
| 827 | MKK | rs7845253 | ILMN_1659651 | ENSG00000184428 | *TOP1MT* | 8 | 144470711 | 144488425 | 17714 | −0.336 | 5.87e−05 |
| 828 | CEU | rs7845367 | ILMN_1725683 | ENSG00000172728 | *FUT10* | 8 | 33529619 | 33450206 | 79413 | −0.49 | 6.48e−08 |
| 829 | YRI | rs7845738 | ILMN_1740752 | ENSG00000158669 | *AGPAT6* | 8 | 41997584 | 41554876 | 442708 | −0.332 | 4.44e−04 |
| 830 | YRI | rs7845953 | ILMN_1699265 | ENSG00000120889 | *TNFRSF10B* | 8 | 22884408 | 22982637 | 98229 | 0.34 | 3.23e−04 |
| 831 | CEU | rs7848 | ILMN_1725683 | ENSG00000172728 | *FUT10* | 8 | 33478272 | 33450206 | 28066 | −0.483 | 1.02e−07 |
| 832 | MEX | rs7925108 | ILMN_1736689 | ENSG00000173599 | *PC* | 11 | 65990599 | 66482423 | 491824 | −0.48 | 8.52e−04 |
| 833 | CHB | rs8064423 | ILMN_1791106 | ENSG00000068097 | *HEATR6* | 17 | 55716056 | 55511074 | 204982 | −0.429 | 7.04e−05 |
| 834 | CHB | rs8064576 | ILMN_1791106 | ENSG00000068097 | *HEATR6* | 17 | 55855046 | 55511074 | 343972 | −0.458 | 1.96e−05 |
| 835 | CHB | rs8064588 | ILMN_1791106 | ENSG00000068097 | *HEATR6* | 17 | 55716243 | 55511074 | 205169 | 0.432 | 6.28e−05 |
| 836 | GIH | rs8064769 | ILMN_1781001 | ENSG00000184557 | *SOCS3* | 17 | 74842633 | 73867753 | 974880 | −0.403 | 1.73e−04 |
| 837 | CHB | rs8064943 | ILMN_1710082 | ENSG00000089685 | *BIRC5* | 17 | 73243833 | 73721872 | 478039 | −0.367 | 8.14e−04 |
| 838 | GIH | rs8065479 | ILMN_1687703 | ENSG00000108395 | *TRIM37* | 17 | 54768829 | 54539011 | 229818 | −0.381 | 4.06e−04 |
| 839 | CEU | rs8066241 | ILMN_1791106 | ENSG00000068097 | *HEATR6* | 17 | 55511931 | 55511074 | 857 | −0.426 | 3.80e−06 |
| 840 | GIH | rs8066346 | ILMN_1707391 | ENSG00000166263 | *STXBP4* | 17 | 49874964 | 50401125 | 526161 | 0.361 | 8.73e−04 |
| 841 | LWK | rs8066768 | ILMN_1781001 | ENSG00000184557 | *SOCS3* | 17 | 74760386 | 73867753 | 892633 | −0.396 | 2.32e−04 |
| 842 | CHB | rs8067583 | ILMN_1791106 | ENSG00000068097 | *HEATR6* | 17 | 55741497 | 55511074 | 230423 | −0.414 | 1.34e−04 |
| 843 | JPT | rs8067909 | ILMN_1791106 | ENSG00000068097 | *HEATR6* | 17 | 55477409 | 55511074 | 33665 | −0.492 | 2.67e−06 |
| 844 | MEX | rs8067975 | ILMN_1710082 | ENSG00000089685 | *BIRC5* | 17 | 74703889 | 73721872 | 982017 | −0.493 | 5.84e−04 |
| 845 | CEU | rs8068376 | ILMN_1791106 | ENSG00000068097 | *HEATR6* | 17 | 55468702 | 55511074 | 42372 | −0.314 | 8.84e−04 |
| 846 | JPT | rs8070127 | ILMN_1654943 | ENSG00000089685 | *BIRC5* | 17 | 73708537 | 73721872 | 13335 | −0.388 | 3.10e−04 |
| 847 | CEU | rs8070466 | ILMN_1791106 | ENSG00000068097 | *HEATR6* | 17 | 55490062 | 55511074 | 21012 | 0.314 | 8.84e−04 |
| 848 | CEU | rs8071475 | ILMN_1791106 | ENSG00000068097 | *HEATR6* | 17 | 55328702 | 55511074 | 182372 | −0.316 | 8.14e−04 |
| 849 | GIH | rs8072709 | ILMN_1687703 | ENSG00000108395 | *TRIM37* | 17 | 54770329 | 54539011 | 231318 | 0.381 | 4.06e−04 |
| 850 | CHB | rs8074274 | ILMN_1791106 | ENSG00000068097 | *HEATR6* | 17 | 55857094 | 55511074 | 346020 | −0.458 | 1.96e−05 |
| 851 | LWK | rs8075002 | ILMN_1762071 | ENSG00000141219 | *C17orf80* | 17 | 69063313 | 68740953 | 322360 | −0.358 | 9.67e−04 |
| 852 | GIH | rs8075376 | ILMN_1781001 | ENSG00000184557 | *SOCS3* | 17 | 74862253 | 73867753 | 994500 | −0.385 | 3.55e−04 |
| 853 | LWK | rs8075789 | ILMN_1791106 | ENSG00000068097 | *HEATR6* | 17 | 55534954 | 55511074 | 23880 | 0.44 | 3.48e−05 |
| 854 | GIH | rs8077426 | ILMN_1711327 | ENSG00000108395 | *TRIM37* | 17 | 53635956 | 54539011 | 903055 | −0.374 | 5.33e−04 |
| 855 | CEU | rs8078068 | ILMN_1791106 | ENSG00000068097 | *HEATR6* | 17 | 55523766 | 55511074 | 12692 | 0.426 | 3.80e−06 |
| 856 | CHB | rs8078292 | ILMN_1791106 | ENSG00000068097 | *HEATR6* | 17 | 55744805 | 55511074 | 233731 | −0.414 | 1.34e−04 |
| 857 | CHB | rs8079797 | ILMN_1791106 | ENSG00000068097 | *HEATR6* | 17 | 55710411 | 55511074 | 199337 | 0.427 | 8.75e−05 |
| 858 | GIH | rs8081466 | ILMN_1781001 | ENSG00000184557 | *SOCS3* | 17 | 74842056 | 73867753 | 974303 | −0.36 | 8.89e−04 |
| 859 | CEU | rs8081973 | ILMN_1806037 | ENSG00000167900 | *TK1* | 17 | 74152185 | 73694726 | 457459 | −0.325 | 5.66e−04 |
| 860 | CEU | rs8082388 | ILMN_1791106 | ENSG00000068097 | *HEATR6* | 17 | 55485107 | 55511074 | 25967 | −0.314 | 8.84e−04 |
| 861 | LWK | rs8100147 | ILMN_1794823 | ENSG00000188171 | *ZNF626* | 19 | 20613255 | 20636246 | 22991 | −0.597 | 6.14e−09 |
| 862 | YRI | rs8100241 | ILMN_1798083 | ENSG00000085872 | *CHERP* | 19 | 17253894 | 16514263 | 739631 | −0.328 | 5.72e−04 |
| 863 | GIH | rs8100262 | ILMN_1794823 | ENSG00000188171 | *ZNF626* | 19 | 20613342 | 20636246 | 22904 | 0.397 | 2.20e−04 |
| 864 | GIH | rs8100448 | ILMN_1754923 | ENSG00000105085 | *MED26* | 19 | 17283402 | 16600015 | 683387 | 0.366 | 7.09e−04 |
| 865 | YRI | rs8100460 | ILMN_1794823 | ENSG00000188171 | *ZNF626* | 19 | 20644142 | 20636246 | 7896 | −0.368 | 9.46e−05 |
| 866 | JPT | rs8103146 | ILMN_1794823 | ENSG00000188171 | *ZNF626* | 19 | 20582566 | 20636246 | 53680 | 0.378 | 6.45e−04 |
| 867 | CHB | rs8103778 | ILMN_1752423 | ENSG00000126934 | *MAP2K2* | 19 | 4520809 | 4075126 | 445683 | 0.37 | 7.20e−04 |
| 868 | MKK | rs8104461 | ILMN_1686610 | ENSG00000011132 | *APBA3* | 19 | 4334783 | 3712673 | 622110 | −0.332 | 6.90e−05 |
| 869 | GIH | rs8105522 | ILMN_1673069 | ENSG00000142002 | *DPP9* | 19 | 5328037 | 4674875 | 653162 | −0.357 | 9.88e−04 |
| 870 | LWK | rs8108244 | ILMN_1794823 | ENSG00000188171 | *ZNF626* | 19 | 20654590 | 20636246 | 18344 | 0.55 | 8.79e−08 |
| 871 | LWK | rs8109263 | ILMN_1794823 | ENSG00000188171 | *ZNF626* | 19 | 20589484 | 20636246 | 46762 | −0.364 | 7.61e−04 |
| 872 | GIH | rs8178290 | ILMN_1711327 | ENSG00000108395 | *TRIM37* | 17 | 53675157 | 54539011 | 863854 | −0.36 | 9.05e−04 |
| 873 | YRI | rs8178793 | ILMN_1676946 | ENSG00000070718 | *AP3M2* | 8 | 42154137 | 42129748 | 24389 | 0.363 | 1.14e−04 |
| 874 | YRI | rs8178801 | ILMN_1676946 | ENSG00000070718 | *AP3M2* | 8 | 42153292 | 42129748 | 23544 | −0.363 | 1.14e−04 |
| 875 | YRI | rs8178811 | ILMN_1676946 | ENSG00000070718 | *AP3M2* | 8 | 42151149 | 42129748 | 21401 | 0.363 | 1.14e−04 |
| 876 | CEU | rs8685 | ILMN_1725683 | ENSG00000172728 | *FUT10* | 8 | 33477812 | 33450206 | 27606 | 0.532 | 2.57e−09 |
| 877 | GIH | rs872054 | ILMN_1805221 | ENSG00000147533 | *GOLGA7* | 8 | 40802495 | 41467238 | 664743 | 0.368 | 6.77e−04 |
| 878 | CEU | rs873884 | ILMN_1811433 | ENSG00000161016 | *RPL8* | 8 | 145726978 | 145988609 | 261631 | 0.388 | 3.41e−05 |
| 879 | LWK | rs877159 | ILMN_1654943 | ENSG00000089685 | *BIRC5* | 17 | 73040560 | 73721872 | 681312 | 0.365 | 7.39e−04 |
| 880 | YRI | rs877350 | ILMN_1714108 | ENSG00000164938 | *TP53INP1* | 8 | 96150578 | 96030770 | 119808 | 0.346 | 2.44e−04 |
| 881 | JPT | rs880190 | ILMN_1654943 | ENSG00000089685 | *BIRC5* | 17 | 73746610 | 73721872 | 24738 | 0.36 | 8.79e−04 |
| 882 | CHB | rs883429 | ILMN_1666022 | ENSG00000173530 | *TNFRSF10D* | 8 | 22942763 | 23077485 | 134722 | 0.363 | 9.41e−04 |
| 883 | CHB | rs884509 | ILMN_1714081 | ENSG00000008853 | *RHOBTB2* | 8 | 23198139 | 22913059 | 285080 | 0.379 | 5.22e−04 |
| 884 | MKK | rs884802 | ILMN_1776088 | ENSG00000109065 | *NAT9* | 17 | 70028638 | 70284065 | 255427 | −0.28 | 8.75e−04 |
| 885 | GIH | rs8887 | ILMN_1752423 | ENSG00000126934 | *MAP2K2* | 19 | 4453201 | 4075126 | 378075 | 0.36 | 8.85e−04 |
| 886 | GIH | rs890220 | ILMN_1676946 | ENSG00000070718 | *AP3M2* | 8 | 41597518 | 42129748 | 532230 | −0.387 | 3.34e−04 |
| 887 | GIH | rs892158 | ILMN_1752423 | ENSG00000126934 | *MAP2K2* | 19 | 4458716 | 4075126 | 383590 | 0.401 | 1.89e−04 |
| 888 | GIH | rs892160 | ILMN_1752423 | ENSG00000126934 | *MAP2K2* | 19 | 4450939 | 4075126 | 375813 | 0.359 | 9.32e−04 |
| 889 | MEX | rs896379 | ILMN_1666022 | ENSG00000173530 | *TNFRSF10D* | 8 | 22430145 | 23077485 | 647340 | −0.484 | 8.75e−04 |
| 890 | YRI | rs898670 | ILMN_1722390 | ENSG00000104472 | *CHRAC1* | 8 | 140846433 | 141590586 | 744153 | −0.368 | 8.74e−05 |
| 891 | JPT | rs9004 | ILMN_1811433 | ENSG00000161016 | *RPL8* | 8 | 146039126 | 145988609 | 50517 | 0.389 | 3.08e−04 |
| 892 | CEU | rs9071 | ILMN_1811433 | ENSG00000161016 | *RPL8* | 8 | 145721314 | 145988609 | 267295 | −0.338 | 3.21e−04 |
| 893 | GIH | rs907898 | ILMN_1781001 | ENSG00000184557 | *SOCS3* | 17 | 74857410 | 73867753 | 989657 | −0.409 | 1.34e−04 |
| 894 | MKK | rs924211 | ILMN_1659801 | ENSG00000155097 | *ATP6V1C1* | 8 | 104107331 | 104102445 | 4886 | 0.347 | 3.02e−05 |
| 895 | CEU | rs9297208 | ILMN_1725683 | ENSG00000172728 | *FUT10* | 8 | 33536588 | 33450206 | 86382 | −0.495 | 4.44e−08 |
| 896 | CEU | rs9297209 | ILMN_1725683 | ENSG00000172728 | *FUT10* | 8 | 33536800 | 33450206 | 86594 | 0.495 | 4.44e−08 |
| 897 | CHB | rs9297945 | ILMN_1813635 | ENSG00000164944 | *KIAA1429* | 8 | 95818199 | 95634864 | 183335 | 0.398 | 2.52e−04 |
| 898 | GIH | rs9297951 | ILMN_1727618 | ENSG00000156170 | *C8orf38* | 8 | 96110613 | 95977217 | 133396 | 0.4 | 1.96e−04 |
| 899 | YRI | rs9303008 | ILMN_1806037 | ENSG00000167900 | *TK1* | 17 | 73113718 | 73694726 | 581008 | 0.32 | 7.43e−04 |
| 900 | MKK | rs9303407 | ILMN_1711327 | ENSG00000108395 | *TRIM37* | 17 | 54731591 | 54539011 | 192580 | 0.284 | 7.48e−04 |
| 901 | CEU | rs9303417 | ILMN_1791106 | ENSG00000068097 | *HEATR6* | 17 | 55523104 | 55511074 | 12030 | −0.314 | 8.84e−04 |
| 902 | GIH | rs930571 | ILMN_1806037 | ENSG00000167900 | *TK1* | 17 | 74010287 | 73694726 | 315561 | −0.36 | 8.89e−04 |
| 903 | CEU | rs938641 | ILMN_1725683 | ENSG00000172728 | *FUT10* | 8 | 33416511 | 33450206 | 33695 | 0.464 | 3.78e−07 |
| 904 | GIH | rs950504 | ILMN_1794823 | ENSG00000188171 | *ZNF626* | 19 | 21575835 | 20636246 | 939589 | 0.357 | 9.99e−04 |
| 905 | GIH | rs954009 | ILMN_1725683 | ENSG00000172728 | *FUT10* | 8 | 33314997 | 33450206 | 135209 | −0.473 | 7.33e−06 |
| 906 | JPT | rs959419 | ILMN_1794823 | ENSG00000188171 | *ZNF626* | 19 | 20618532 | 20636246 | 17714 | −0.426 | 6.46e−05 |
| 907 | JPT | rs959420 | ILMN_1794823 | ENSG00000188171 | *ZNF626* | 19 | 20618518 | 20636246 | 17728 | −0.486 | 3.63e−06 |
| 908 | LWK | rs9644027 | ILMN_1666022 | ENSG00000173530 | *TNFRSF10D* | 8 | 22849899 | 23077485 | 227586 | 0.363 | 8.05e−04 |
| 909 | GIH | rs9656764 | ILMN_1725683 | ENSG00000172728 | *FUT10* | 8 | 33393941 | 33450206 | 56265 | 0.432 | 5.01e−05 |
| 910 | GIH | rs9656765 | ILMN_1725683 | ENSG00000172728 | *FUT10* | 8 | 33393968 | 33450206 | 56238 | −0.436 | 4.25e−05 |
| 911 | MEX | rs9676784 | ILMN_1752423 | ENSG00000126934 | *MAP2K2* | 19 | 3745099 | 4075126 | 330027 | 0.476 | 9.52e−04 |
| 912 | YRI | rs9694453 | ILMN_1663257 | ENSG00000155097 | *ATP6V1C1* | 8 | 104212297 | 104102445 | 109852 | 0.378 | 5.58e−05 |
| 913 | LWK | rs9694798 | ILMN_1659651 | ENSG00000184428 | *TOP1MT* | 8 | 144513368 | 144488425 | 24943 | −0.382 | 3.96e−04 |
| 914 | YRI | rs972354 | ILMN_1685676 | ENSG00000162222 | *TTC9C* | 11 | 61474183 | 62252160 | 777977 | 0.339 | 3.32e−04 |
| 915 | LWK | rs974588 | ILMN_1707391 | ENSG00000166263 | *STXBP4* | 17 | 50219263 | 50401125 | 181862 | −0.397 | 2.23e−04 |
| 916 | GIH | rs9886456 | ILMN_1741841 | ENSG00000164944 | *KIAA1429* | 8 | 96446296 | 95634864 | 811432 | −0.388 | 3.14e−04 |
| 917 | CHB | rs9890837 | ILMN_1791106 | ENSG00000068097 | *HEATR6* | 17 | 55657685 | 55511074 | 146611 | −0.414 | 1.34e−04 |
| 918 | CHB | rs9891296 | ILMN_1791106 | ENSG00000068097 | *HEATR6* | 17 | 55630776 | 55511074 | 119702 | −0.385 | 4.19e−04 |
| 919 | CHB | rs9893536 | ILMN_1791106 | ENSG00000068097 | *HEATR6* | 17 | 55663144 | 55511074 | 152070 | 0.424 | 1.35e−04 |
| 920 | CEU | rs9895182 | ILMN_1791106 | ENSG00000068097 | *HEATR6* | 17 | 55506667 | 55511074 | 4407 | −0.314 | 8.84e−04 |
| 921 | CHB | rs9897885 | ILMN_1791106 | ENSG00000068097 | *HEATR6* | 17 | 55764416 | 55511074 | 253342 | −0.414 | 1.34e−04 |
| 922 | GIH | rs9898046 | ILMN_1781001 | ENSG00000184557 | *SOCS3* | 17 | 74855102 | 73867753 | 987349 | 0.382 | 4.27e−04 |
| 923 | MKK | rs9900151 | ILMN_1707391 | ENSG00000166263 | *STXBP4* | 17 | 50504148 | 50401125 | 103023 | −0.295 | 4.52e−04 |
| 924 | JPT | rs9900396 | ILMN_1791106 | ENSG00000068097 | *HEATR6* | 17 | 55488106 | 55511074 | 22968 | 0.426 | 9.18e−05 |
| 925 | LWK | rs9901066 | ILMN_1791106 | ENSG00000068097 | *HEATR6* | 17 | 55743561 | 55511074 | 232487 | 0.458 | 1.55e−05 |
| 926 | JPT | rs9904341 | ILMN_1654943 | ENSG00000089685 | *BIRC5* | 17 | 73721962 | 73721872 | 90 | −0.376 | 5.05e−04 |
| 927 | MKK | rs9904374 | ILMN_1791106 | ENSG00000068097 | *HEATR6* | 17 | 55262878 | 55511074 | 248196 | −0.304 | 2.85e−04 |
| 928 | CEU | rs9907155 | ILMN_1791106 | ENSG00000068097 | *HEATR6* | 17 | 55513680 | 55511074 | 2606 | −0.314 | 8.84e−04 |
| 929 | MKK | rs9907660 | ILMN_1791106 | ENSG00000068097 | *HEATR6* | 17 | 55559629 | 55511074 | 48555 | −0.31 | 2.32e−04 |
| 930 | MEX | rs9911523 | ILMN_1781001 | ENSG00000184557 | *SOCS3* | 17 | 74193421 | 73867753 | 325668 | 0.479 | 8.67e−04 |
| 931 | CEU | rs9914853 | ILMN_1791106 | ENSG00000068097 | *HEATR6* | 17 | 55514084 | 55511074 | 3010 | −0.314 | 8.84e−04 |
| 932 | GIH | rs9915396 | ILMN_1762071 | ENSG00000141219 | *C17orf80* | 17 | 69079105 | 68740953 | 338152 | −0.382 | 3.93e−04 |
| 933 | MEX | rs9916134 | ILMN_1781001 | ENSG00000184557 | *SOCS3* | 17 | 73230856 | 73867753 | 636897 | −0.514 | 3.08e−04 |
| 934 | MKK | rs9941395 | ILMN_1791106 | ENSG00000068097 | *HEATR6* | 17 | 54731210 | 55511074 | 779864 | −0.279 | 9.08e−04 |
| 935 | YRI | rs998571 | ILMN_1762071 | ENSG00000141219 | *C17orf80* | 17 | 68676887 | 68740953 | 64066 | 0.314 | 9.46e−04 |
